# Solvation-Enhanced Salt Bridges

**DOI:** 10.1021/jacs.4c11869

**Published:** 2024-10-04

**Authors:** Ben Iddon, Christopher A. Hunter

**Affiliations:** Yusuf Hamied Department of Chemistry, University of Cambridge, Lensfield Road, Cambridge CB2 1EW, U.K.

## Abstract

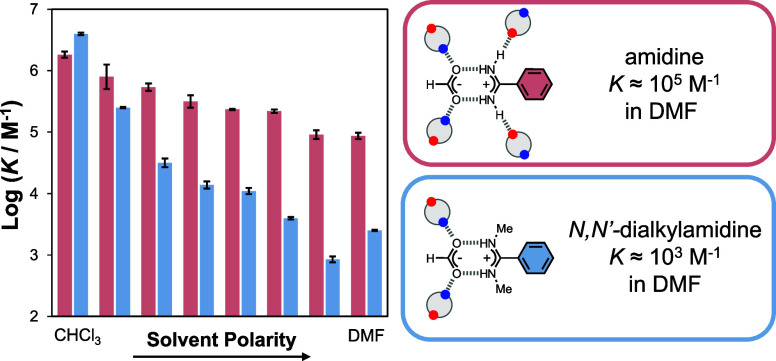

Salt bridges formed by amidines and carboxylic acids represent an important class of noncovalent interaction in biomolecular and supramolecular systems. Isothermal titration calorimetry was used to study the relationships between the strength of the interaction, the chemical structures of the components, and the nature of the solvent. The stability of the 1:1 complex formed in chloroform changed by 2 orders of magnitude depending on the basicity of the amidine and the acidity of the acid, which is consistent with proton transfer in the complex. Polar solvents reduce the stabilities of salt bridges formed with *N,N’*-dialkylamidines by up to 3 orders of magnitude, but this dependence on solvent polarity can be eliminated if the alkyl groups are replaced by protons in the parent amidine. The enhanced stability of the complex formed by benzamidine is due to solvation of the NH sites not directly involved in salt bridge formation, which become significantly more polar when proton transfer takes place, leading to more favorable interactions with polar solvents in the bound state. Calculation of H-bond parameters using density functional theory was used to predict solvent effects on the stabilities of salt bridges to within 1 kJ mol^–1^. While H-bonding interactions are strong in nonpolar solvents, and solvophobic interactions are strong in polar protic solvents, these interactions are weak in polar aprotic solvents. In contrast, amidinium–carboxylate salt bridges are stable in both polar and nonpolar aprotic solvents, which is attractive for the design of supramolecular systems that operate in different solvent environments.

## Introduction

**Figure 1:**
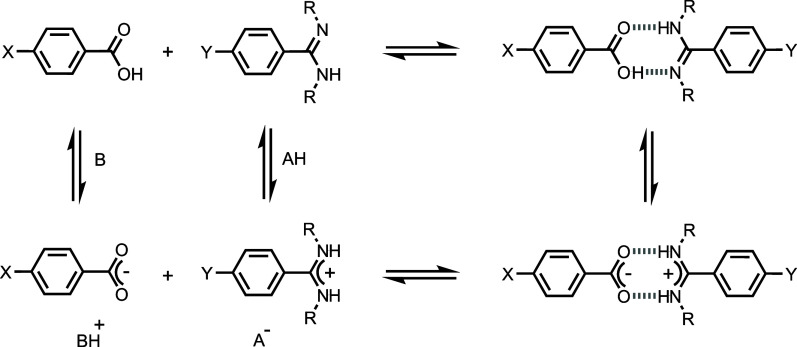
Salt bridge interaction between a benzoic acid and a benzamidine derivative. AH and B represent an acid and base, respectively, and X, Y, and R are substituents.

Salt bridges represent an important class of noncovalent interactions that involve both H-bonding and ion-pairing when a cationic H-bond donor interacts with an anionic H-bond acceptor. These interactions play a pivotal role in biological systems, particularly in protein folding, protein-nucleic acid recognition, and medicinal chemistry.^1−4^ The amidinium–carboxylate salt bridge has been widely used in synthetic supramolecular systems due to the large association constants found in nonpolar solvents and the well-defined geometry dictated by two cooperative H-bonds (Figure 1). Applications include sensing,^5,6^ crystal engineering,^7,8^ catalysis,^9^ H-bonded organic frameworks,^10^ polymer chemistry,^11,12^ self-replicating systems,^13^ self-assembly of duplexes and capsules,^14−17^ and template synthesis.^18,19^ In contrast to other noncovalent interactions that have been the subject of quantitative systematic studies,^20−23^ salt bridges have received relatively little attention. Reliable implementation of noncovalent chemistry in molecular design requires an understanding of the relationships between the strength of the interaction, the chemical structures of the components, and the nature of the solvent.^24^ Here, we use the formation of salt bridges between a series of carboxylic acid and benzamidine derivatives in a range of organic solvents to establish these principles.

The combination of H-bonding and ion-pairing involved in the formation of a salt bridge means that multiple equilibria may be involved, as illustrated in Figure 1. Proton transfer may take place to different extents before and after the formation of the H-bonds in the salt bridge, so there is a complex interplay of acid–base chemistry and solvation, as well as the effects of charge on the strength of the H-bonds.^25^ Here, we show that, starting from the neutral species (top left in Figure 1), the stability of the salt bridge interaction in chloroform can be modulated by 2 orders of magnitude depending on the X and Y substituents. Polar solvents reduce the stabilities of salt bridges formed with *N,N’*-dialkylamidines by up to 3 orders of magnitude, but this dependence on solvent polarity can be practically eliminated if the alkyl groups are replaced by protons in the parent amidine (R = H in Figure 1). We show that this unusual property of salt bridges formed by amidines and carboxylic acids is due to changes in the solvation shell associated with proton transfer that takes place within the salt bridge.

## Results and Discussion

### Synthesis

**Scheme 1:**
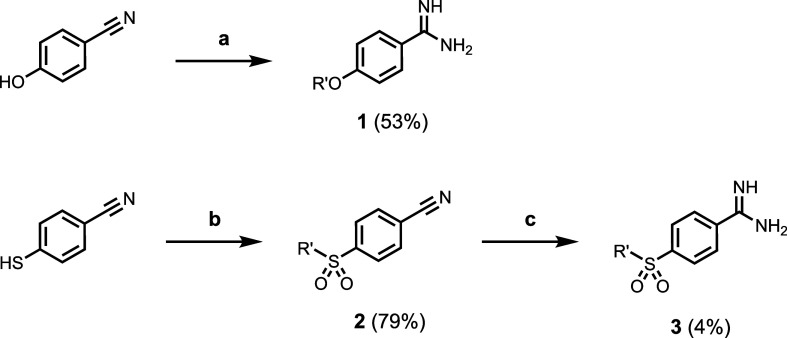
Synthesis of 4-Substituted Benzamidines (R’ = 2-Ethylhexyl) Conditions: (a) 1. 2-ethylhexyl bromide; 2. AcCl, MeOH then NH_3_; (b) 1. 2-ethylhexyl bromide; 2. *m*CPBA; (c) AcCl, MeOH then NH_3_.

All of the benzoic acids investigated were commercially available (X = H, NMe_2_, OMe, CF_3_, and NO_2_ in Figure 1). The synthesis of 4-substituted benzamidines was carried out by the routes shown in Scheme 1. 4*-*Hydroxybenzonitrile was first alkylated with 2-ethylhexyl bromide, and subsequent treatment with acetyl chloride in methanol followed by methanolic ammonia solution gave 4-alkoxybenzamidine **1**. 4*-*Mercaptobenzonitrile was similarly alkylated and then oxidized with 3-chloroperbenzoic acid (*m*CPBA) to give the 4-sulfonylbenzonitrile **2**. Conversion of **2** to the corresponding amidine gave 4-sulfonylbenzamidine **3**.

**Scheme 2:**
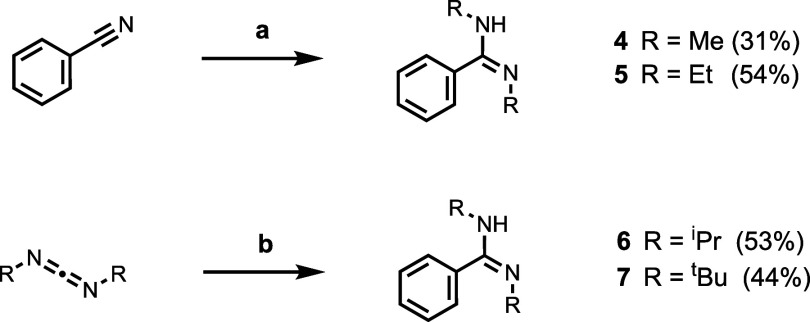
Synthesis of *N,N’*-Dialkylbenzamidines Conditions: (a) 1. ROTf; 2. RNH_2_; (b) phenyl magnesium bromide.

*N,N′-*Dialkylbenzamidines **4**–**7** were synthesized by the routes shown in Scheme 2. The *N,N′-*dimethyl and *N,N′-*diethyl derivatives **4** and **5** were prepared by alkylation of benzonitrile with the relevant alkyl triflate, followed by reaction with the corresponding primary amine. The *N,N′-*di-*i-*propyl and *N,N′-*di-*t*-butyl derivatives **6** and **7** were obtained by reaction of the corresponding carbodiimide with phenyl magnesium bromide.

### Effect of Aromatic Substituents

The interaction of pairwise combinations of benzoic acid and benzamidine derivatives was investigated in chloroform solution using isothermal titration calorimetry (ITC). In each case, the titration data fit well to a 1:1 binding isotherm (see Supporting Information for details), and the resulting thermodynamic parameters are summarized in Table 1.

**Table 1:** Substituent Effects on the Thermodynamic Parameters for Salt Bridge Formation between Benzoic Acid and Benzamidine Derivatives Determined by ITC in CHCl_3_ at 298 K^a^

X	Y^b^	R	Log (*K/*M^–1^)	Δ*G*°/kJ mol^–1^	Δ*H°*^c^/kJ mol^–1^	Δ*S*°^c^/J K^–1^ mol^–1^	*N*
H	H	H	6.01 ± 0.05	–34.3 ± 0.3	–70.0 ± 3.0	–120 ± 10	1.07 ± 0.06^d^
NMe_2_	H	H	5.10 ± 0.10	–29.1 ± 0.6	–60.0 ± 0.8	–104 ± 5	1.08 ± 0.06^d^
OMe	H	H	5.69 ± 0.03	–32.5 ± 0.2	–66.0 ± 3.0	–110 ± 10	1.10 ± 0.01^d^
CF_3_	H	H	6.51 ± 0.02	–37.1 ± 0.1	–76.0 ± 1.0	–130 ± 4	0.80 ± 0.01^d^
NO_2_	H	H	6.84 ± 0.09	–39.0 ± 0.5	–75.4 ± 0.6	–122 ± 4	0.90 ± 0.10^d^
H	OR’	H	6.55 ± 0.09	–37.4 ± 0.5	–70.0 ± 10.0	–110 ± 30	1.20 ± 0.20^d^
H	SO_2_R’	H	4.92 ± 0.03	–28.1 ± 0.2	–61.0 ± 1.0	–111 ± 4	0.90 ± 0.01^e^
H	H	Me	6.37 ± 0.05	–36.3 ± 0.3	–73.0 ± 9.0	–120 ± 30	1.20 ± 0.1^e^
H	H	Et	6.52 ± 0.09	–37.2 ± 0.5	–74.0 ± 3.0	–120 ± 10	1.20 ± 0.1^d^
H	H	iPr	6.40 ± 0.20	–37.0 ± 1.0	–75.0 ± 1.0	–128 ± 7	1.00 ± 0.1^e^
H	H	tBu	6.36 ± 0.08	–36.3 ± 0.5	–80.0 ± 3.0	–150 ± 10	1.17 ± 0.03^e^

a Errors are twice the standard deviation of at least two repeat measurements.

b R’ = 2-ethylhexyl.

c Errors in Δ*H*° and Δ*S*° do not take into account the uncertainty in *N*.

d *N* is the number of amidine molecules bound to one carboxylic acid in the complex.

e *N* is the number of carboxylic acid molecules bound to one amidine in the complex.

**Figure 2:**
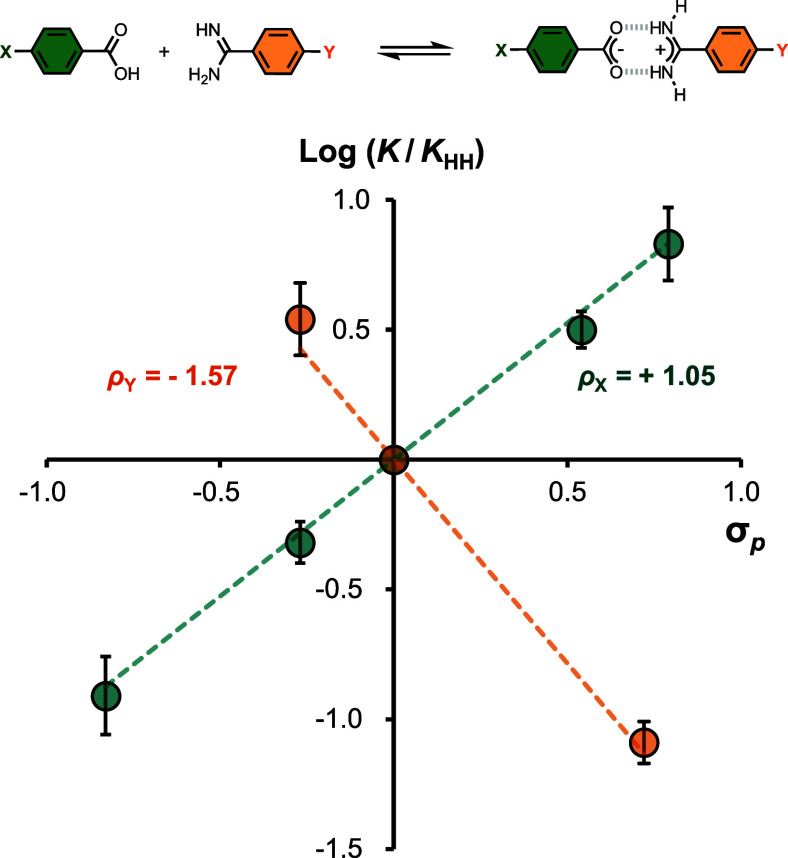
Hammett plots showing the relationship between the association constant for salt bridge formation measured in chloroform at 298 K and substituents on the benzoic acid (X, green) or the benzamidine (Y, orange). *K*_HH_ is the association constant for X = Y = H. The lines of best fit were fixed to pass through the origin and correspond to Log(*K*_X_/*K*_HH_) = 1.05 σ_X_ and Log(*K*_Y_/*K*_HH_) = −1.57 σ_Y_.

The nature of the aromatic substituents X and Y has a large impact on the stability of the 1:1 complex formed in chloroform, and the association constants span almost 2 orders of magnitude. The stability of the complex increases for electron-withdrawing groups on the carboxylic acid and electron-donating groups on the amidine. Figure 2 shows that the association constants correlate well with the Hammett substituent parameter σ, which measures the effect of substituents on the acidity of the corresponding benzoic acid. The results show that the strength of the interaction between a neutral carboxylic acid and a neutral amidine can be directly and predictably tuned by changing the acidity and/or basicity of the interacting partners.

The slopes of the Hammett plots in Figure 2, ρ, quantify the complexation-induced changes in charge on the carboxylic acid and amidine groups. The values of ρ are large and positive for X and large and negative for Y, which indicate a substantial change in charge for both partners when the salt bridge is formed. These observations suggest that a proton is transferred from the carboxylic acid to the amidine in the salt bridge, which exists in the zwitterionic form even in nonpolar solvents like chloroform. These results might be interpreted as a simple proton transfer reaction between the acid and amidine, generating the ionized species without forming an intermolecular complex. However, ^1^H NMR DOSY experiments in acetonitrile, a more polar solvent that would stabilize the separated ions better than chloroform, show that the complex is fully assembled in a 1:1 mixture at millimolar concentrations (see Supporting Information for details).

### Effect of *N*-Alkyl Amidine Substituents

**Figure 3:**
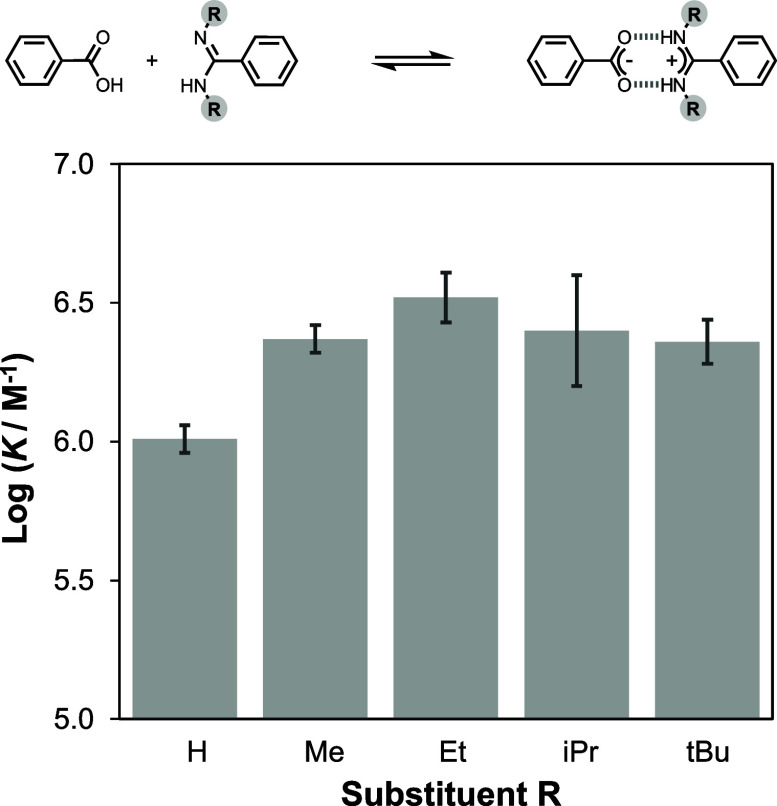
Effect of benzamidine *N,N’*-dialkyl substituents (R) on the association constant for salt bridge formation with benzoic acid measured in chloroform at 298 K.

*N,N′-*Dialkylamidines have been commonly used in supramolecular systems because they can easily be made from *N,N’*-dialkylcarbodiimides and show increased solubility in organic solvents compared with the parent amidines.^26^Figure 3 shows how the stability of the salt bridge formed with benzoic acid is affected by *N*-alkyl substituents (R) of increasing steric bulk in chloroform. Alkylation of the amidine increases the stability of the complex relative to the parent amidine (R = H), but the association constants measured for all four *N,N’*-dialkylamidines are the same within experimental error. The steric bulk of the alkyl groups does not play a role in determining the salt bridge stability. By analogy with the results for substituent effects described above, the increase in stability observed for the *N,N’*-dialkylamidines is most likely due to the higher basicity of more substituted nitrogen atoms.

### Solvent Effects

**Figure 4:**
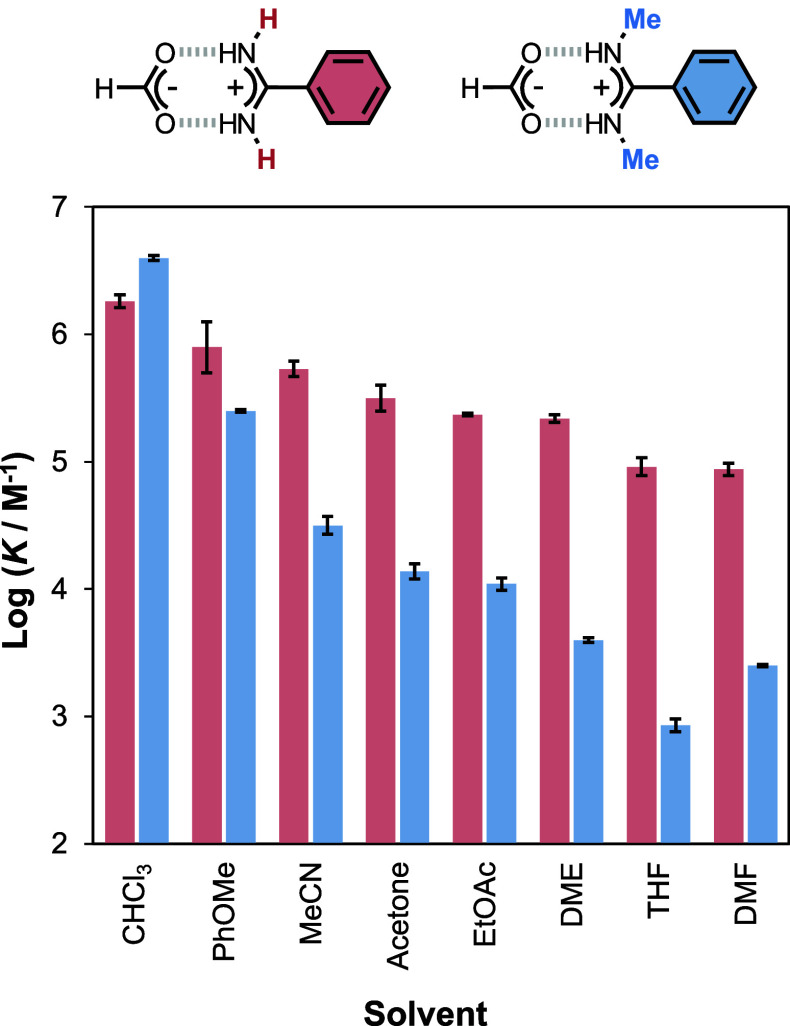
Solvent effects on the association constant for salt bridge formation between formic acid and benzamidine (red) or *N,N’*-dimethylbenzamidine (blue).

Although association constants for amidinium–carboxylate salt bridges have previously been measured in different solvents and in solvent mixtures,^16,27−31^ no systematic study has been attempted. To investigate the role of the solvent, ITC titrations were used to measure the interaction of formic acid with benzamidine (Y = H, R = H) and with *N,N′-*dimethylbenzamidine (Y = H, R = Me) in eight organic solvents with a wide range of polarities. Polar protic solvents were not included in these experiments because of the increased probability of proton transfer between solute and solvent, which would change the nature of the free species on the left-hand side of the equilibrium (for details, see eq 7 and the associated discussion later in the text). Table 2 summarizes the thermodynamic parameters obtained from the ITC experiments, and Figure 4 compares the stabilities of the complexes formed by the two different amidines. The association constant decreases with increasing solvent polarity for both benzamidine (red) and *N,N′-*dimethylbenzamidine (blue), but the behavior of the two systems is quite different. The benzamidinium–formate complex is much less sensitive to the solvent polarity than the *N,N’*-dimethylbenzamidinium–formate complex. Benzamidine forms a slightly less stable salt bridge than *N,N’*-dimethylbenzamidine in chloroform, but *N*-alkylation leads to a decrease in the association constant by 2 orders of magnitude in THF and DMF compared with the parent benzamidine.

**Table 2:** Solvent Effects on the Thermodynamic Parameters for Salt Bridge Formation between Formic Acid and Benzamidine Derivatives Determined by ITC at 298 K^a^

Solvent	R	Y	Log (*K/*M^–1^)	Δ*G*°/kJ mol^–1^	Δ*H***°**^b^/kJ mol^–1^	Δ*S***°**^b^ J/K^–1^ mol^–1^	*N*^c^
CHCl_3_	H	H	6.26 ± 0.05	–35.7 ± 0.3	–58.0 ± 1.0	–75 ± 4	1.01 ± 0.01
PhOMe	H	H	5.90 ± 0.20	–34.0 ± 1.0	–67.8 ± 0.1	–113 ± 4	0.80 ± 0.30
MeCN	H	H	5.73 ± 0.06	–32.7 ± 0.3	–63.0 ± 5.0	–102 ± 18	0.90 ± 0.20
Acetone	H	H	5.50 ± 0.10	–31.4 ± 0.6	–64.0 ± 2.0	–109 ± 9	0.89 ± 0.03
EtOAc	H	H	5.37 ± 0.01	–30.6 ± 0.1	–65.0 ± 4.0	–120 ± 10	0.89 ± 0.03
DME	H	H	5.34 ± 0.03	–30.5 ± 0.2	–68.0 ± 2.0	–126 ± 7	0.96 ± 0.04
THF	H	H	4.96 ± 0.07	–28.3 ± 0.4	–61.0 ± 0.4	–110 ± 3	0.79 ± 0.06
DMF	H	H	4.94 ± 0.05	–28.2 ± 0.3	–54.9 ± 0.3	–90 ± 2	0.97 ± 0.01
CHCl_3_	Me	H	6.60 ± 0.05	–37.7 ± 0.3	–60.0 ± 10	–75 ± 30	0.93 ± 0.03
PhOMe	Me	H	5.40 ± 0.01	–30.8 ± 0.1	–51.0 ± 0.1	–68 ± 1	0.88 ± 0.01
MeCN	Me	H	4.50 ± 0.07	–25.7 ± 0.4	–53.0 ± 5.0	–90 ± 20	0.95 ± 0.07
Acetone	Me	H	4.14 ± 0.06	–23.6 ± 0.3	–37.0 ± 2.0	–45 ± 8	1.03 ± 0.01
EtOAc	Me	H	4.04 ± 0.05	–23.0 ± 0.3	–44.0 ± 0.7	–70 ± 3	0.93 ± 0.04
DME	Me	H	3.60 ± 0.02	–20.5 ± 0.1	–46.0 ± 4.0	–90 ± 10	1.00^d^
THF	Me	H	2.93 ± 0.05	–16.7 ± 0.3	–23.0 ± 2.0	–21 ± 8	1.00^d^
DMF	Me	H	3.34 ± 0.07	–19.1 ± 0.4	–50.0 ± 10.0	–100 ± 30	1.00^d^

a Errors are twice the standard deviation of at least two repeat measurements.

b Errors do not take into account the uncertainty in *N*.

c *N* is the number of carboxylic acid molecules bound to one amidine in the complex.

d Titrations carried out in the low *c*-value regime were analyzed with *N* fixed at 1.00.^32^.

**Figure 5:**
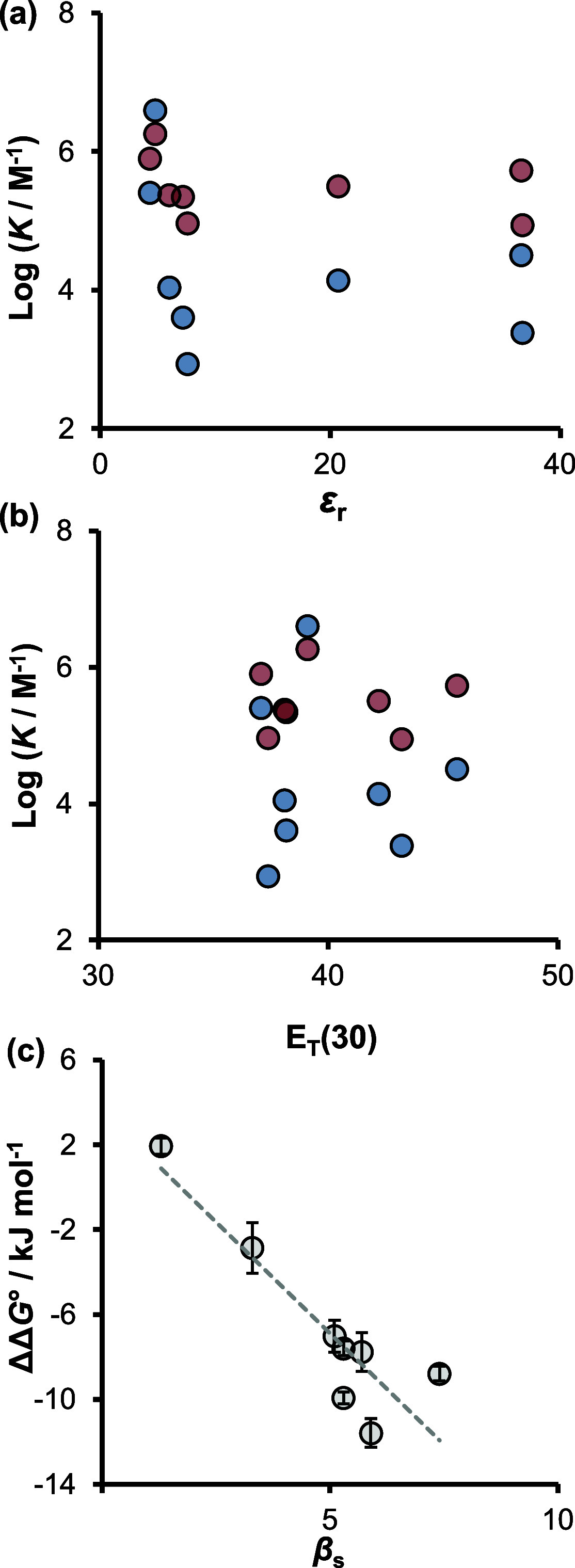
Comparison of the association constants for salt bridge formation between formic acid and benzamidine (red) or *N,N’*-dimethylbenzamidine (blue) with (a) solvent dielectric constant, *ε*_r_, and (b) solvent polarity, *E*_T_(30).^37^ (c) Comparison of the difference between the free energy changes for the formation of the benzamidine and *N,N’*-dimethylbenzamidine complexes (ΔΔ*G*°) with the solvent hydrogen bond acceptor parameter (β_S_); *R*^2^ = 0.81. (See the Supporting Information for details.)

No correlation was found between the association constants and common descriptors of the solvent polarity. For example, Figure 5a shows the relationship between the association constants and the solvent dielectric constant, ε_r_, which indicates that the ionizing power of the solvent is not an important factor governing the observed solvent effects. Figure 5b shows the relationship with solvent polarity parameter *E*_T_(30), which highlights the failure of bulk solvent descriptors to account for the observed solvent effects. Similar results were found for the Hansen solubility parameters (see Supporting Information). However, a correlation was found between the solvent hydrogen bond acceptor parameter, β_S_, and the difference between the free energy changes for the formation of the benzamidine and *N,N’*-dimethylbenzamidine complexes (ΔΔ*G*°, Figure 5c). The enhanced stability of the benzamidine complex in polar solvents is therefore related to interactions between solvent H-bond acceptors and H-bond donor sites in the benzamidine complex that are not present in the *N,N’*-dimethyl complex. Since the difference between the two complexes is simply the replacement of two NH H-bond donor sites with nonpolar methyl groups, this observation suggests that a more explicit analysis of the details of the solvation shell may shed light on the effect of solvent on salt bridge stability.

Solution-phase complexation can be described by a solvent competition model that uses the parameters α and β to quantify the noncovalent interaction properties of solute and solvent functional groups.^24,33−35^ These parameters can be determined by experiment or calculated using density functional theory (DFT) molecular electrostatic potential surfaces in conjunction with a footprinting algorithm described previously.^36^ The free energy contribution due to an intermolecular interaction between two functional groups is given in eq 1.
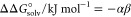
1

**Figure 6:**
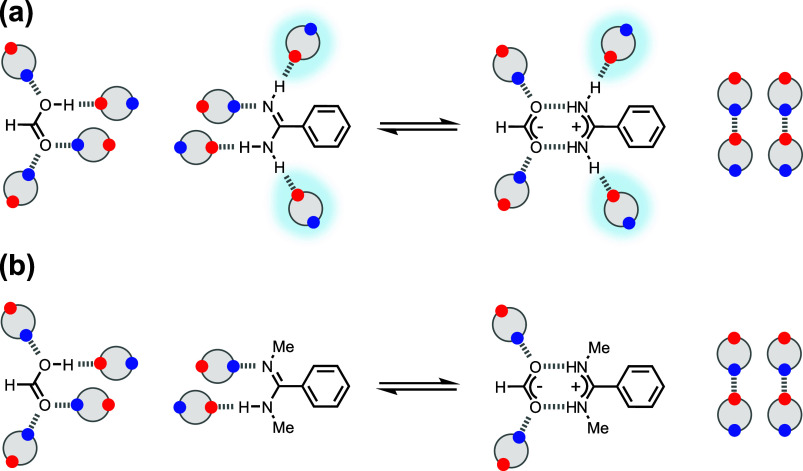
Primary solute–solvent interactions in the solvation shells of the free and bound species involved in the formation of (a) the benzamidinium–formate salt bridge and (b) the *N,N’*-dimethylbenzamidinium–formate salt bridge. The major difference is due to the solvation interactions highlighted in blue.

This approach can be used to build up a quantitative picture of the solvent–solute interactions that govern the behavior of the salt bridges. Figure 6 compares the primary interactions in the solvation shells of the free and bound species involved in the formation of the two different salt bridges. The major difference between the two complexes relates to the solvation of the two peripheral NH sites that are not directly involved in the solute–solute H-bonding interactions (the solvation interactions are highlighted in blue in Figure 6). These NH groups become significantly more polar in the zwitterionic complex compared with the neutral free state, which will lead to stronger interactions with the solvent in the bound state. It is the change in solvation of these peripheral sites that accounts for the difference in behavior between benzamidine and *N,N’*-dimethylbenzamidine. Stronger solvation of the peripheral NH protons upon formation of the salt bridge enhances the stability of the benzamidine complex by almost 2 orders of magnitude in DMF compared with the *N,N’*-dimethylbenzamidine complex.

The change in solvation free energy between free and bound species on formation of the salt bridge (ΔΔ*G*°_solv_) can be calculated in terms of the H-bond parameters for the solvent and solute by summing the free energy contributions of each pairwise interaction shown in Figure 6 (eq 2).
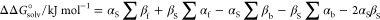
2where α_f_, β_f_, α_b_, and β_b_ are the H-bond parameters of the sites on the free and bound solutes, and α_S_ and β_S_ are the H-bond parameters of the solvent.

**Figure 7:**
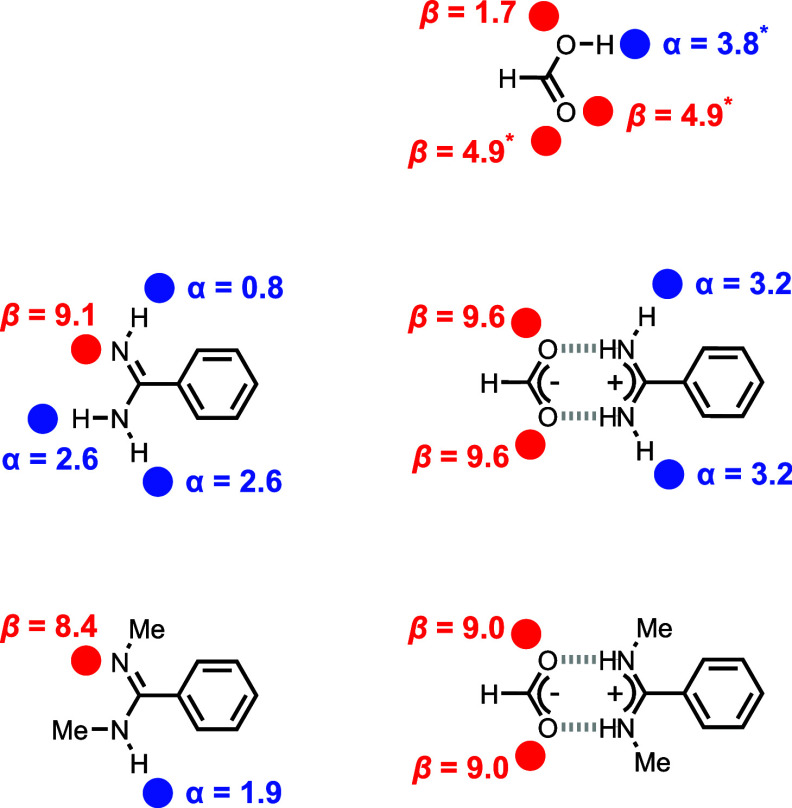
Free and bound solute H-bond parameters calculated using DFT (the carboxylic acid parameters labeled with an asterisk were obtained from experimental data).^35,36^

H-bond parameters for all of the sites on the free and bound solutes were calculated or obtained from experimental data (Figure 7). The calculated H-bond parameters confirm that the peripheral NH protons and oxygen lone pairs that are not directly involved in the salt bridge H-bonds become significantly better hydrogen bond donors and acceptors, respectively, when the proton is transferred in the salt bridge. Using these H-bond parameters in eq 2, it is possible to estimate how differences in solvation energy affect the relative stability of the two different salt bridges (eqs 3 and 4).
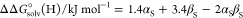
3
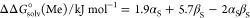
4

**Figure 8:**
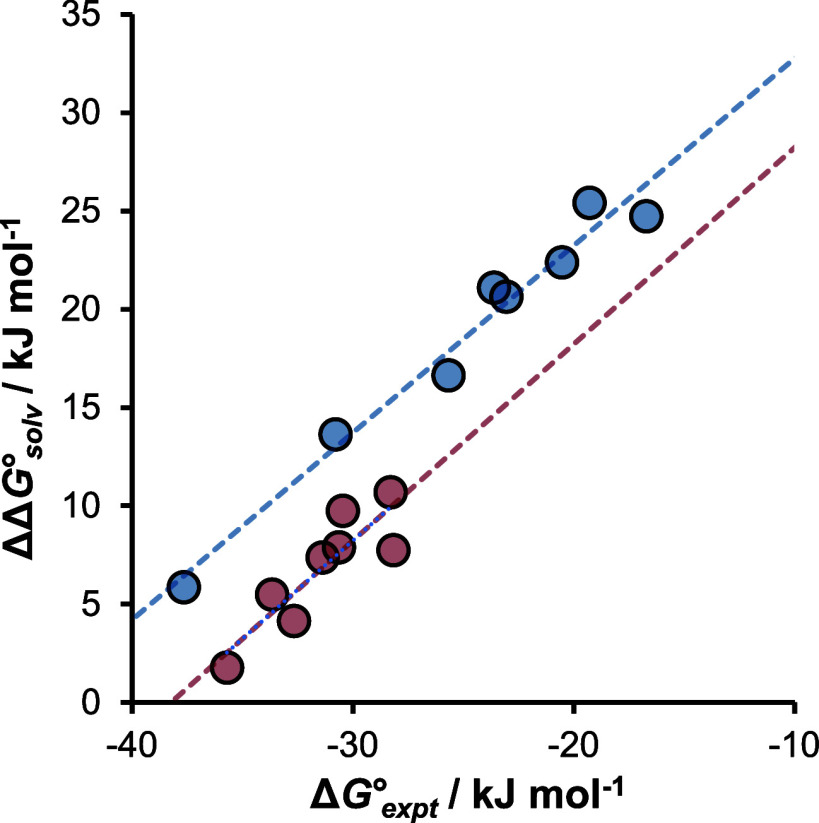
Comparison of the experimental free energy changes for the formation of salt bridges in different solvents with the associated change in solvation free energy calculated using eqs 3 and 4 (ΔΔ*G*°_solv_). The lines of best fit are Δ*G*°_expt_ = 38.2 + 1.00 ΔΔ*G*°_solv_ (R = H, red, RMSE = 1 kJ mol^–1^) and Δ*G*°_expt_ = 42.3 + 0.95 ΔΔ*G*°_solv_ (R = Me, blue, RMSE = 1 kJ mol^–1^).

The coefficients of the solvent H-bond parameters in eqs 3 and 4 predict that the *N,N’*-dimethyl complex should be significantly more sensitive to solvent polarity than the complex formed with the parent benzamidine, particularly with respect to the H-bond acceptor properties of the solvent, which is consistent with the experimental observations described above. Figure 8 compares the change in solvation energy predicted by eqs 3 and 4 (see Supporting Information for solvent H-bond parameters) with the experimental values of the free energy change for the formation of the salt bridge in different solvents. There is an excellent correlation for both the benzamidine (R = H, red) and *N,N’*-dimethylbenzamidine (R = Me, blue) complexes, and the slope of the line of best fit is 1.0 in both cases. In other words, the experimentally observed solvent effects on the stability of the salt bridge interactions are almost perfectly described by the primary solvation model in Figure 6.

**Figure 9:**
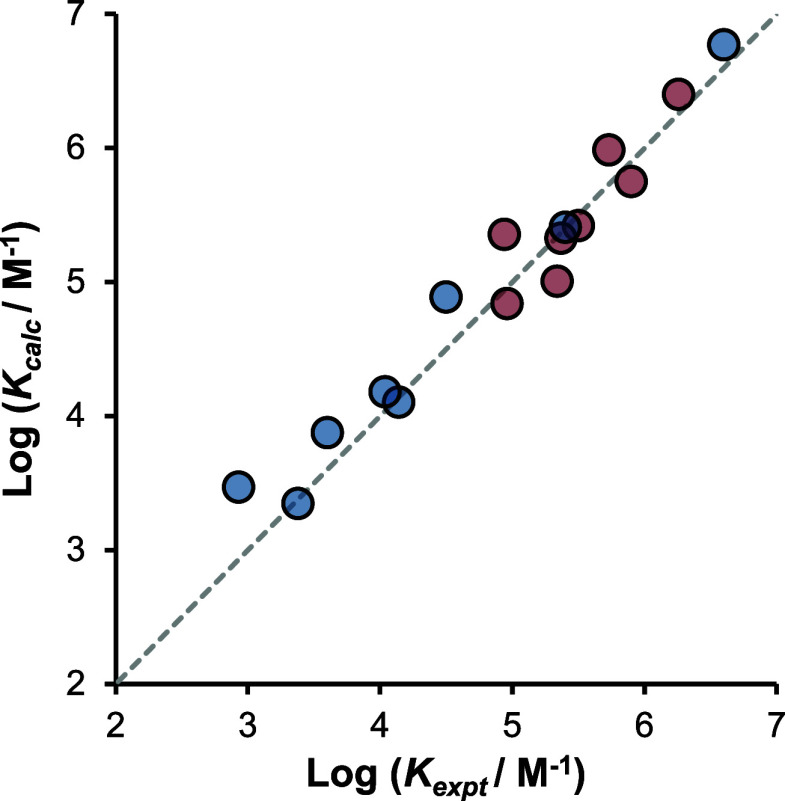
Comparison of experimentally determined association constants for the formation of salt bridges with formic acid in different solvents (*K*_expt_) with the corresponding values calculated using eqs 5 and 6 (*K*_calc_): benzamidine complex in red, and *N,N’*-dimethylbenzamidine complex in blue. The dashed line is *y = x* (RMSE = 0.25).

The intercepts on the Δ*G*°_expt_ axis in Figure 8 represent the intrinsic stabilities of the salt bridges in a completely nonpolar solvent (i.e., ΔΔ*G*°_solv_ = 0) and give −38 kJ mol^–1^ for the benzamidinium–formate complex and −44 kJ mol^–1^ for the *N,N’*-dimethylbenzamidinium–formate complex. Using the change in solvation energy from eqs 3 and 4 together with these values allows prediction of the stability of the salt bridge relative to the neutral carboxylic acid and amidine in any solvent for which the H-bond parameters are available (eqs 5 and 6). The only caveat is that the solutes should not be significantly ionized in the free state; otherwise, the competing equilibria shown in Figure 1 would complicate the analysis. Figure 9 compares the association constants calculated using eqs 5 and 6 with those obtained experimentally.
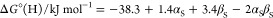
5
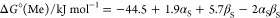
6

Equation 5 predicts that the association constant for the benzamidinium–formate salt bridge should be 6 × 10^7^ M^–1^ in water (α_S_ = 2.8, β_S_ = 4.5). However, experimental measurements for similar systems, e.g., the guanidinium–acetate salt bridge, show that salt bridges are much less stable in water (*K* < 1 M^–1^).^38,39^ The discrepancy comes from differences in the nature of the free species. The experiments described here were all carried out in organic solvents in which ionization of the free carboxylic acid and free amidine is negligible. In water, the ionized species are substantially populated in the free state (Figure 1), and these competing equilibria must be considered in the estimation of the overall association constant for the formation of the salt bridge. The association constant for the formation of a salt bridge (*K*) can be expressed in terms of the equilibrium constants for protonation of the amidine (*K*_A_), deprotonation of the acid (*K*_C_), and formation of the salt bridge starting from the neutral species (*K*_N_) (eq 7).
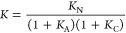
7

The equilibrium constants for ionization of the free species in water can be calculated from the acidity constants of benzamidinium (p*K*_a_ = 11.6)^40^ and formic acid (p*K*_a_ = 3.8)^41^ giving *K*_A_ = 4.0 × 10^4^ and *K*_C_ = 1.6 × 10^3^ at pH 7. Using these values in eq 7 together with the association constant predicted by using eq 5 (*K*_N_) gives an association constant of *K* = 0.9 M^–1^ for the benzamidinium–formate salt bridge in water at pH 7, which is consistent with the experimental observations. The difference between the behavior in water and in organic solvents lies in the ability of water to strongly solvate both anions and cations in the free state, whereas polar aprotic solvents solvate anions poorly.

Analogous behavior is observed in aprotic solvents if salts of the acid and amidine are used as the starting materials instead of the neutral species. For example, the association constant for the salt bridge formed on mixing tetrabutylammonium benzoate and benzamidinium chloride in dimethyl sulfoxide (DMSO) is 2,500 M^–1^.^7^ Although we have not measured association constants in DMSO, it is possible to predict the value with eq 5 using α_S_*=* 1.4 and β_S_ = 8.6. The calculated association constant for the formation of the benzamidinium–formate complex from the neutral species is 3 × 10^5^ M^–1^. The experimental value reported above is 2 orders of magnitude lower due to the two competing ion-pair equilibria in the free state, analogous to the competing ionization equilibria described by eq 7.

## Conclusions

A systematic investigation of factors affecting the strength of the amidinium–carboxylate salt bridge was carried out by measuring association constants for 27 different systems using isothermal titration calorimetry. Aromatic substituent effects show that electron-rich amidines and electron-poor carboxylic acids form the most stable complexes, suggesting that there is extensive proton transfer on the salt bridge formation. The steric size of the alkyl substituents on the nitrogen atoms of the amidine does not affect the stability of the salt bridge.

The results show that the solvent plays an important role in determining the stability of salt bridges, and the effects cannot be explained with bulk solvent descriptors. The complex of formic acid with *N,N’*-dimethylbenzamidine shows surprisingly different behavior compared to to the corresponding complex formed with benzamidine. In chloroform, the presence of methyl groups increases the stability of the salt bridge slightly. In more polar solvents, there is a decrease of 3 orders of magnitude in the stability of the *N,N’*-dimethylbenzamidine complex, whereas the association constant measured for the formation of the benzamidinium–formate salt bridge is between 10^5^ and 10^6^ M^–1^ in eight different solvents, ranging in polarity from chloroform to dimethylformamide.

The increase in stability of the benzamidine complex relative to the *N,N’*-dimethyl analogue correlates with the solvent H-bond acceptor parameter β_S_, which indicates that the stabilization is due to interactions with the two additional NH H-bond donor sites that are present in the benzamidine complex. There is a substantial increase in the polarity of these two peripheral NH groups when the proton transfer takes place in the benzamidine salt bridge, and the associated increase in free energy contributions due to H-bonding interactions with polar solvents stabilizes the complex. In very polar solvents (THF and DMF), these solvation effects enhance the stability of the benzamidine–formate complex by 2 orders of magnitude.

These conclusions are supported by density functional theory calculations of the H-bond parameters for all of the H-bonding sites in the free and bound species. The H-bond parameters were used to calculate the free energy contributions due to solvent–solute interactions in the primary solvation shell, and the calculations quantitatively predict the experimentally observed solvent effects to within 1 kJ mol^–1^. The approach provides a simple method that accurately predicts the stability of amidinium–carboxylate salt bridges in any solvent. The model also provides a quantitative explanation for the low stability of salt bridges in water if the equilibria between neutral and ionized species in the free state are taken into account. The solvent effects observed here represent an extreme example of what might be expected to be a more general phenomenon: if formation of a complex increases the polarity of peripheral functional groups exposed to the solvent, then more favorable interactions in the solvation shell will give rise to an unexpected stabilization of the complex in polar solvents.^42,43^

While H-bonding interactions are strong in nonpolar solvents, such as chloroform, and solvophobic interactions are strong in polar protic solvents, such as water, both types of interactions are weak in polar aprotic solvents, such as DMF. The exceptionally large association constants reported here in polar aprotic solvents highlight the unique properties of the amidinium–carboxylate salt bridge, making this interaction a very attractive option for the design of supramolecular systems that operate in a largely unexplored area of solvent space.

## Supporting Information Available

Materials and methods, detailed synthetic procedures, characterization including ^1^H and ^13^C NMR spectra of all compounds, ITC titration data, DOSY NMR experiments, and solvent parameters (PDF)
