# Supplementary material for: Solvation-Enhanced Salt Bridges
Source: J Am Chem Soc. 2024 Oct 4;146(41):28580–8. doi: 10.1021/jacs.4c11869 (PMC11487562; doi:10.1021/jacs.4c11869)
Supplement: Supplementary file 1 — ja4c11869_si_001.pdf [file ja4c11869_si_001.pdf]

Solvation-enhanced salt bridges

Ben Iddon and Christopher A. Hunter*

Yusuf Hamied Department of Chemistry, University of Cambridge, Lensfield Road, Cambridge CB2 1EW, UK. E-mail: herchelsmith.orgchem@ch.cam.ac.uk

Supporting Information

Table of Contents

1.	General Experimental Details	S2
2.	Synthesis and Characterisation of Compounds	S3
3.	Isothermal Titration Calorimetry Experiments	S17
4.	Diffusion-Ordered Spectroscopy Experiments	S45
5.	Solvent Parameters	S47
6.	Density Functional Theory Calculations	S48
7.	References	S48

1. General Experimental Details

All the reagents and materials used in the synthesis of the compounds described below were bought from commercial sources, without prior purification. Dry CH_2Cl_2 and THF were obtained from the solvent purification system Pure Solv™ by Innovative Technology. Dry DMF was purchased from Sigma-Aldrich.

Thin layer chromatography was carried out using with silica gel 60F (Merck) on glass plates.

Flash chromatography was carried out on an automated system (Combiflash Rf+ or Combiflash Rf Lumen) using prepacked cartridges of silica (25 µm PuriFlash® columns).

FT-IR spectroscopy was carried out using a PerkinElmer Spectrum One spectrometer equipped with an ATR cell.

HRMS spectroscopy was carried out using Agilent 1100 HPLC coupled with Waters LCT Premier TOF, Waters Xevo G2-S bench top QTOF or Waters Synapt G2-Si Quadrupole-Ton mobility-TOF.

NMR spectroscopy was carried out using a 400 MHz Neo Prodigy Cryoprobe spectrometer, 500 MHz AVIII Smart Probe spectrometer or 700 MHz TXO Cryoprobe spectrometer, using the residual solvent as the internal standard.

In chloroform-*d*, the ^{1}H spectra were referenced to δ 7.26 ppm and ^{13}C spectra were referenced to δ 77.16 ppm. In methanol-d_4, the ^{1}H spectra were referenced to δ 3.31 ppm and ^{13}C spectra were referenced to δ 49.00 ppm. In acetonitrile-d_3, the ^{1}H spectra were referenced to δ 1.94 ppm and ^{13}C spectra were referenced to δ 1.32 ppm.

All chemical shifts (δ) are reported in ppm and coupling constants (*J*) reported in Hz. Splitting patterns are reported as follows: s (singlet), d (doublet), t (triplet), q (quadruplet), hept (heptet), m (multiplet). Broad signals are reported as ‘br’. Peaks are assigned as far as possible to the molecular structure. If the signals for environments a and b overlap, it is assigned ‘a & b’. If the signals for environments a and b are resolved but cannot be distinguished confidently, they are each assigned ‘a / b’.

2. Synthesis and Characterisation of Compounds

Synthesis of Substituted Amidines

Synthesis of Compound **1**

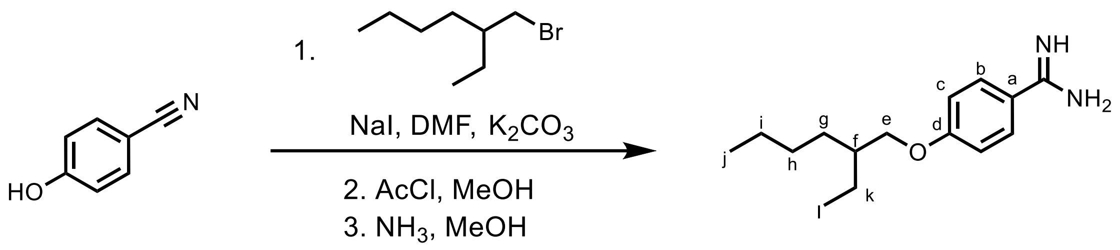

4-Hydroxybenzonitrile (184 mg, 1.54 mmol, 1.0 eq) was dissolved in dry DMF (2 mL) and 2-ethylhexylbromide (407 µL, 2.3 mmol, 1.5 eq), sodium iodide (22mg, 0.15 mmol, 0.1 eq) and potassium carbonate (420 mg, 3.1 mmol, 2.0 eq) were added. The solution was heated at 120 °C in a microwave reactor for 80 minutes then the solvent was removed by flushing under a stream of N_2. The residues were redissolved in EtOAc (10 mL) and washed with water (2 x 10 mL), 5% aqueous LiCl solution (2 x 10 mL) and brine (10 mL) then dried ($MgSO_4$). The crude obtained was used without further purification in the following steps.

The crude was dissolved in dry CH_2Cl_2 (0.5 mL) and MeOH (0.5 mL) and cooled to -78 °C. Acetyl chloride (550 µL, 7.7 mmol, 5.0 eq) was added dropwise and the solution was stirred for 2 hours at room temperature then cooled again to -78 °C. 2.2 mL of 7M methanolic ammonia solution was added dropwise and the solution was stirred at room temperature for 16 hours. The solvent was removed by flushing under a stream of N_2 and the residues were redissolved in CH_2Cl_2 (10 mL) and 1 M aqueous NaOH solution (15 mL) was added. The solution was extracted with CH_2Cl_2 (3 x 10 mL) then dried ($MgSO_4$). The crude was purified by flash column chromatography (SiO_2, 0 - 40% 7M methanolic NH_3 in CH_2Cl_2). The product was obtained as a white solid (205 mg, 0.823 mmol, 53%).

^{1}H NMR (400 MHz, Methanol-*d*$_4$): δ_H 7.73 (d, *J* = 8.8 Hz, 2H, b), 7.04 (d, *J* = 8.8 Hz, 2H, c), 3.96 (d, *J* = 5.6 Hz, 2H, e), 1.74 (hept, *J* = 6.1 Hz, 1H, f), 1.61 – 1.39 (m, 4H, g & k), 1.39 – 1.30 (m, 4H, i & h), 0.95 (t, *J* = 7.5 Hz, 3H, k), 0.92 (t, *J* = 7.0 Hz, 3H, j);

^{13}C NMR (101 MHz, Methanol-*d*$_4$): δ_C 167.6 (N-C=N), 164.3 (d), 130.2 (b), 124.5 (a), 115.7 (c), 71.8 (e), 40.7 (f), 31.6 (g), 30.2 (h), 24.9 (k), 24.1 (i), 14.4 (j), 11.5 (l);

FT-IR (ATR): ν_{max} /cm^{-1} 3314, 2933, 2831, 1636, 1609, 1489, 1464, 1399, 1308, 1262, 1021, 842, 666, 630, 594, 535, 450, 425;

HRMS (ES+): calcd. for $[C_{15}H_{24}N_2O + H]^+$ is 249.1961, found 249.1982 (+ 8.12 ppm).

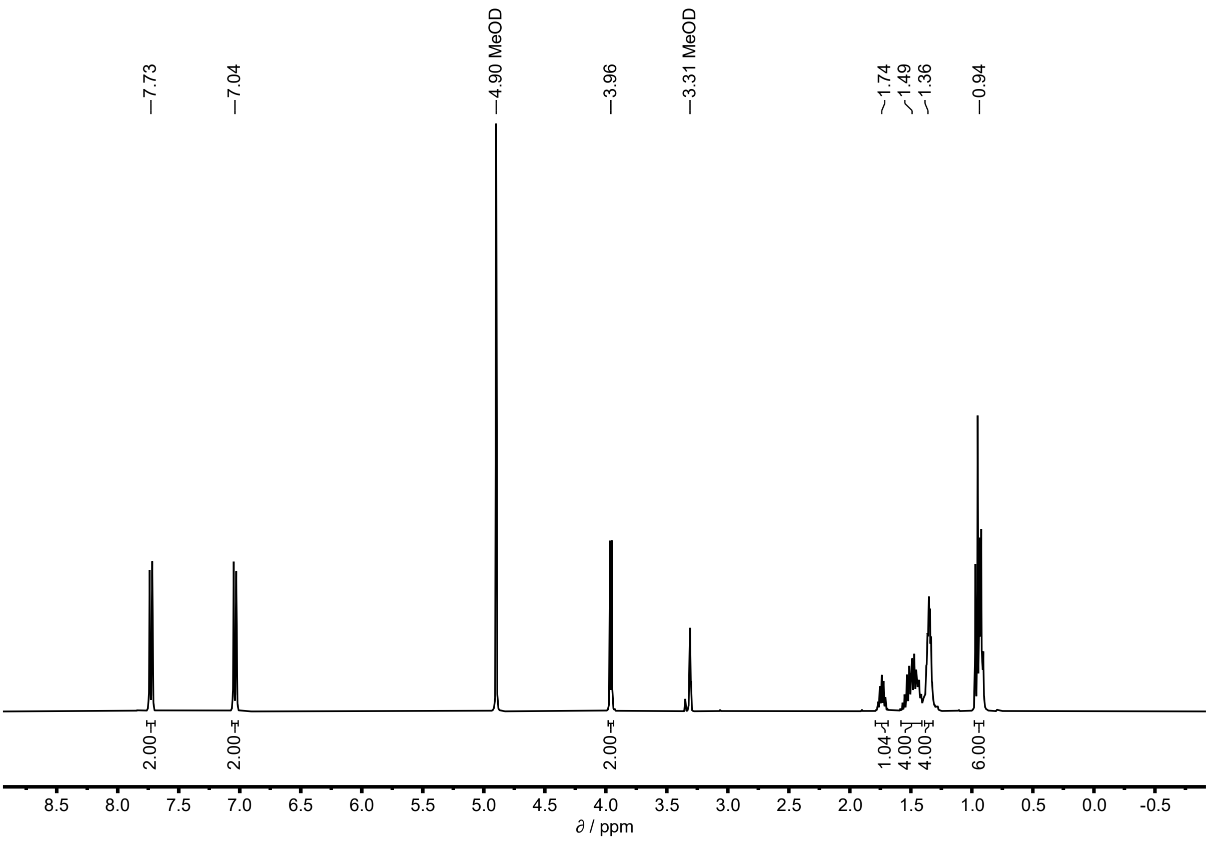

Figure S1: ^{1}H NMR spectrum (400 MHz, CD_3OD) of compound **1**.

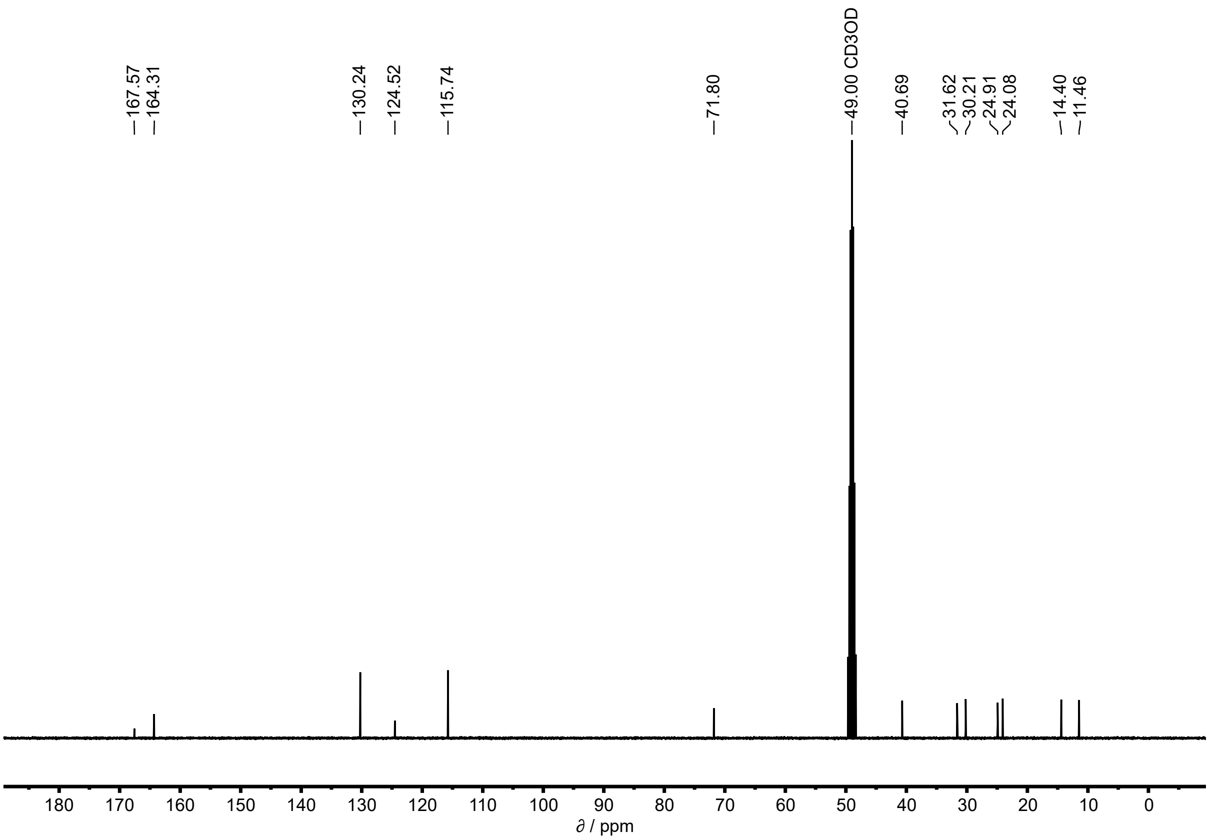

Figure S2: ^{13}C NMR spectrum (101 MHz, CD_3OD) of compound **1**.

Synthesis of Compound **2**

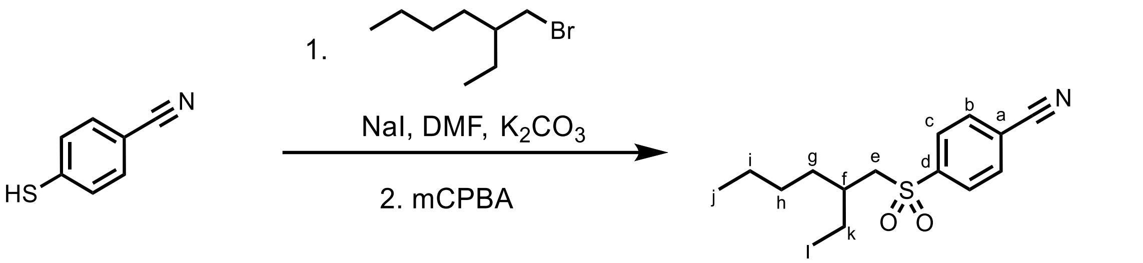

4-Mercaptobenzonitrile (244 mg, 1.8 mmol, 1.0 eq), 2-ethylhexylbromide (640 µL, 3.6 mmol, 2.0 eq), sodium iodide (27 mg, 0.18 mmol 0.1 eq) and potassium carbonate (500 mg, 3.6 mmol, 2.0 eq) were dissolved in dry DMF (5 mL) and the solution was heated at 80 °C for 15 hours. The solvent was removed under a stream of N_2 and the residues dissolved in water (10 mL) and extracted with CH_2Cl_2 (3 x 10 mL). The combined organic phases were washed with 5% aqueous LiCl solution (5 mL) and brine (5 mL) then dried ($MgSO_4$). The crude obtained was used without further purification.

The crude from the previous step was dissolved in dry CH_2Cl_2 (3 mL) and cooled to 5 °C. *m*CPBA (930 mg, 5.4 mmol, 3.0 eq) was added slowly and the solution was stirred at room temperature for 90 minutes. The solution was diluted with sat. $NaHCO_3$ solution (10 mL) and extracted with CH_2Cl_2 (3 x 10 mL). The combined organic phases were dried ($MgSO_4$) and the crude was purified by flash column chromatography (SiO_2, 20% EtOAc in Pet. Ether) to give the product as a colourless oil (399 mg, 1.43 mmol, 79%).

^{1}H NMR (700 MHz, Chloroform-*d*) δ_H 8.04 (d, *J* = 8.0 Hz, 2H, b), 7.87 (d, *J* = 7.9 Hz, 2H, c), 3.03 (dd, *J* = 14.4, 6.2 Hz, 1H, e'), 3.01 (dd, *J* = 14.4, 5.7 Hz, 1H, e''), 1.94 (hept, *J* = 6.2 Hz, 1H, f), 1.52 – 1.41 (m, 2H, k), 1.42 – 1.36 (m, 2H, g), 1.26 – 1.20 (m, 2H, h), 1.19 – 1.13 (m, 2H, i), 0.84 (t, *J* = 7.3 Hz, 1H, j), 0.81 (t, *J* = 7.4 Hz, 1H, l);

^{13}C NMR (176 MHz, Chloroform-*d*): δ_C 144.4 (d), 133.2 (b), 128.8 (c), 117.5 (a / C≡N), 117.2 (a / C≡N), 59.8 (e), 34.5 (f), 32.5 (g), 28.2 (h), 25.8 (k), 22.7 (i), 14.1 (j), 10.23 (k);

FT-IR (ATR): ν_{max} /cm^{-1} 2960, 2930, 2873, 2235, 1760, 1730, 1702, 1462, 1399, 1315, 1303, 1285, 1240, 1184, 1140, 1084, 1018, 841, 803, 752, 639, 573, 652;

HRMS (ES+): calcd. for $[C_{15}H_{21}NO_2S + H]^+$ is 280.1366, found 280.1363 (- 1.13 ppm).

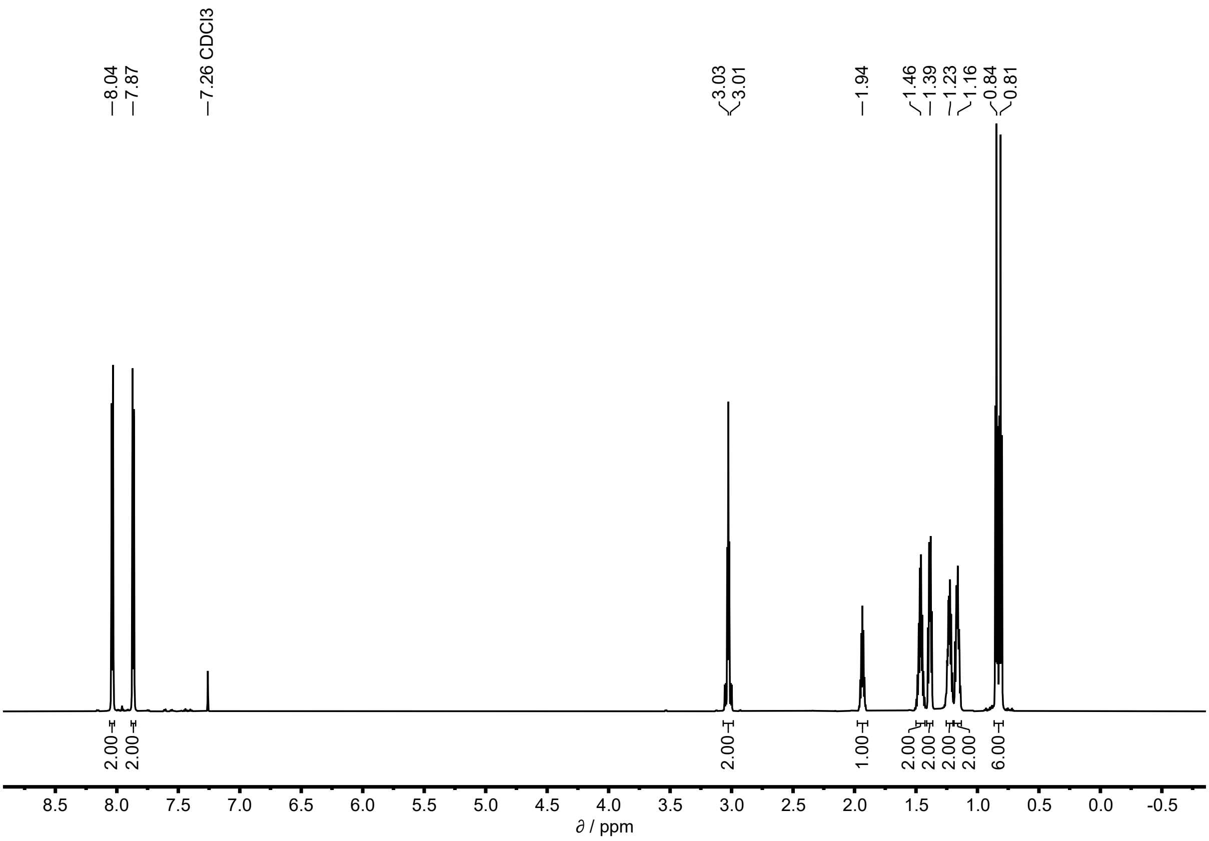

Figure S3: ^{1}H NMR spectrum (700 MHz, $CDCl_3$) of compound **2**.

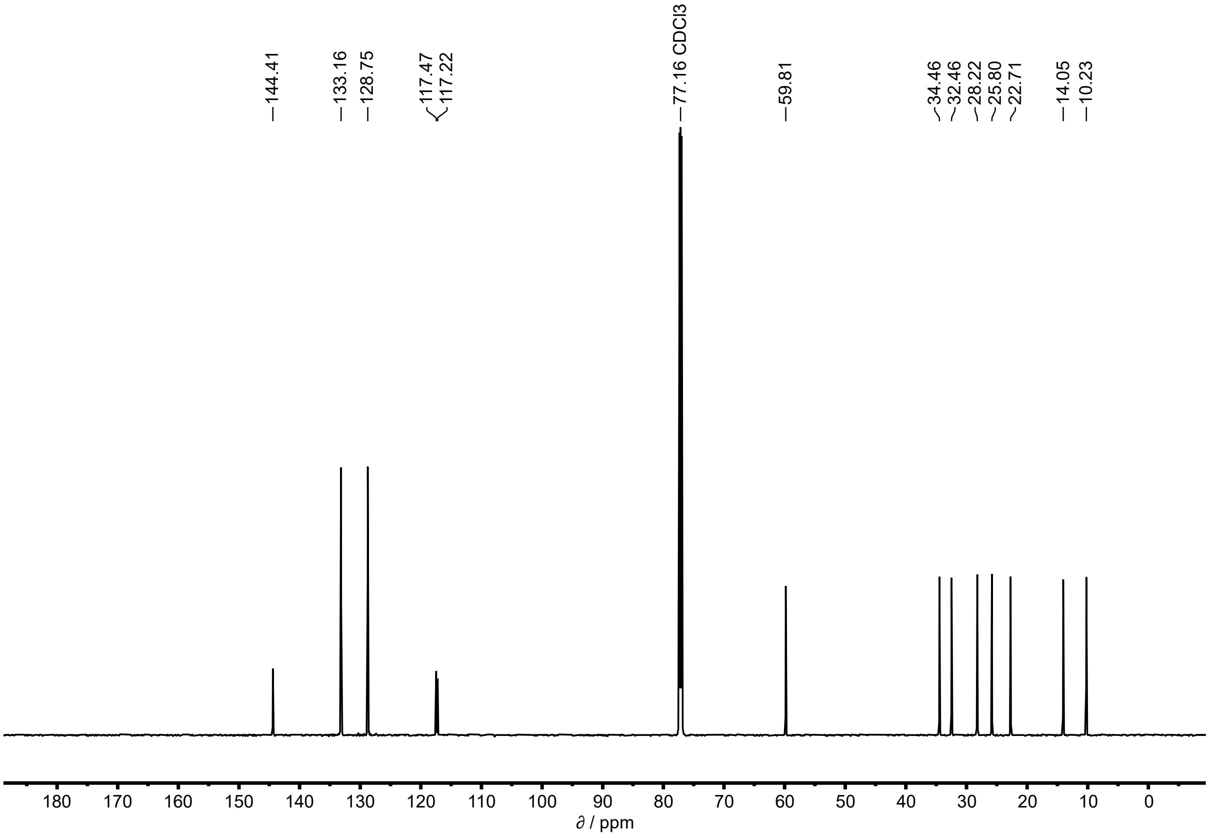

Figure S4: ^{13}C NMR spectrum (176 MHz, $CDCl_3$) of compound **2**.

Synthesis of Compound **3**

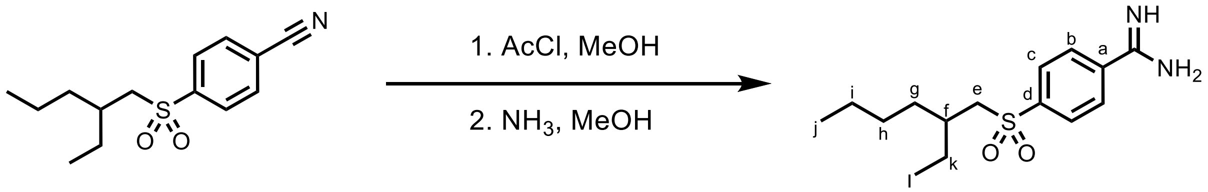

Compound **2** (235 mg, 0.84 mmol, 1.0 eq) was dissolved in dry CH_2Cl_2 (1.6 mL) under an atmosphere of N_2 and MeOH (0.4 mL) was added. The solution was cooled to 0 °C and acetyl chloride (300 µL, 4.2 mmol, 5.0 eq) was added dropwise. The solution was stirred at room temperature for 3 hours then cooled again to 0 °C. 1.2 mL of 7M methanolic ammonia solution was added dropwise and the solution was stirred at room temperature for 16 hours. The solvent was removed by flushing under a stream of N_2 and the residues were redissolved in EtOAc (20 mL) and washed with 1 M aqueous HCl solution (10 mL), 1 M aqueous NaOH solution (2 x 15 mL) then dried ($MgSO_4$). The crude was purified by flash column chromatography (SiO_2, 0 - 15% 7M methanolic NH_3 in CH_2Cl_2). The product was obtained as a colourless oil (8.5 mg, 0.029 mmol, 4%).

^{1}H NMR (400 MHz, Chloroform-*d*): δ_H 7.96 (d, *J* = 8.5 Hz, 2H, c), 7.83 (d, *J* = 8.4 Hz, 2H, b), 4.79 (s, 3H, NH, exchanges with H_2O), 3.03 (d, *J* = 6.0 Hz, 1H, e), 1.95 (hept, *J* = 6.3 Hz, 1H, f), 1.52 – 1.42 (m, 2H, k), 1.42 – 1.36 (m, 2H, g), 1.29 – 1.13 (m, 4H, i & h, overlaps with grease), 0.85 (t, *J* = 7.1 Hz, 3H, j, overlaps with grease), 0.82 (t, *J* = 7.4 Hz, 3H, l, overlaps with grease);

^{13}C NMR (101 MHz, Chloroform-*d*): δ_C 164.1 (N-C=N), 142.5 (d), 140.4 (a), 128.6 (c), 127.5 (b), 60.0 (e), 34.5 (f), 32.5 (g), 28.3 (h), 25.8 (k), 22.8 (i), 14.1 (j), 10.3 (l);

FT-IR (ATR): ν_{max} /cm^{-1} 2959, 2927, 2859, 1642, 1596, 1562, 1459, 1439, 1404, 1311, 1289, 1183, 1147, 1085, 854, 758, 738, 661, 600, 549;

HRMS (ES+): calcd. for $[C_{15}H_{24}N_2O_2S + H]^+$ is 298.1661, found 298.1669 (+ 2.83 ppm).

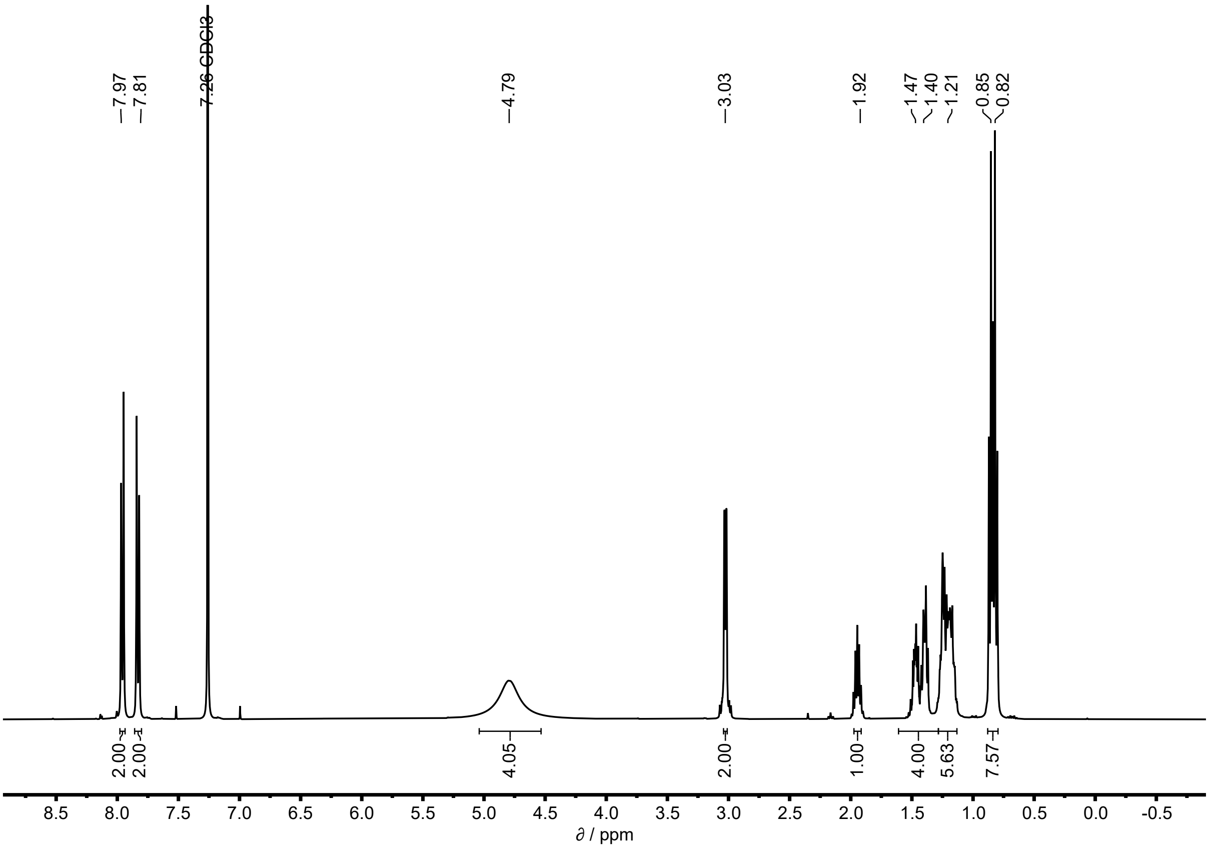

Figure S5: ^{1}H NMR spectrum (400 MHz, $CDCl_3$) of compound **3.**

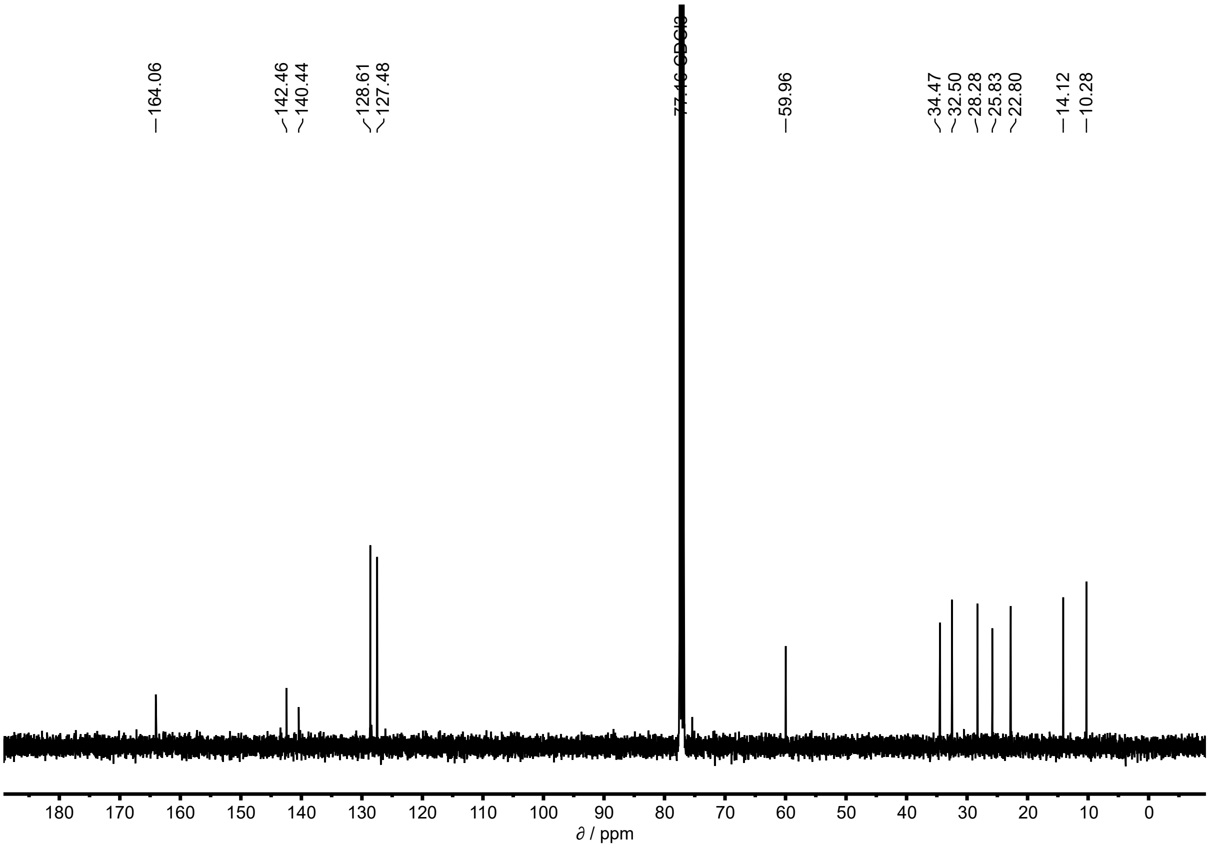

Figure S6: ^{13}C NMR spectrum (101 MHz, $CDCl_3$) of compound **3.**

Synthesis of Compound **4** (*N,N'*-Dimethylbenzamidine)

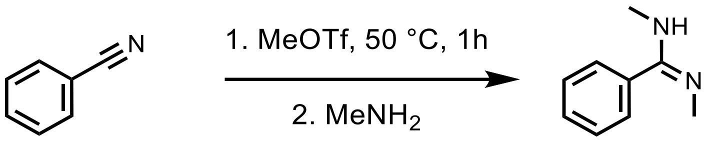

Benzonitrile (200 µL, 1.94 mmol, 1.0 eq) was dissolved in methyl triflate (1 mL) and heated in a microwave reactor at 50 °C for one hour. Excess methyl triflate was removed by flushing under a stream of N_2. The residues were dissolved in methylamine solution (33 wt.% in ethanol, 2 mL) and stirred for 10 minutes. The solvent was removed *in vacuo* and the residues were dissolved in 0.5 M aqueous NaOH solution (10 mL) and extracted with CH_2Cl_2 (3 x 5 mL). The combined organic phases were dried ($MgSO_4$) and the crude was purified by flash column chromatography (SiO_2, 0 - 25% 7M methanolic NH_3 in CH_2Cl_2). The product was obtained as off-white crystals (89.9 mg, 0.61 mmol, 31%).

^{1}H NMR (700 MHz, Acetonitrile-*d₃*): δ_H 7.44 – 7.37 (m, 3H, *meta* and *para*), 7.26 – 7.22 (d, *J* = 6.1 Hz, 2H, *ortho*), 4.63 (s br, 1H, NH), 2.76 (s, 6H, -CH_3);

^{13}C NMR (176 MHz, Acetonitrile-*d₃*): δ_C 161.6 (N-C=N), 136.9 (*ipso*), 129.6 (*para*), 129.3 (*meta*), 128.6 (*ortho*), 33.2 (br, -CH_3);

FT-IR (ATR): ν_{max} /cm^{-1} 3226, 2934, 2866, 1626, 1600, 1516, 1491, 1444, 1404, 1329, 773, 701;

HRMS (ES+): calcd. for $[C_9H_{12}N_2 + H]^+$ is 149.1073, found 149.1077 (+ 2.41 ppm).

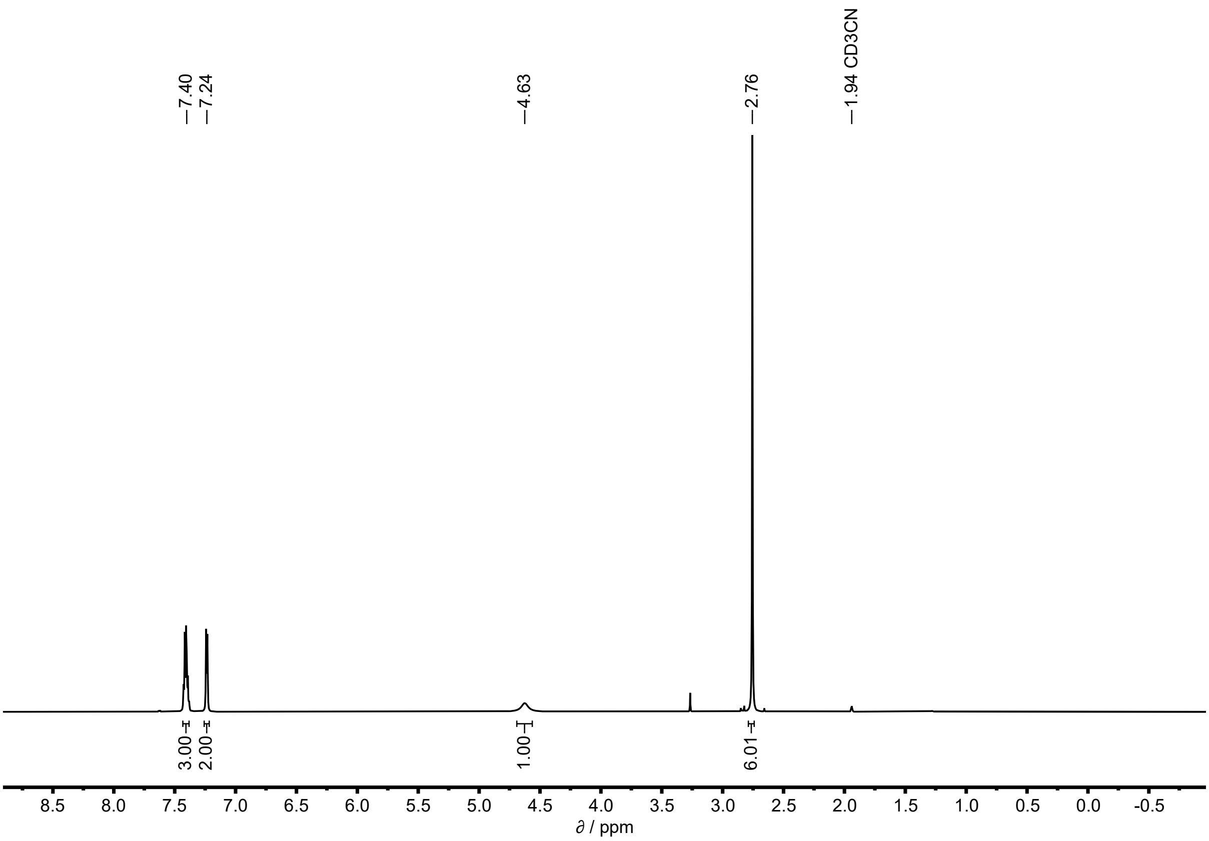

Figure S7: ^{1}H NMR spectrum (700 MHz, CD_3CN) of compound **4.**

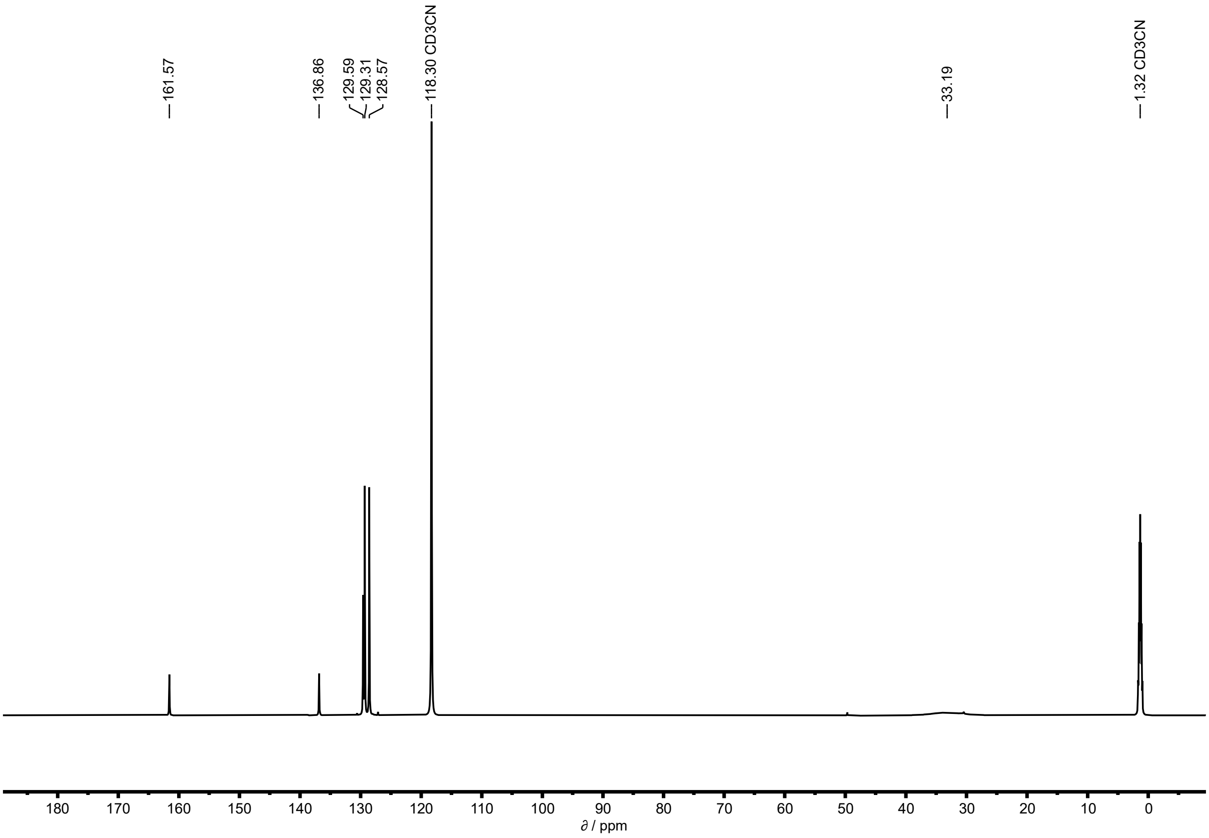

Figure S8: ^{13}C NMR spectrum (176 MHz, CD_3CN) of compound **4.**

Synthesis of Compound **5** (*N,N'*-Diethylbenzamidine)

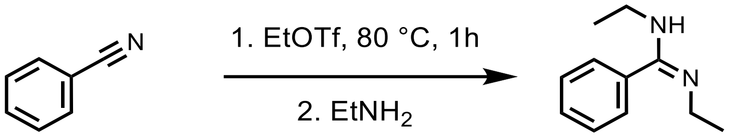

Benzonitrile (200 µL, 1.94 mmol, 1.0 eq) was dissolved in ethyl triflate (2 mL) and heated at 80 °C for one hour. Excess ethyl triflate was removed by flushing under a stream of N_2. The residues were dissolved in dry THF (0.5 mL) and cooled to – 78°C then ethylamine solution (2M in THF, 2 mL) was added and the solution was stirred for 2 hours. The solvent was removed *in vacuo* and the residues were dissolved in 1 M aqueous NaOH solution (10 mL) and extracted with CH_2Cl_2 (3 x 5 mL). The combined organic phases were dried ($MgSO_4$) and the crude was purified by flash column chromatography (SiO_2, 0 - 25% 7M methanolic NH_3 in CH_2Cl_2). The product was obtained as off-white crystals (186.5 mg, 1.05 mmol, 54%).

^{1}H NMR (700 MHz, Acetonitrile-*d*$_3$): δ_H 7.43 – 7.36 (m, 3H, *meta* and *para*), 7.24 (d, *J* = 6.2 Hz, 2H, *ortho*), 4.44 (s br, 1H, NH), 3.12 (s br, 4H, $-CH_2-$), 1.07 (t, *J* = 7.3 Hz, 6H, $-CH_3$);

^{13}C NMR (176 MHz, Acetonitrile-*d*$_3$): δ_C 159.4 (N-C=N), 137.2 (*ipso*), 129.4 (*para*), 129.2 (*meta*), 128.5 (*ortho*), 40.9 (br, $-CH_2-$), 16.6 (br, $-CH_3$);

FT-IR (ATR): ν_{max} /cm^{-1} 3207, 2963, 2928, 2902, 2885, 2862, 2847, 1614, 1597, 1534, 1444, 1371, 1318, 1305, 774, 700;

HRMS (ES+): calcd. for $[C_{11}H_{16}N_2 + H]^+$ is 177.1386, found 177.1390 (+ 1.87 ppm).

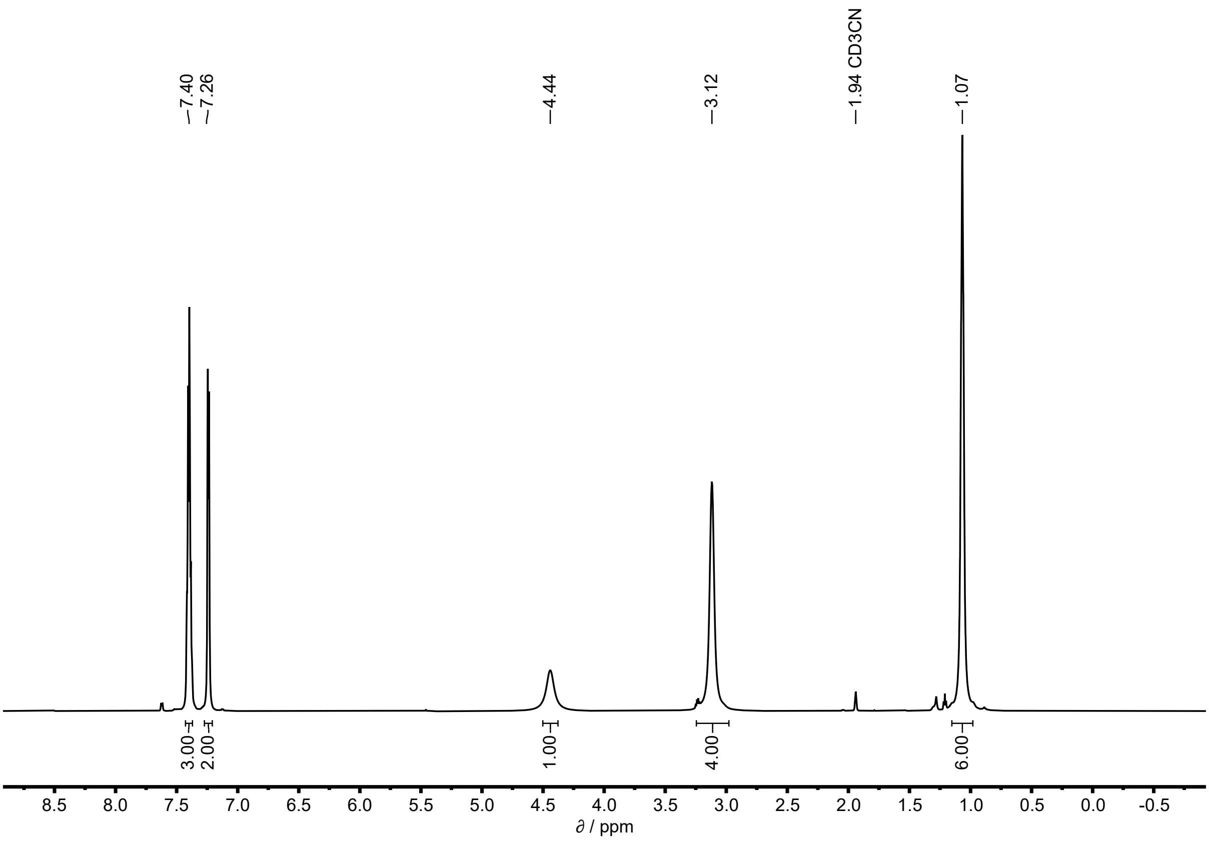

Figure S9: ^{1}H NMR spectrum (700 MHz, CD_3CN) of compound **5**.

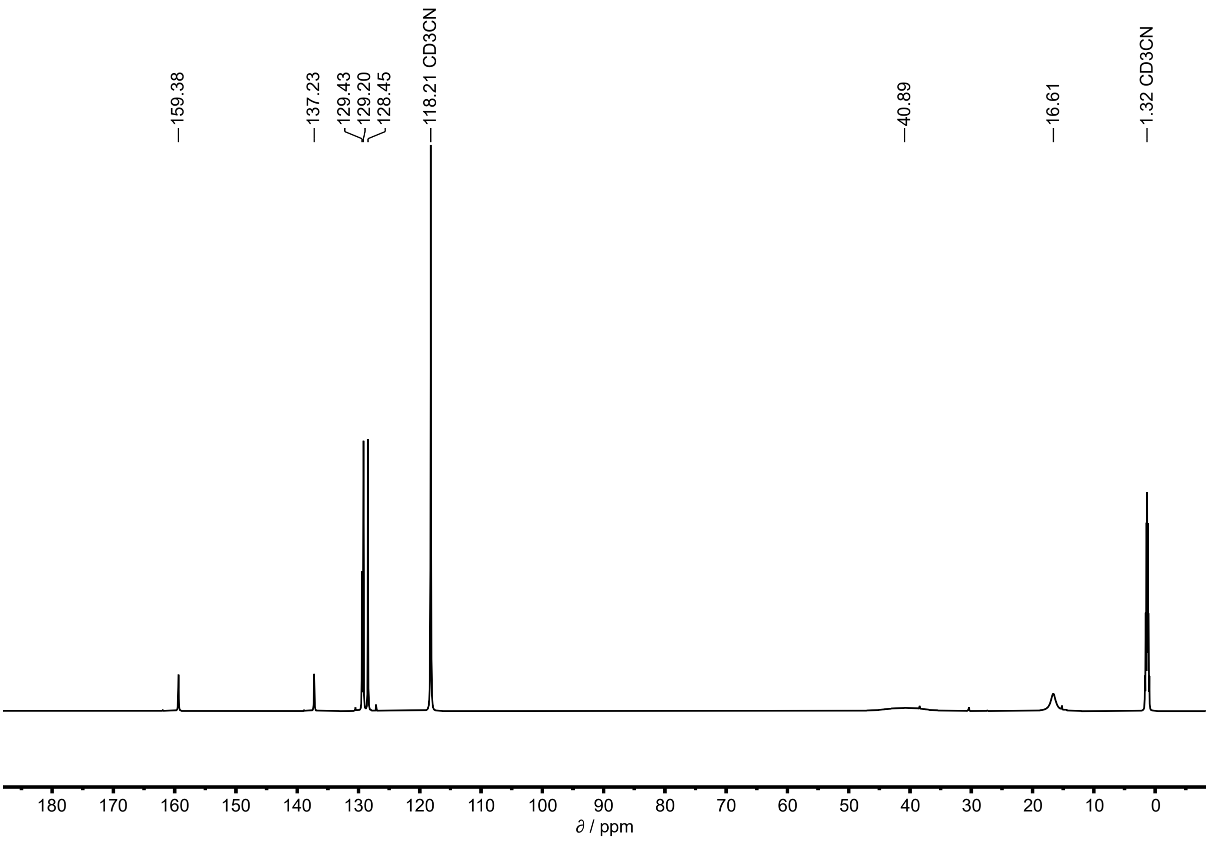

Figure S10: ^{13}C NMR spectrum (176 MHz, CD_3CN) of compound **5**.

Synthesis of Compound **6** (*N,N'*-Di-*iso*-propylbenzamidine)

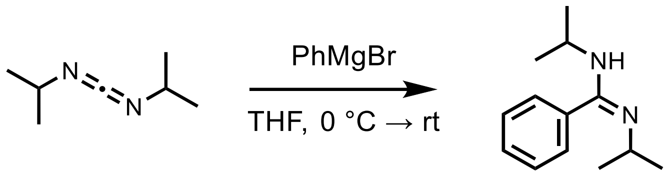

N,N'-Di-*iso*-propylcarbodiimide (77 µL, 0.5 mmol, 1.0 eq) was dissolved in dry THF (2 mL) under an atmosphere of N_2 and cooled to 0 °C. Phenyl magnesium bromide (550 µL of a 1M solution in THF, 0.55 mmol, 1.1 eq) was added dropwise and the solution was warmed to room temperature and stirred for 2 hours. The reaction was quenched by addition of water (1 mL) then the THF was removed under a stream of nitrogen. 10% Aqueous NaOH solution was added (1 mL) and the solution was extracted with CH_2Cl_2 (3 x 5 mL). The combined organic phases were dried ($MgSO_4$). The crude was purified by flash column chromatography (SiO_2, 0 - 15% 7M methanolic NH_3 in CH_2Cl_2) to give the product as a yellow oil (54 mg, 0.26 mmol, 53%).

^{1}H NMR (400 MHz, Chloroform-*d*): δ_H 7.44 – 7.34 (m, 3H, *meta* and *para*), 7.25 (m, 2H, *ortho*, overlaps with $CDCl_3$), 3.52 (s br, 2H, CH), 1.09 (d, *J* = 6.4 Hz, 12H, $-CH_3$);

^{13}C NMR (101 MHz, Chloroform-*d*): δ_C 157.5 (N-C=N), 135.8 (*ipso*), 128.9 (*para*), 128.6 (*meta*), 127.4 (*ortho*), 46.2 (br, CH), 24.1 ($-CH_3$);

FT-IR (ATR): ν_{max} /cm^{-1} 2961, 2930, 2869, 1632, 1600, 1484, 1467, 1446, 1360, 1319, 1274, 1174, 1128, 767, 702;

HRMS (ES+): calcd. for $[C_{13}H_{20}N_2 + H]^+$ is 205.1699, found 205.1700 (+ 0.43 ppm).

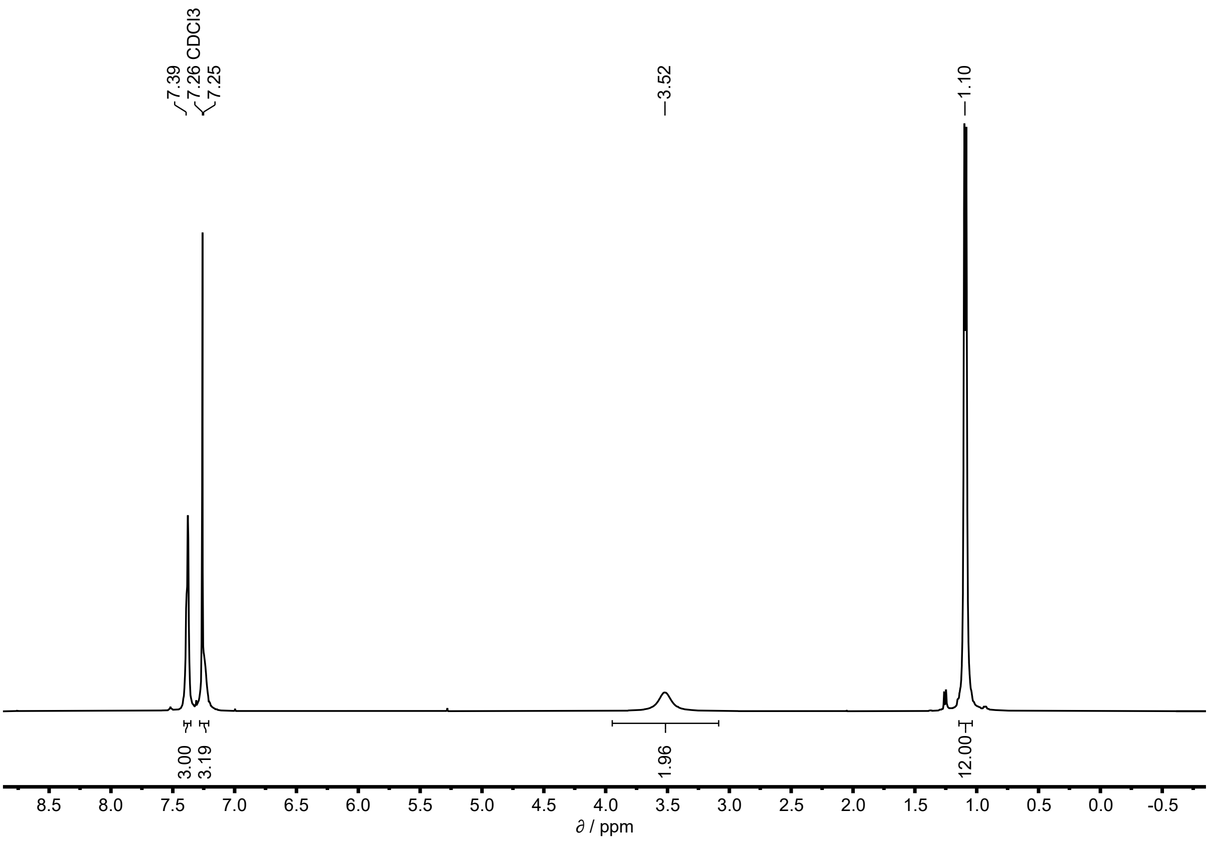

Figure S11: ^{1}H NMR spectrum (400 MHz, $CDCl_3$) of compound **6**.

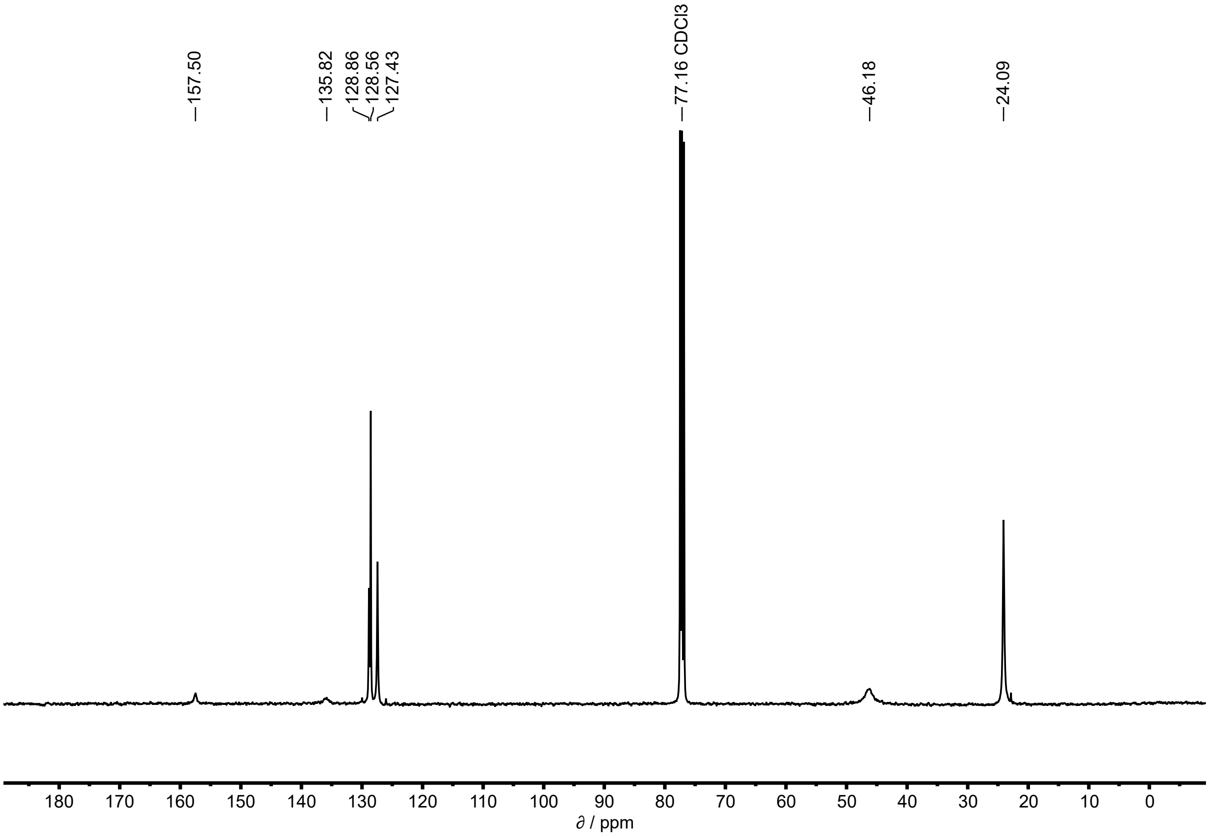

Figure S12: ^{13}C NMR spectrum (101 MHz, $CDCl_3$) of compound **6**.

Synthesis of Compound **7** (*N,N'*-Di-*tert*-butylbenzamidine)

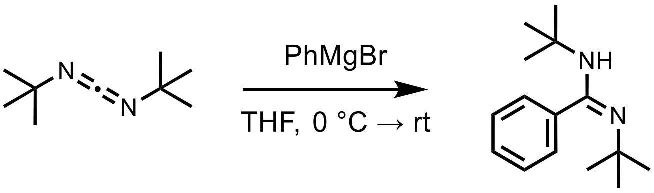

N,N'-Di-*tert*-butylcarbodiimide (239 mg, 1.55 mmol, 1.0 eq) was dissolved in dry THF (2 mL) under an atmosphere of N_2 and cooled to 0 °C. Phenyl magnesium bromide (1.86 mL of a 1M solution in THF, 1.86 mmol, 1.2 eq) was added dropwise and the solution was warmed to room temperature and stirred for 16 hours. The reaction was quenched by addition of water (1 mL) then the THF was removed under a stream of nitrogen. 10% Aqueous NaOH solution was added (5 mL) and the solution was extracted with CH_2Cl_2 (3 x 5 mL). The combined organic phases were dried ($MgSO_4$). The crude was purified by flash column chromatography (SiO_2, 0 - 15% 7M methanolic NH_3 in CH_2Cl_2) to give the product as off white crystals (159 mg, 0.685 mmol, 44%).

^{1}H NMR (500 MHz, Chloroform-*d*): δ_H 7.33 – 7.28 (m, 3H, *meta* and *para*), 7.27 – 7.22 (m, 2H, *ortho*, overlaps with $CDCl_3$), 1.20 (s br, 18H, $-CH_3$);

^{13}C NMR (126 MHz, Chloroform-*d*): δ_C 152.8 (N-C=N), 140.8 (*ipso*), 128.3 (*para*), 127.9 (*meta*), 127.8 (*ortho*), 52.2 (^{t}Bu, C), 31.4 (^{t}Bu, $-CH_3$);

FT-IR (ATR): ν_{max} /cm^{-1} 2960, 2927, 2903, 1644, 1600, 1484, 1444, 1384, 1356, 1288, 1203, 1140, 969, 789, 744, 701;

HRMS (ES+): calcd. for $[C_{15}H_{24}N_2 + H]^+$ is 233.2012, found 233.2023 (+ 4.71 ppm).

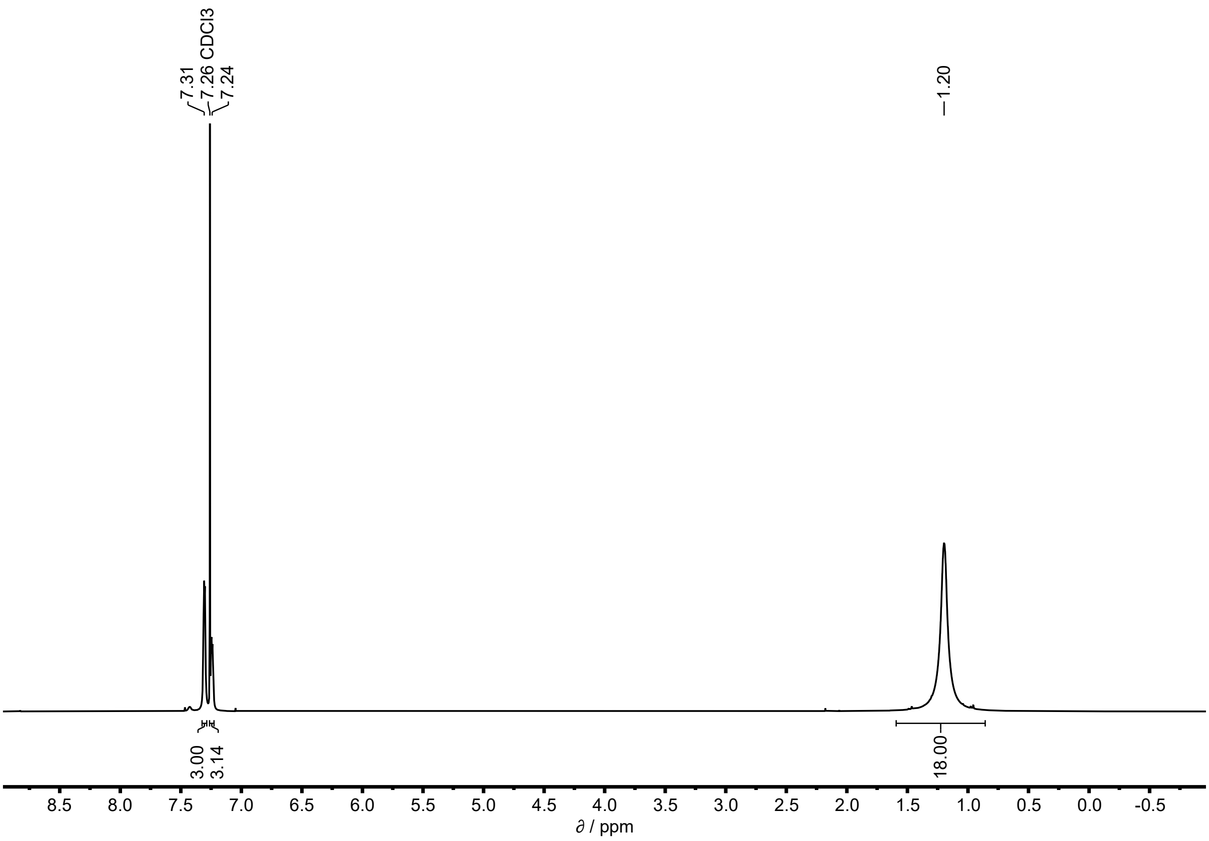

Figure S13: ^{1}H NMR spectrum (500 MHz, $CDCl_3$) of compound **7**.

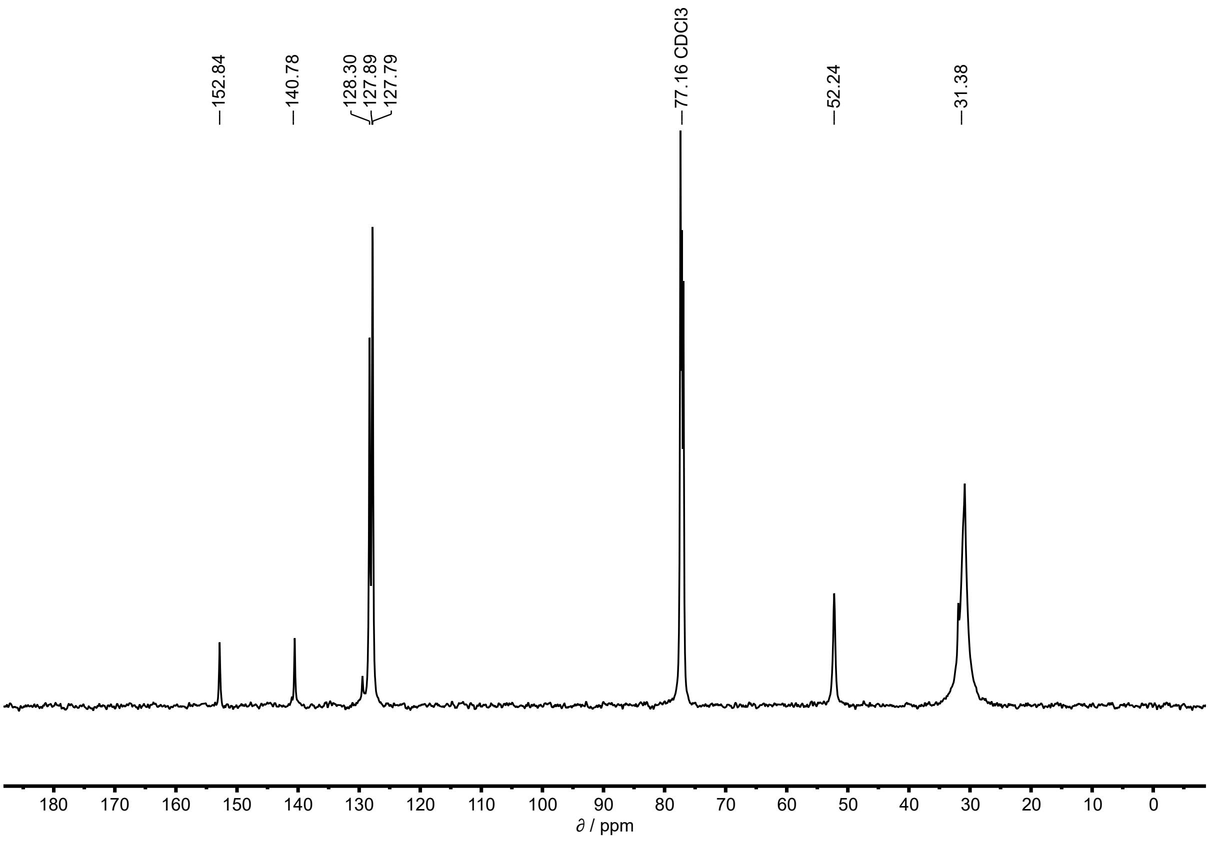

Figure S14: ^{13}C NMR spectrum (126 MHz, $CDCl_3$) of compound **7**.

3. Isothermal Titration Calorimetry Experiments

Isothermal Titration Calorimetry (ITC) experiments were carried out at 298 K using a Malvern MicroCal VP-ITC MicroCalorimeter.

HPLC-plus grade chloroform stabilised with amylenes was filtered over basic alumina and magnesium sulfate before use. Anisole was dried over activated molecular sieves before use. Acetonitrile was taken from the solvent purification system Pure Solv™. Acetone, ethyl acetate and DMF were purchased extra dry with AcroSeal™ over molecular sieves. Extra pure DME stabilised with BHT was used. HPLC grade THF was distilled before use.

In general, a host solution of concentration 50 – 100 times the expected dissociation constant was prepared and loaded into the sample cell of the microcalorimeter. A guest solution of concentration of approximately 10 times the host concentration was prepared and loaded into the injection syringe. The volume of injections was between 1 – 24 µL and the total volume of guest solution injected was 280 µL. The thermogram peaks were integrated and the first injection was discarded. Thermodynamic parameters were calculated using the MicroCal PEAQ-ITC Analysis Software which uses least-squares minimisation to obtain globally minimised parameters, allowing the parameter N to vary. In all cases the data fitted well to a 1:1 binding isotherm.

The ITC titrations of formic acid into *N,N'*-dimethylbenzamidine in DME, THF and DMF were carried out in the low c-value regime. In these cases the host solution was prepared with concentration equal to the expected dissociation constant and the guest solution was prepared at a concentration to achieve at least 70% saturation of host at the end of the titration. These titrations were fitted with the parameter N fixed at 1.00.

Benzamidine with Benzoic Acid

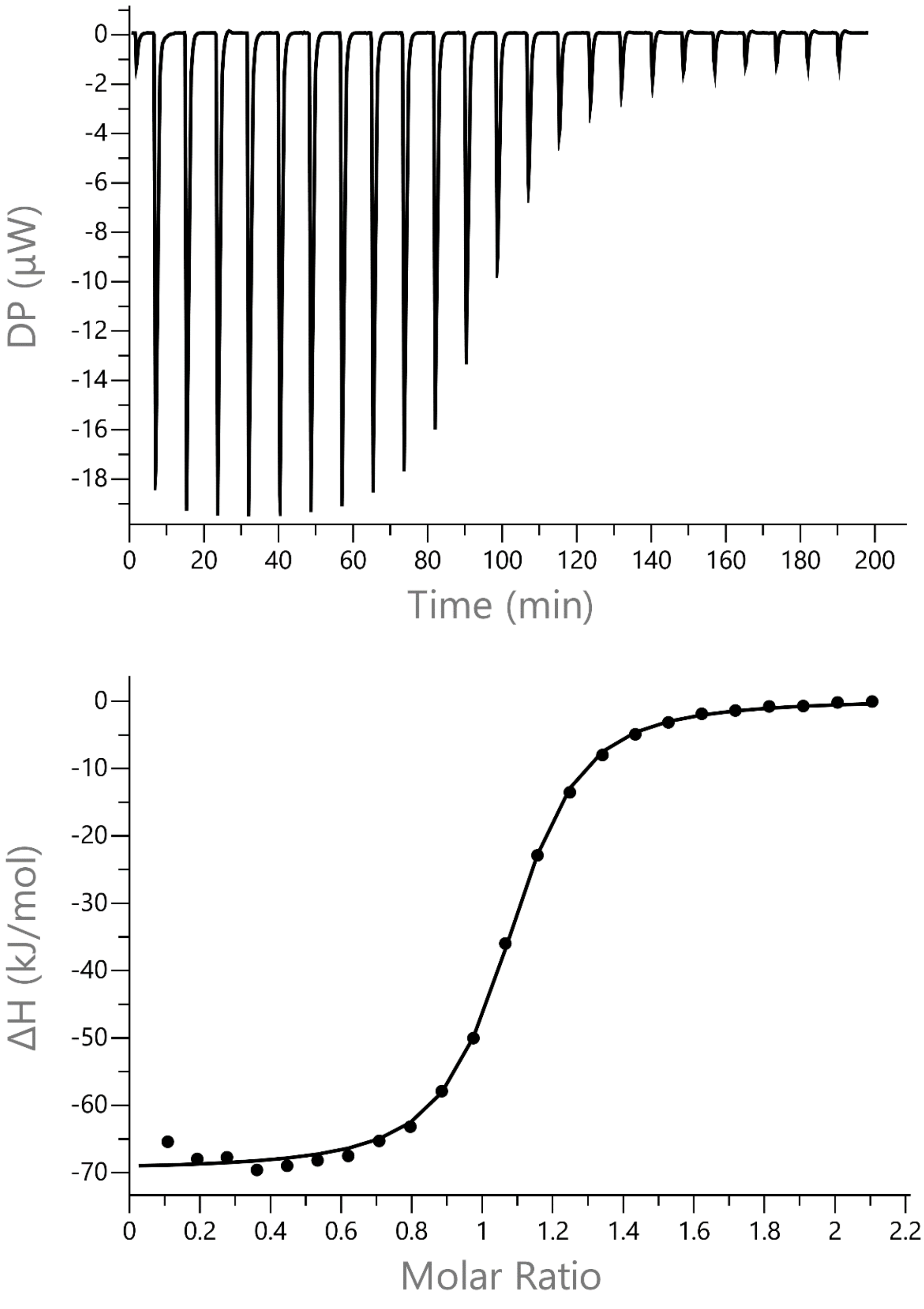

Figure S15: ITC data for titration of benzamidine (0.99 mM) into benzoic acid (0.1 mM) in chloroform at 298 K. The raw data for each injection is shown (differential power, DP), along with the least-squares-fit of the enthalpy change per mole of guest (*ΔH*) to a 1:1 binding isotherm.

Variation of Benzoic Acid Substituent X

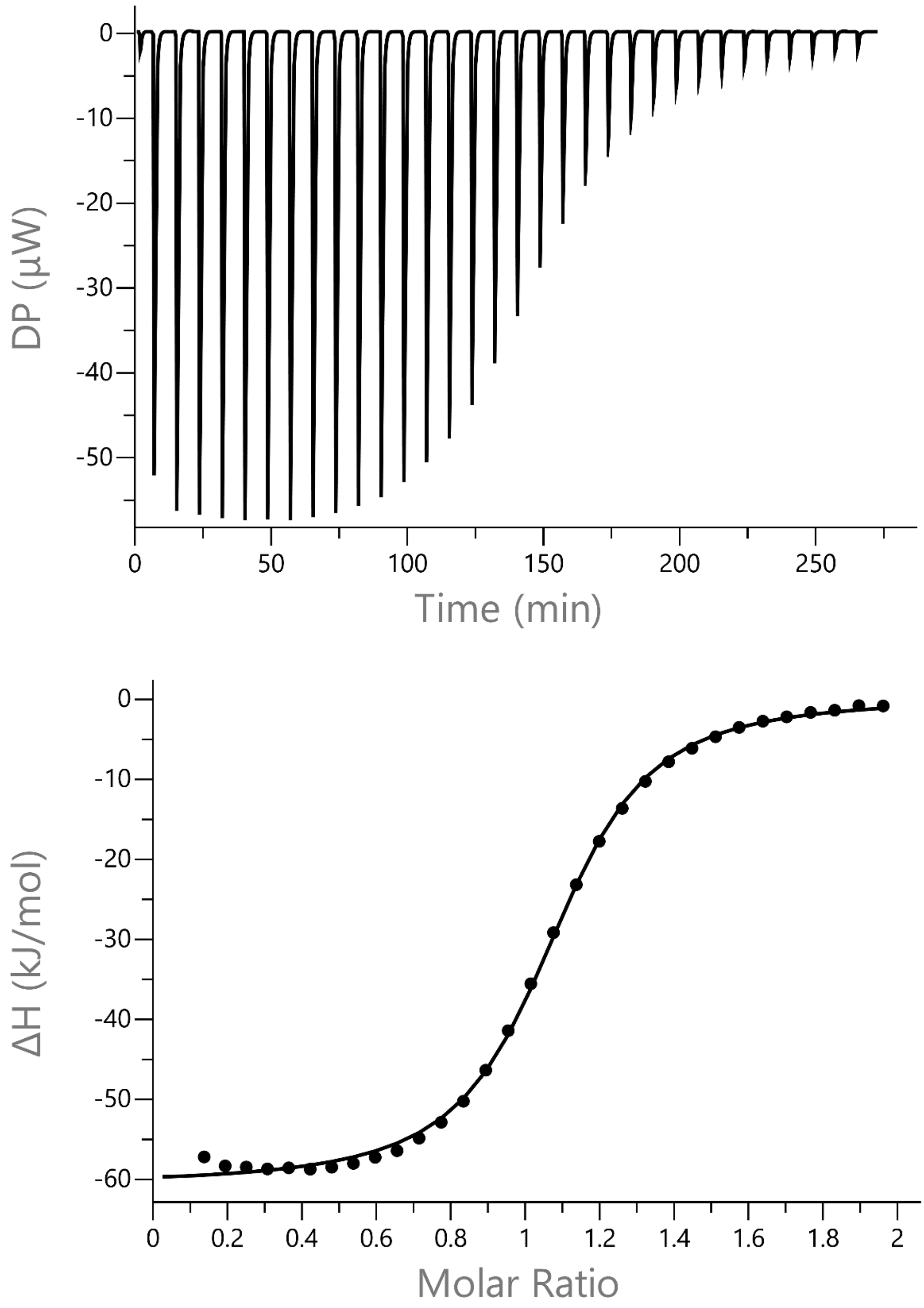

Figure S16: ITC data for titration of benzamidine (4.3 mM) into *p*-dimethylaminobenzoic acid (0.43 mM) in chloroform at 298 K. The raw data for each injection is shown (differential power, DP), along with the least-squares-fit of the enthalpy change per mole of guest (ΔH) to a 1:1 binding isotherm.

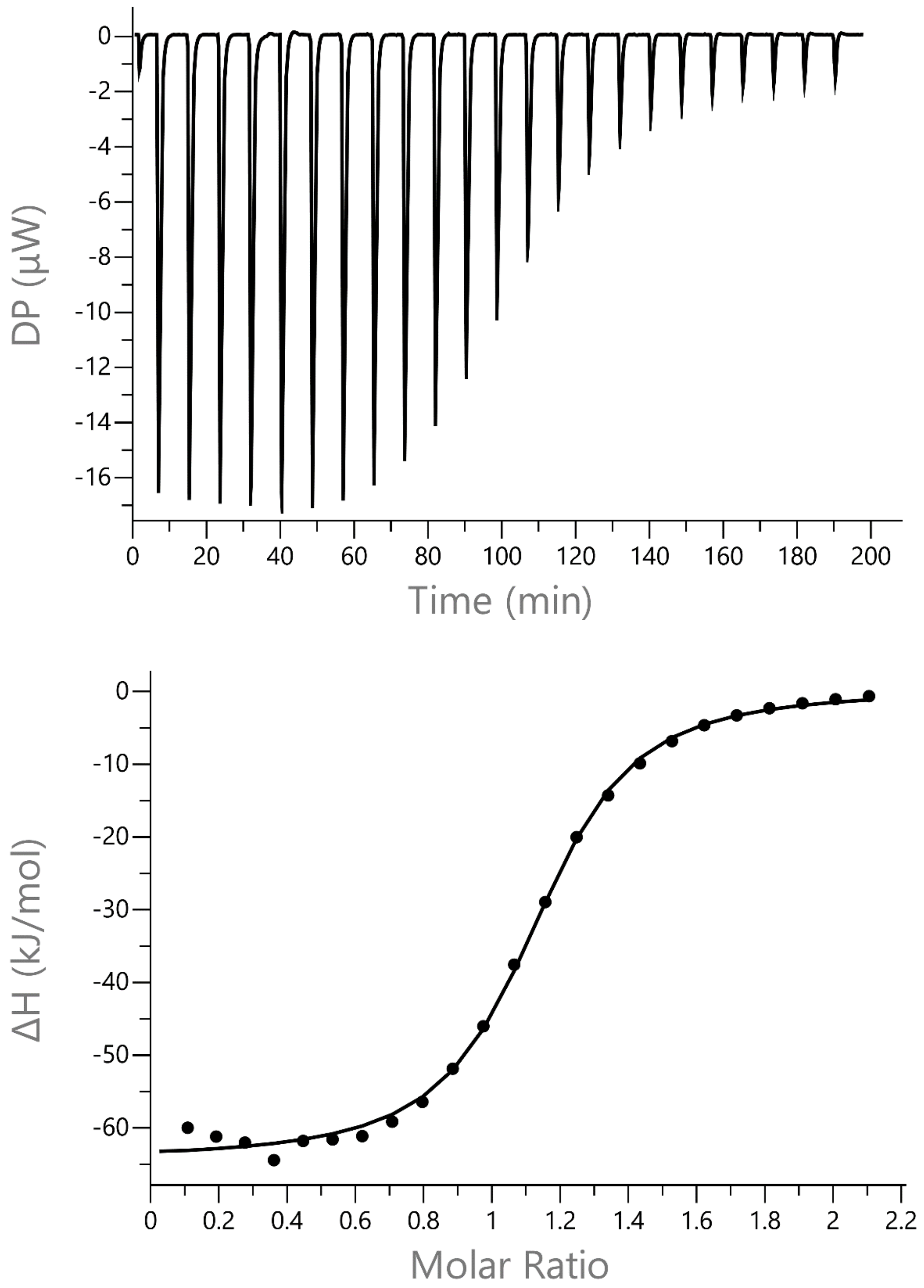

Figure S17: ITC data for titration of benzamidine (0.99 mM) into *p*-methoxybenzoic acid (0.1 mM) in chloroform at 298 K. The raw data for each injection is shown (differential power, DP), along with the least-squares-fit of the enthalpy change per mole of guest (*ΔH*) to a 1:1 binding isotherm.

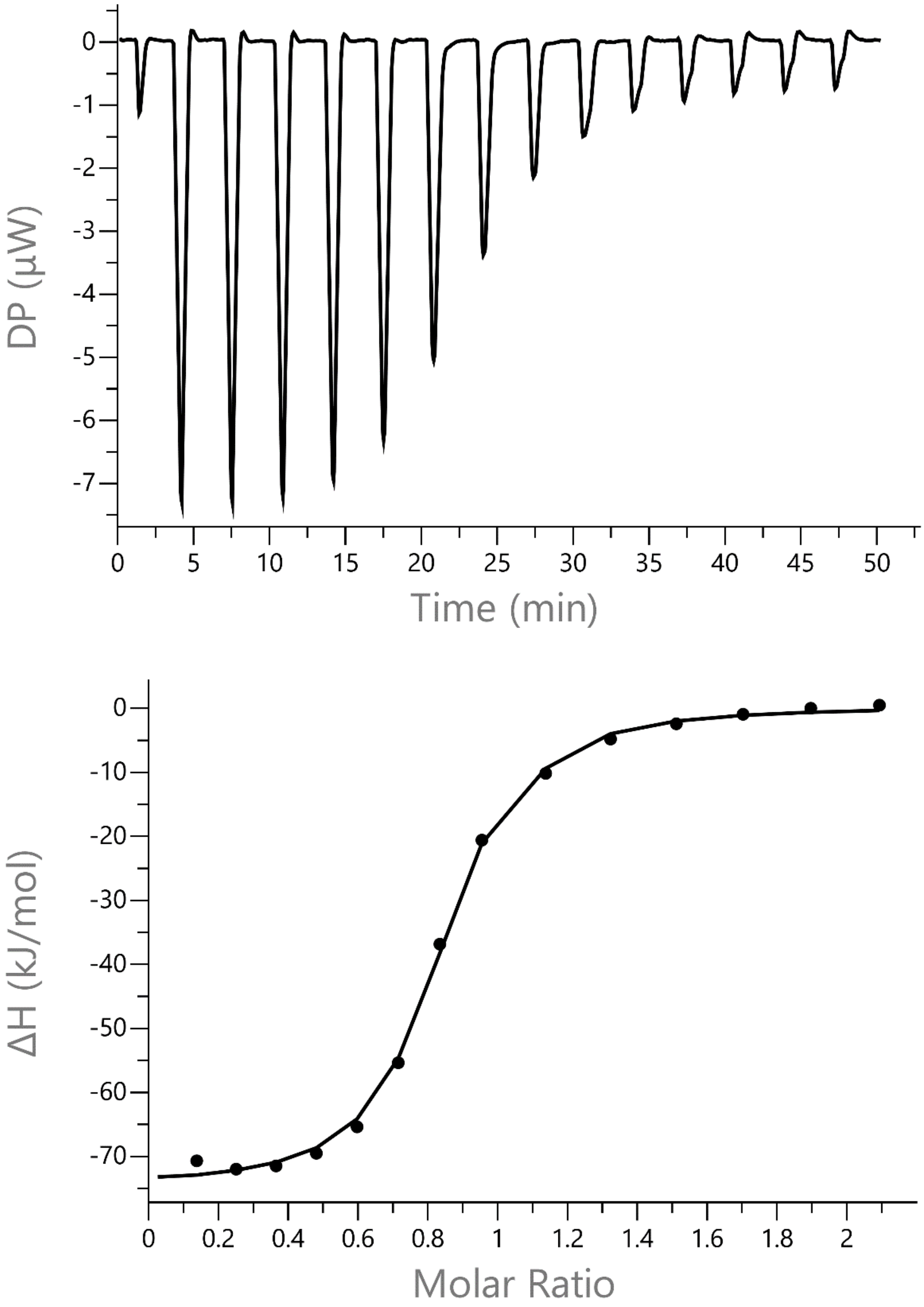

Figure S18: ITC data for titration of benzamidine (0.2 mM) into *p*-trifluoromethylbenzoic acid (0.02 mM) in chloroform at 298 K. The raw data for each injection is shown (differential power, DP), along with the least-squares-fit of the enthalpy change per mole of guest (*ΔH*) to a 1:1 binding isotherm.

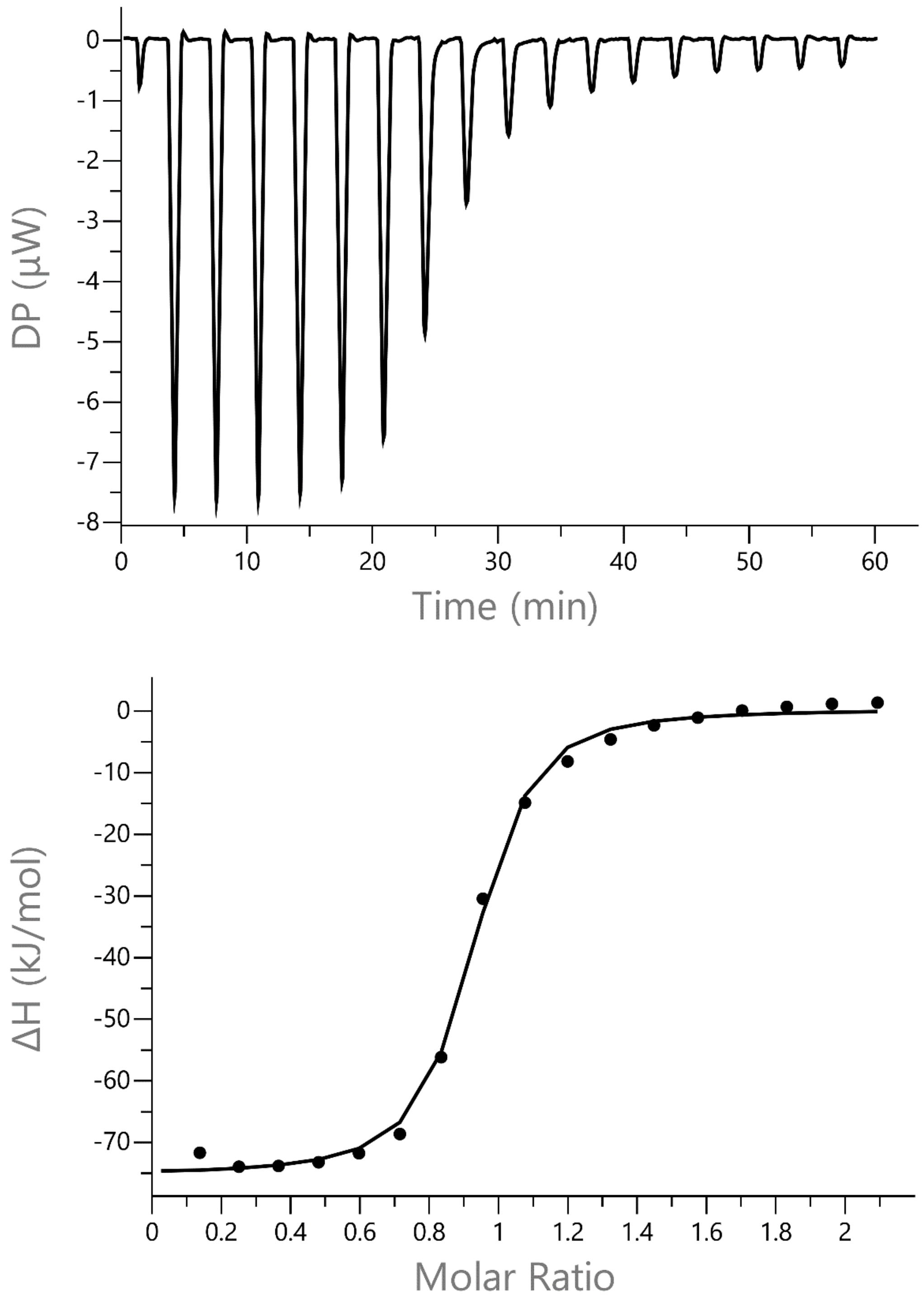

Figure S19: ITC data for titration of benzamidine (0.2 mM) into *p*-nitrobenzoic acid (0.02 mM) in chloroform at 298 K. The raw data for each injection is shown (differential power, DP), along with the least-squares-fit of the enthalpy change per mole of guest (*ΔH*) to a 1:1 binding isotherm.

Variation of Aromatic Benzamidine Substituent Y

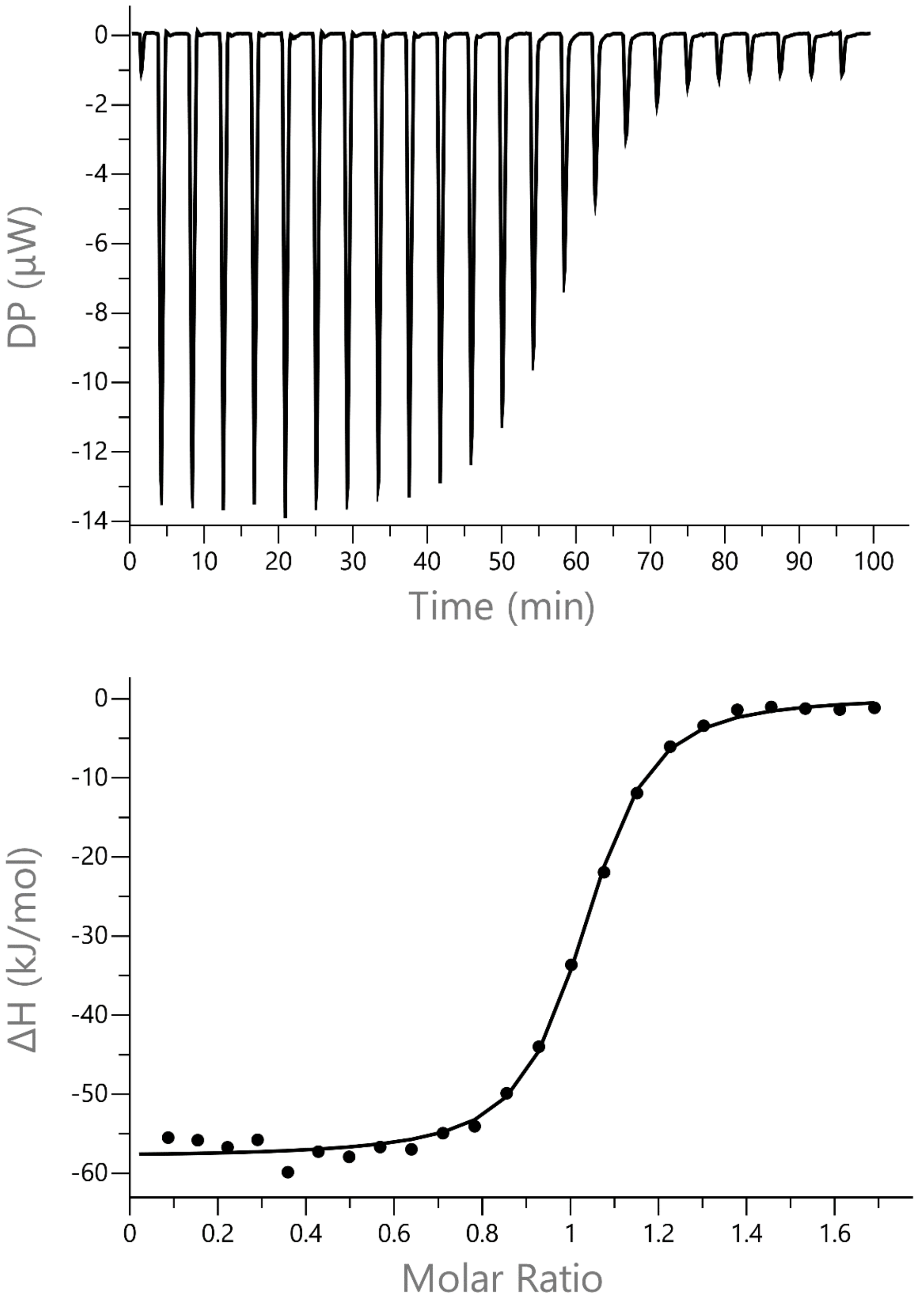

Figure S20: ITC data for titration of compound **1** (0.5 mM) into benzoic acid (0.05 mM) in chloroform at 298 K. The raw data for each injection is shown (differential power, DP), along with the least-squares-fit of the enthalpy change per mole of guest (*ΔH*) to a 1:1 binding isotherm.

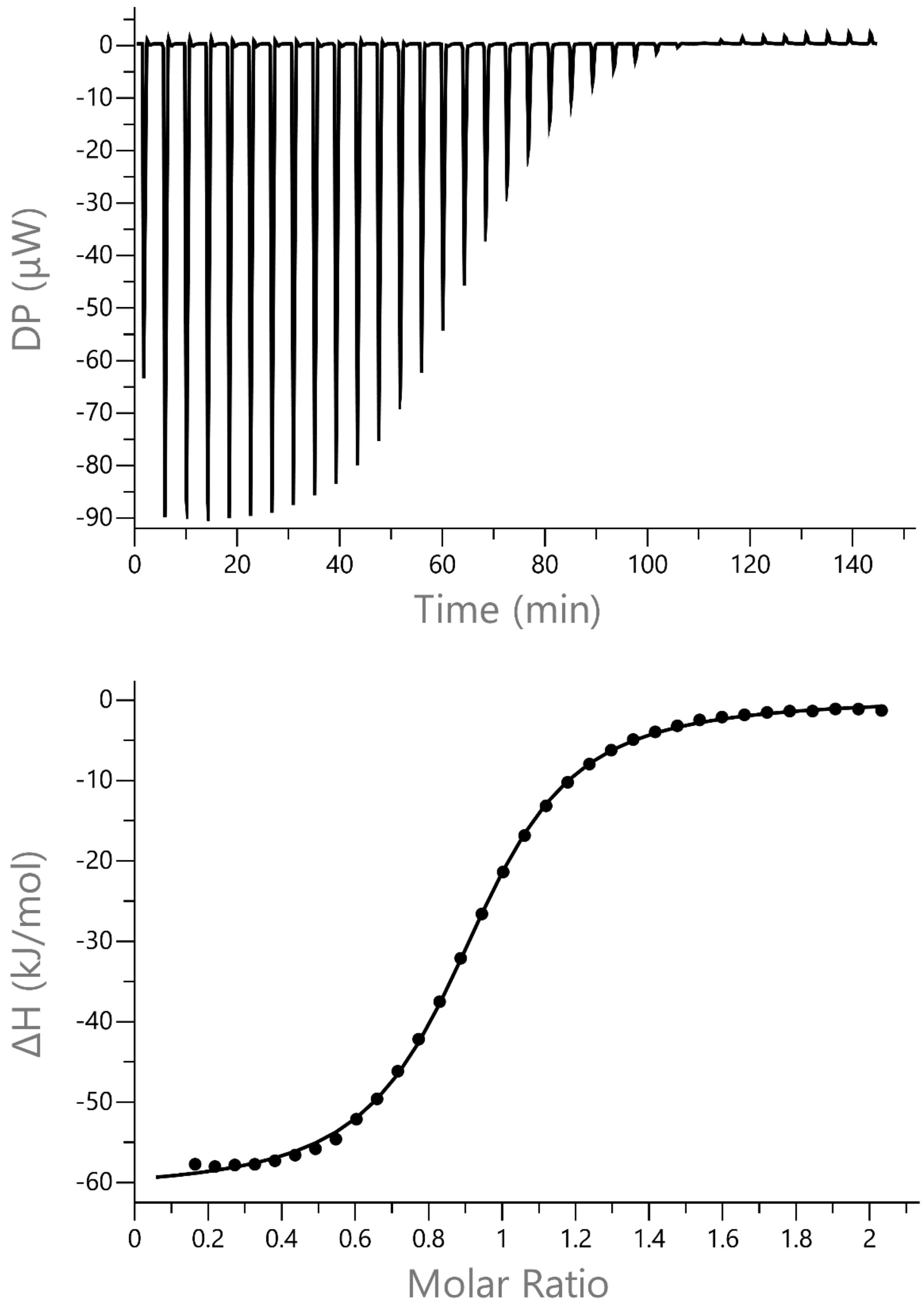

Figure S21: ITC data for titration of benzoic acid (4.7 mM) into compound **3** (0.5 mM) in chloroform at 298 K. The raw data for each injection is shown (differential power, DP), along with the least-squares-fit of the enthalpy change per mole of guest (*ΔH*) to a 1:1 binding isotherm.

Variation of Benzamidine *N*-Alkyl Substituent R

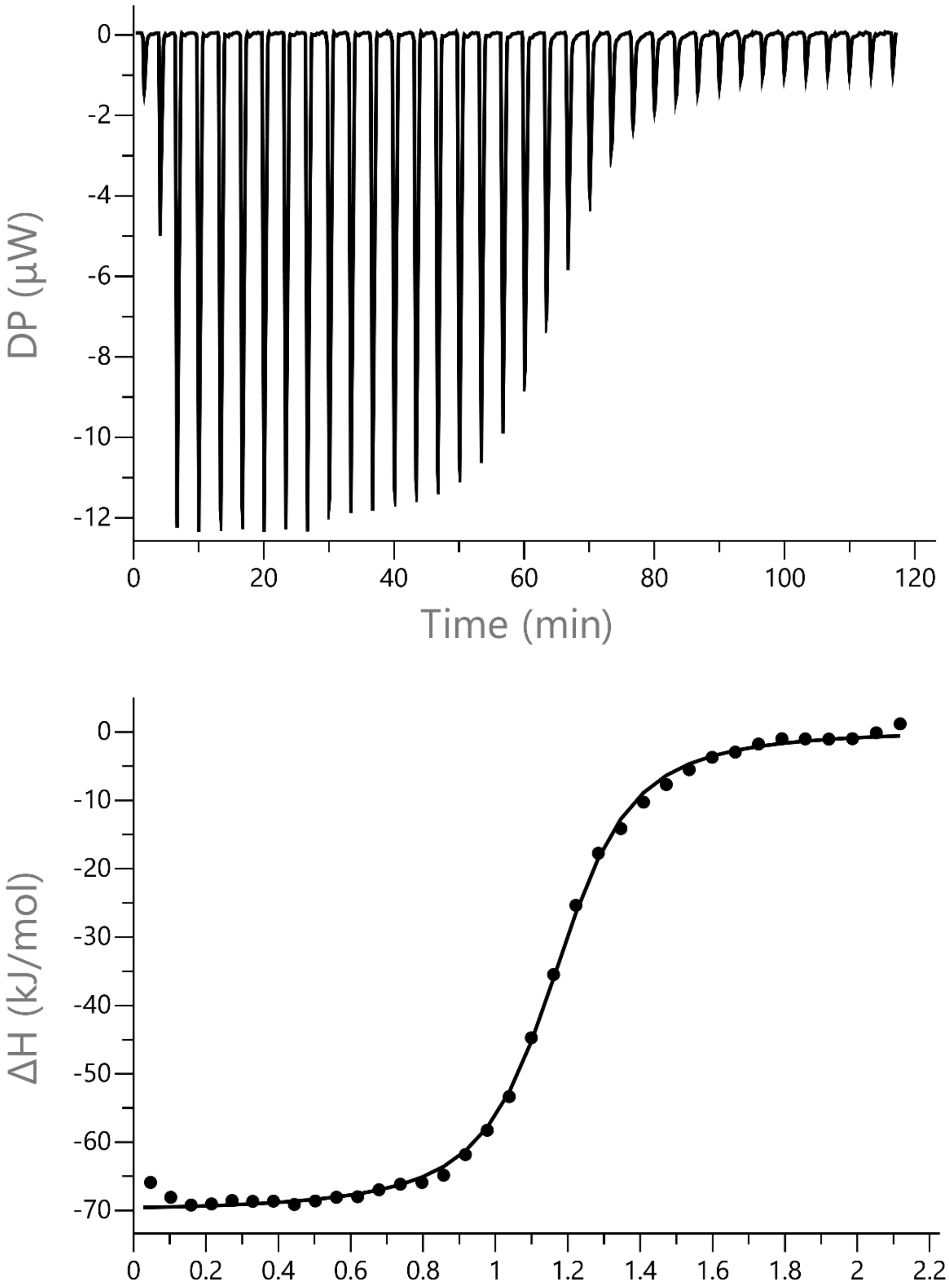

Figure S22: ITC data for titration of benzoic acid (0.5 mM) into *N,N'*-dimethylbenzamidine (0.05 mM) in chloroform at 298 K. The raw data for each injection is shown (differential power, DP), along with the least-squares-fit of the enthalpy change per mole of guest (*ΔH*) to a 1:1 binding isotherm.

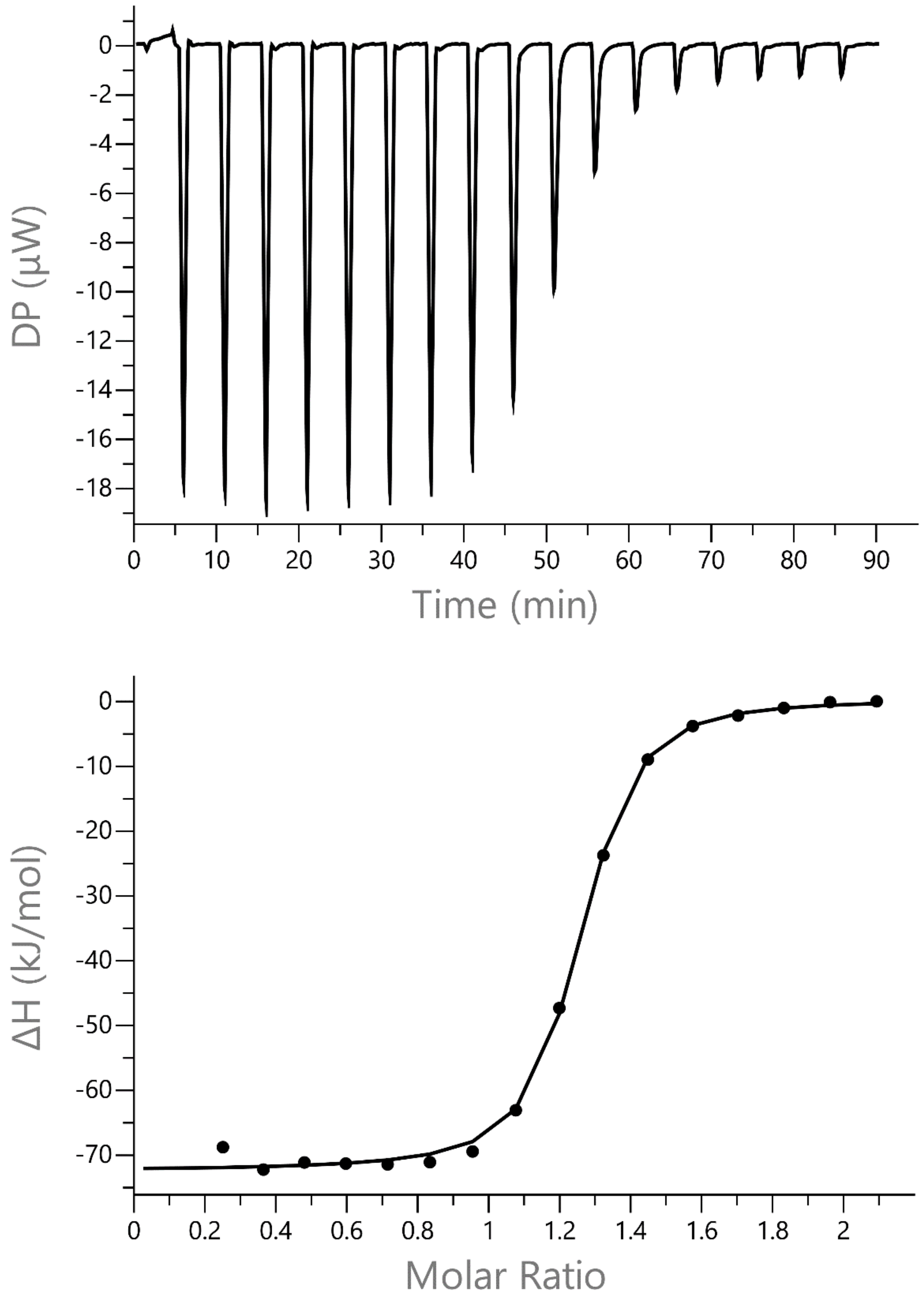

Figure S23: ITC data for titration of *N,N'*-diethylbenzamidine (0.5 mM) into benzoic acid (0.05 mM) in chloroform at 298 K. The raw data for each injection is shown (differential power, DP), along with the least-squares-fit of the enthalpy change per mole of guest (*ΔH*) to a 1:1 binding isotherm.

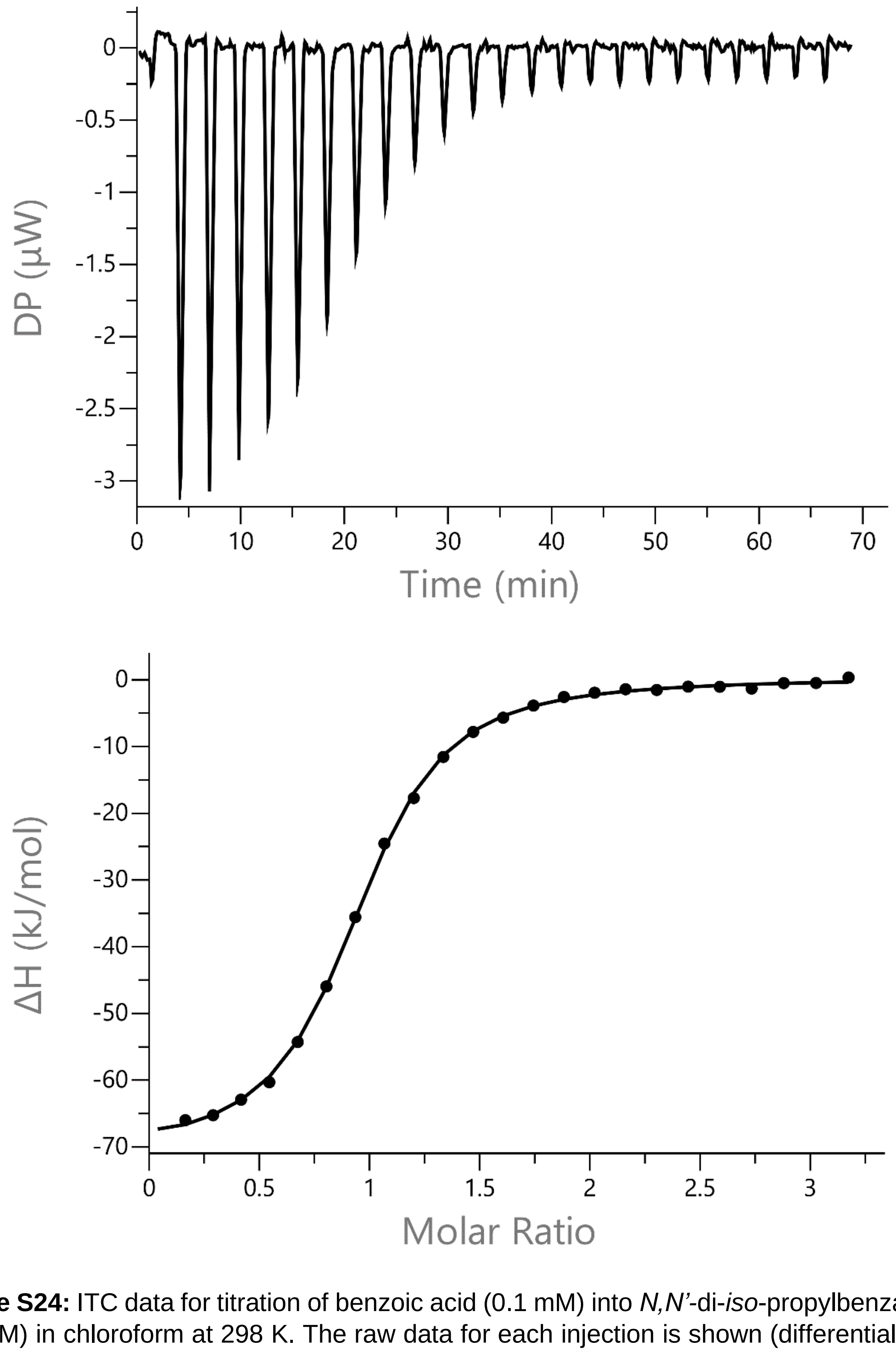

Figure S24: ITC data for titration of benzoic acid (0.1 mM) into *N,N'*-di-*iso*-propylbenzamidine (6.7 μM) in chloroform at 298 K. The raw data for each injection is shown (differential power, DP), along with the least-squares-fit of the enthalpy change per mole of guest (*ΔH*) to a 1:1 binding isotherm.

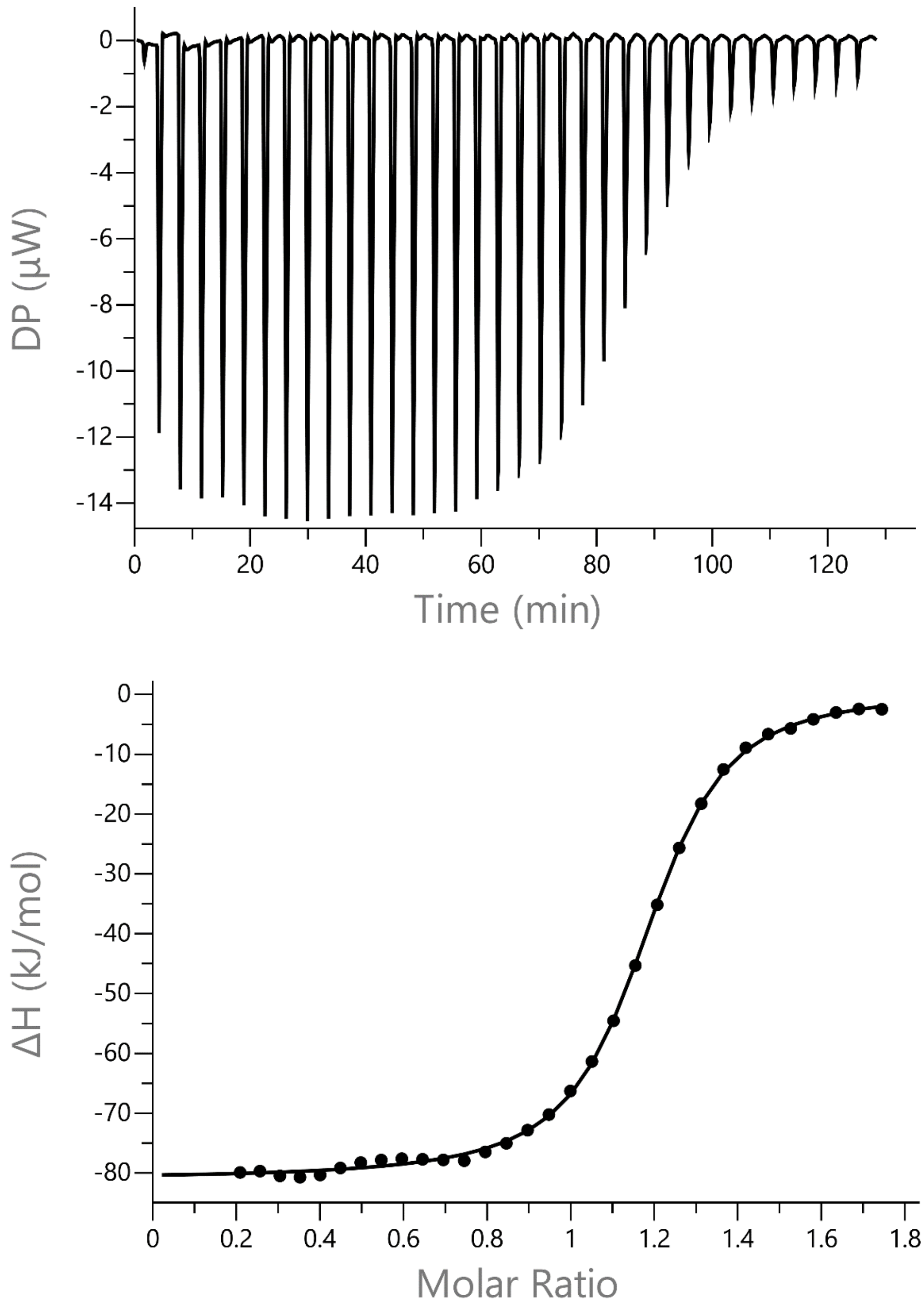

Figure S25: ITC data for titration of benzoic acid (0.5 mM) into *N,N'*-di-*tert*-butylbenzamidine (0.06 mM) in chloroform at 298 K. The raw data for each injection is shown (differential power, DP), along with the least-squares-fit of the enthalpy change per mole of guest (*ΔH*) to a 1:1 binding isotherm.

Solvent Effect Titrations

Formic acid and Benzamidine

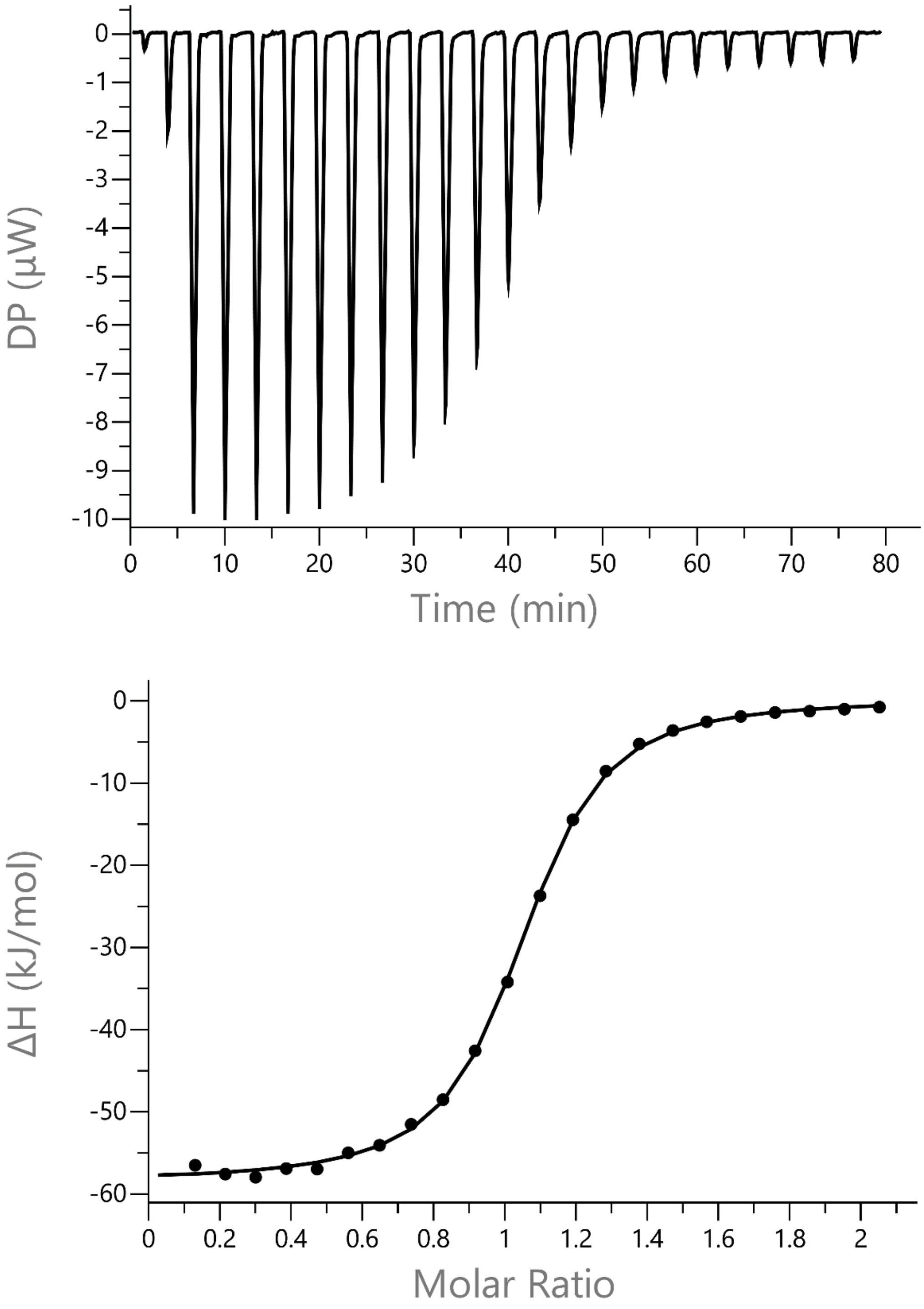

Figure S26: ITC data for titration of formic acid (0.4 mM) into benzamidine (0.04 mM) in chloroform at 298 K. The raw data for each injection is shown (differential power, DP), along with the least-squares-fit of the enthalpy change per mole of guest (*ΔH*) to a 1:1 binding isotherm.

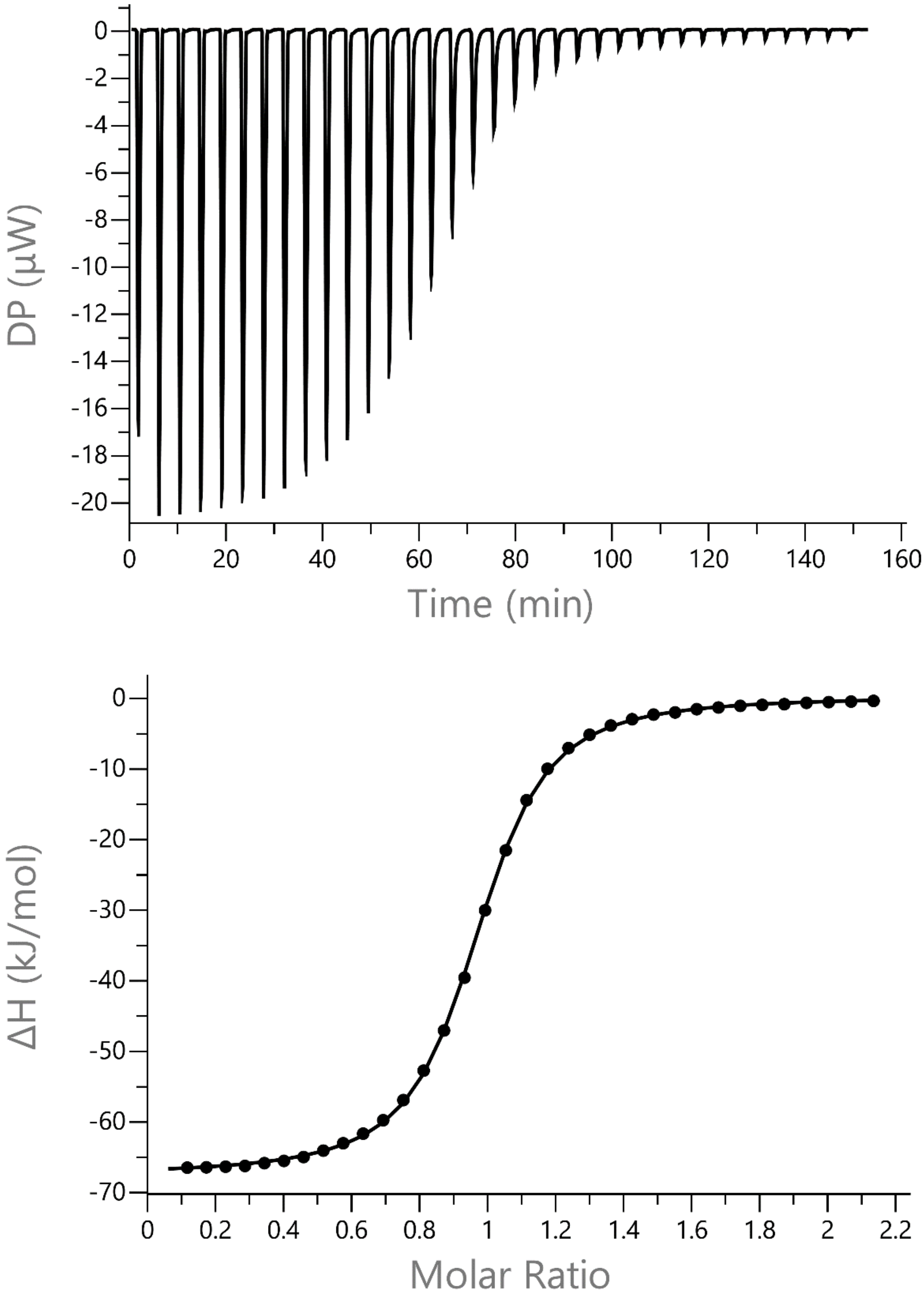

Figure S27: ITC data for titration of formic acid (1 mM) into benzamidine (0.1 mM) in PhOMe at 298 K. The raw data for each injection is shown (differential power, DP), along with the least-squares-fit of the enthalpy change per mole of guest (*ΔH*) to a 1:1 binding isotherm.

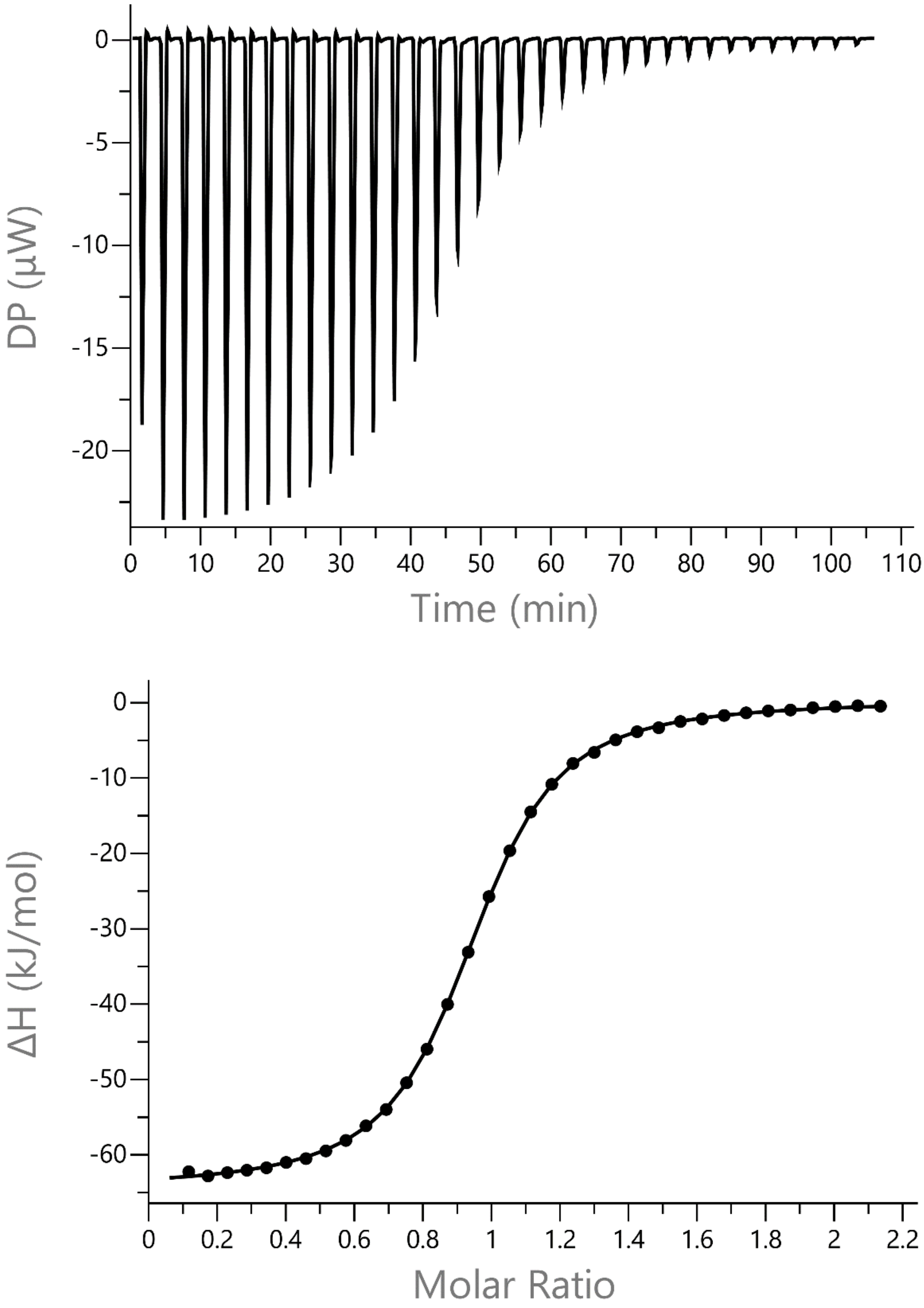

Figure S28: ITC data for titration of formic acid (1 mM) into benzamidine (0.1 mM) in MeCN at 298 K. The raw data for each injection is shown (differential power, DP), along with the least-squares-fit of the enthalpy change per mole of guest (*ΔH*) to a 1:1 binding isotherm.

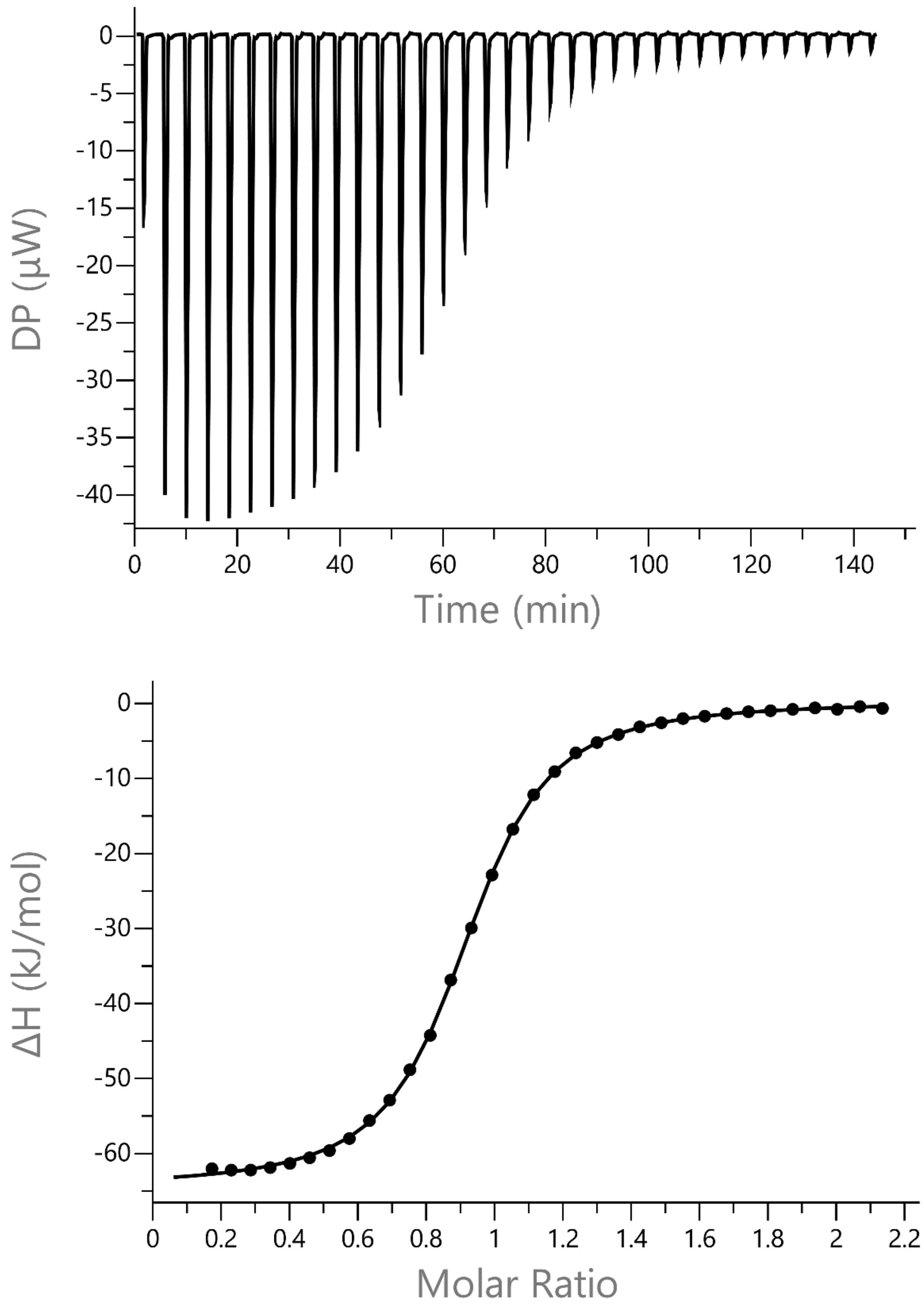

Figure S29: ITC data for titration of formic acid (2 mM) into benzamidine (0.2 mM) in acetone at 298 K. The raw data for each injection is shown (differential power, DP), along with the least-squares-fit of the enthalpy change per mole of guest (*ΔH*) to a 1:1 binding isotherm.

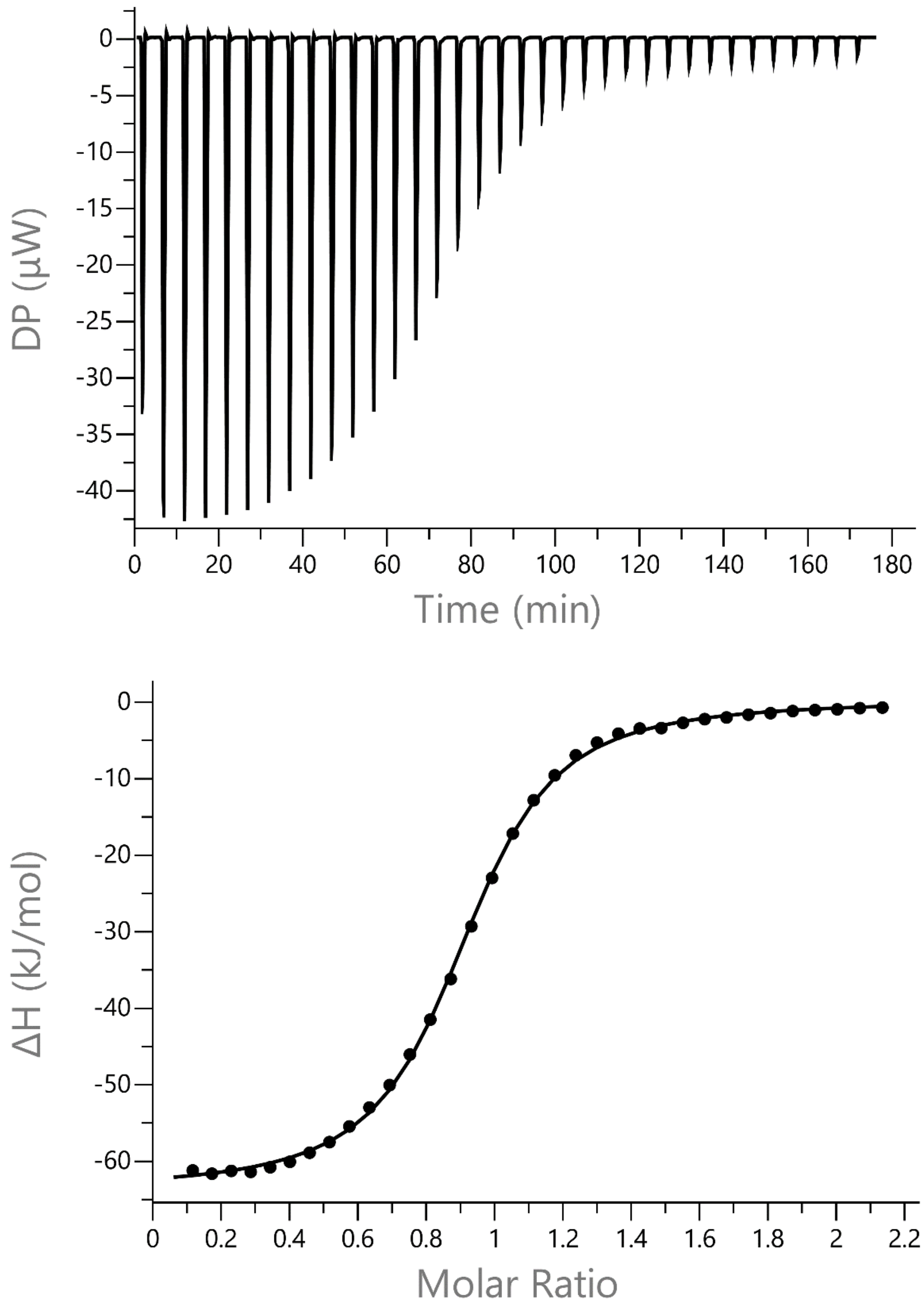

Figure S30: ITC data for titration of formic acid (2 mM) into benzamidine (0.2 mM) in EtOAc at 298 K. The raw data for each injection is shown (differential power, DP), along with the least-squares-fit of the enthalpy change per mole of guest (*ΔH*) to a 1:1 binding isotherm.

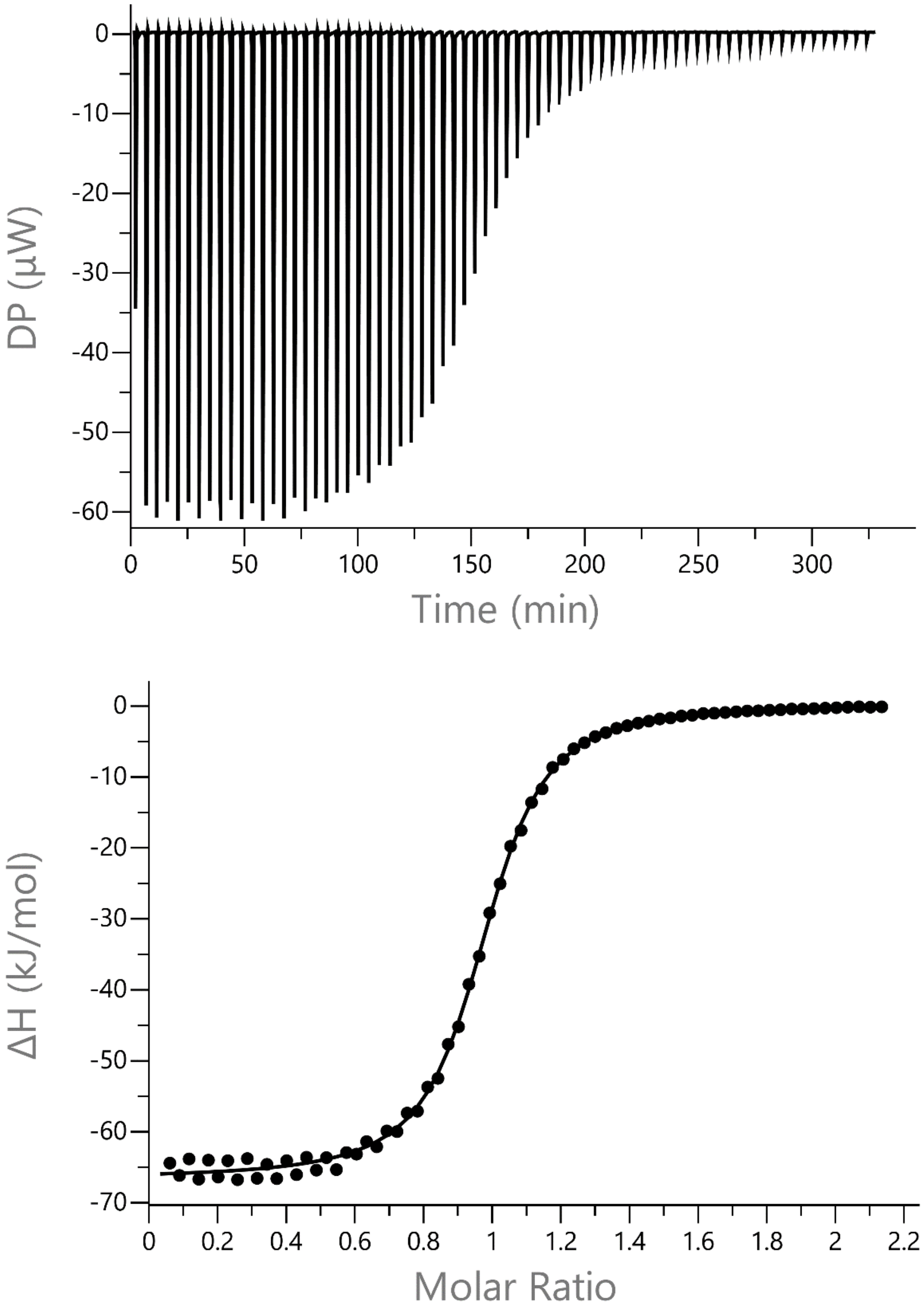

Figure S31: ITC data for titration of formic acid (5 mM) into benzamidine (0.5 mM) in DME at 298 K. The raw data for each injection is shown (differential power, DP), along with the least-squares-fit of the enthalpy change per mole of guest (*ΔH*) to a 1:1 binding isotherm.

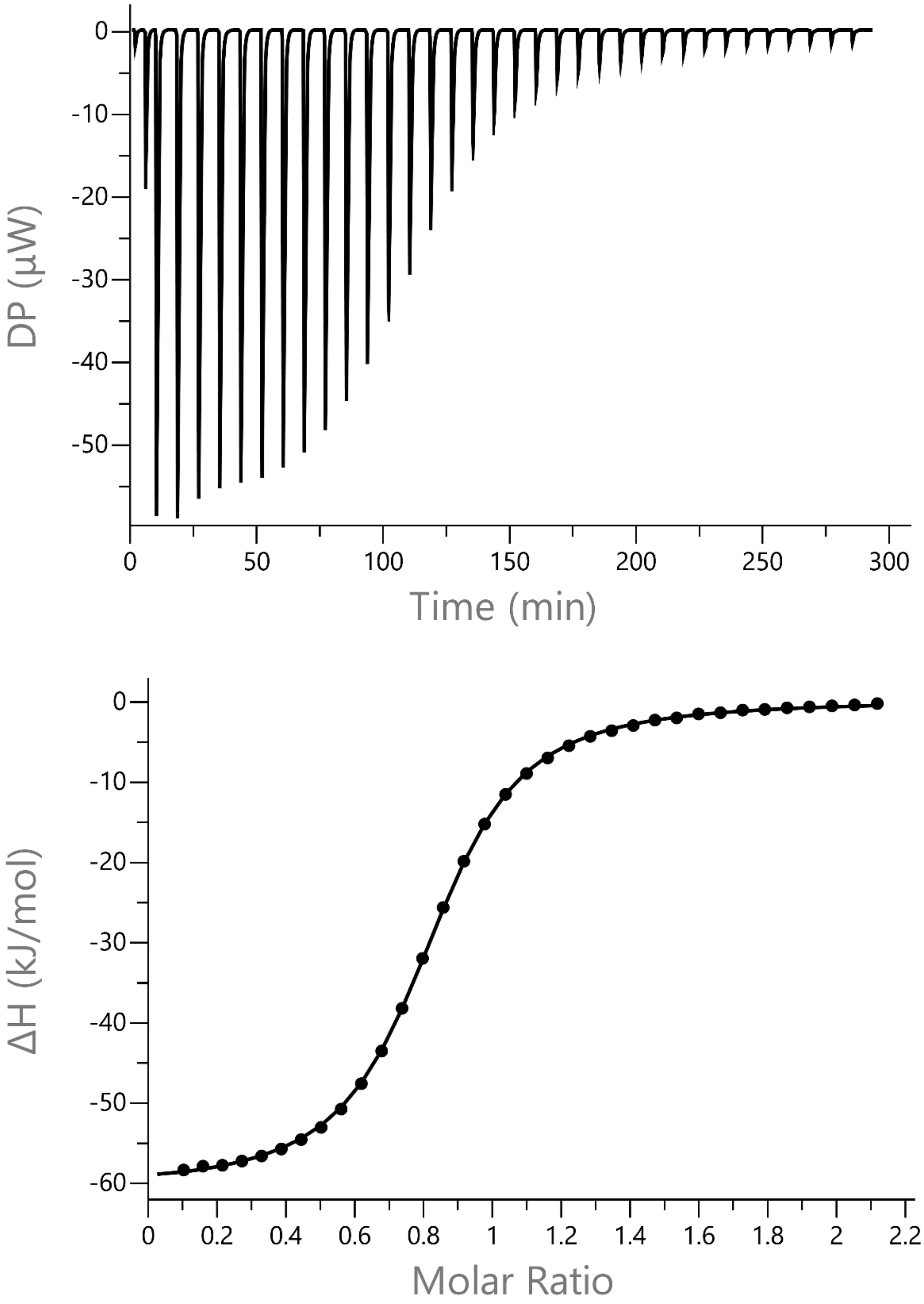

Figure S32: ITC data for titration of formic acid (5 mM) into benzamidine (0.5 mM) in THF at 298 K. The raw data for each injection is shown (differential power, DP), along with the least-squares-fit of the enthalpy change per mole of guest (ΔH) to a 1:1 binding isotherm.

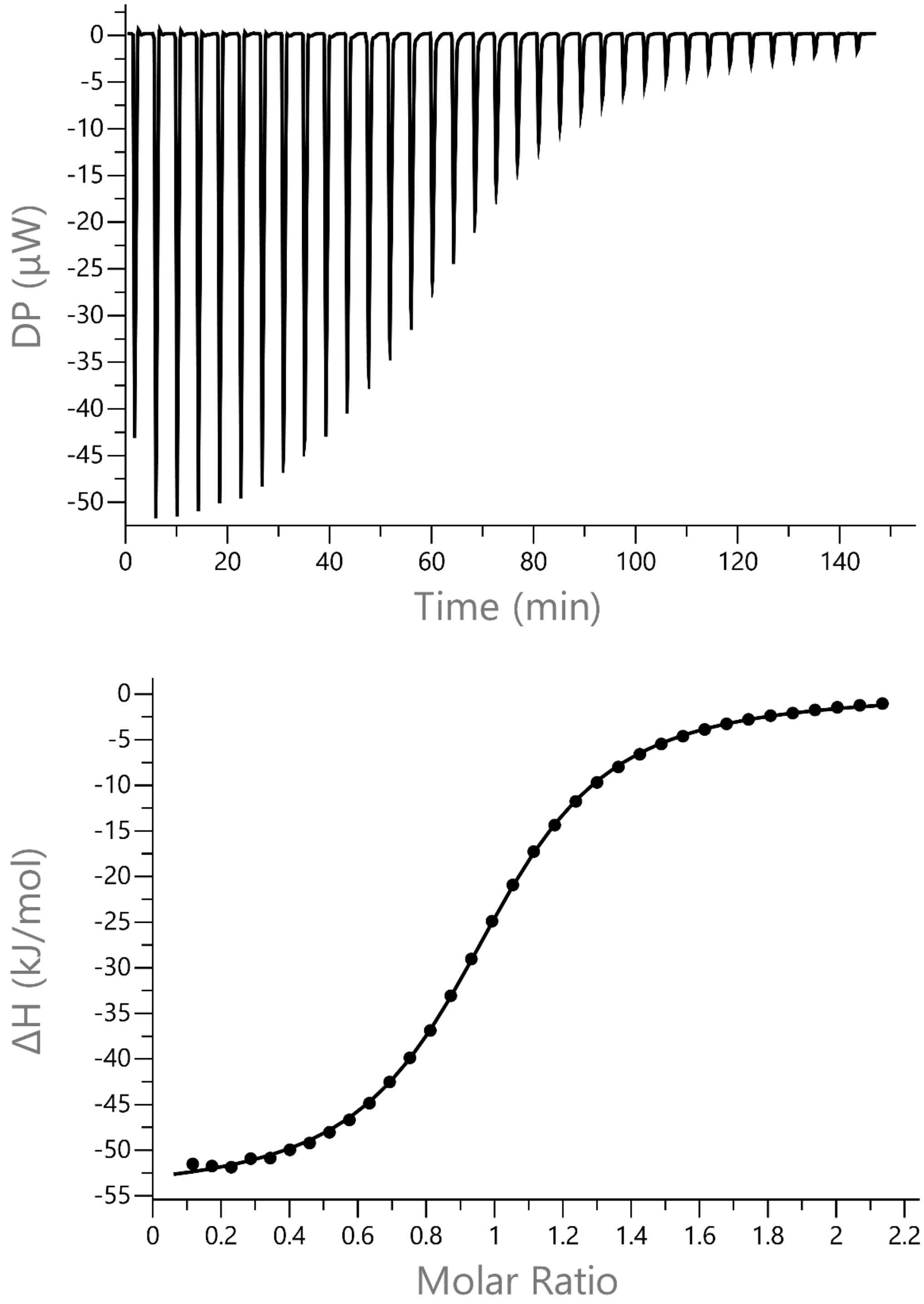

Figure S33: ITC data for titration of formic acid (3 mM) into benzamidine (0.3 mM) in DMF at 298 K. The raw data for each injection is shown (differential power, DP), along with the least-squares-fit of the enthalpy change per mole of guest (*ΔH*) to a 1:1 binding isotherm.

Formic Acid and *N,N'*-Dimethylbenzamidine

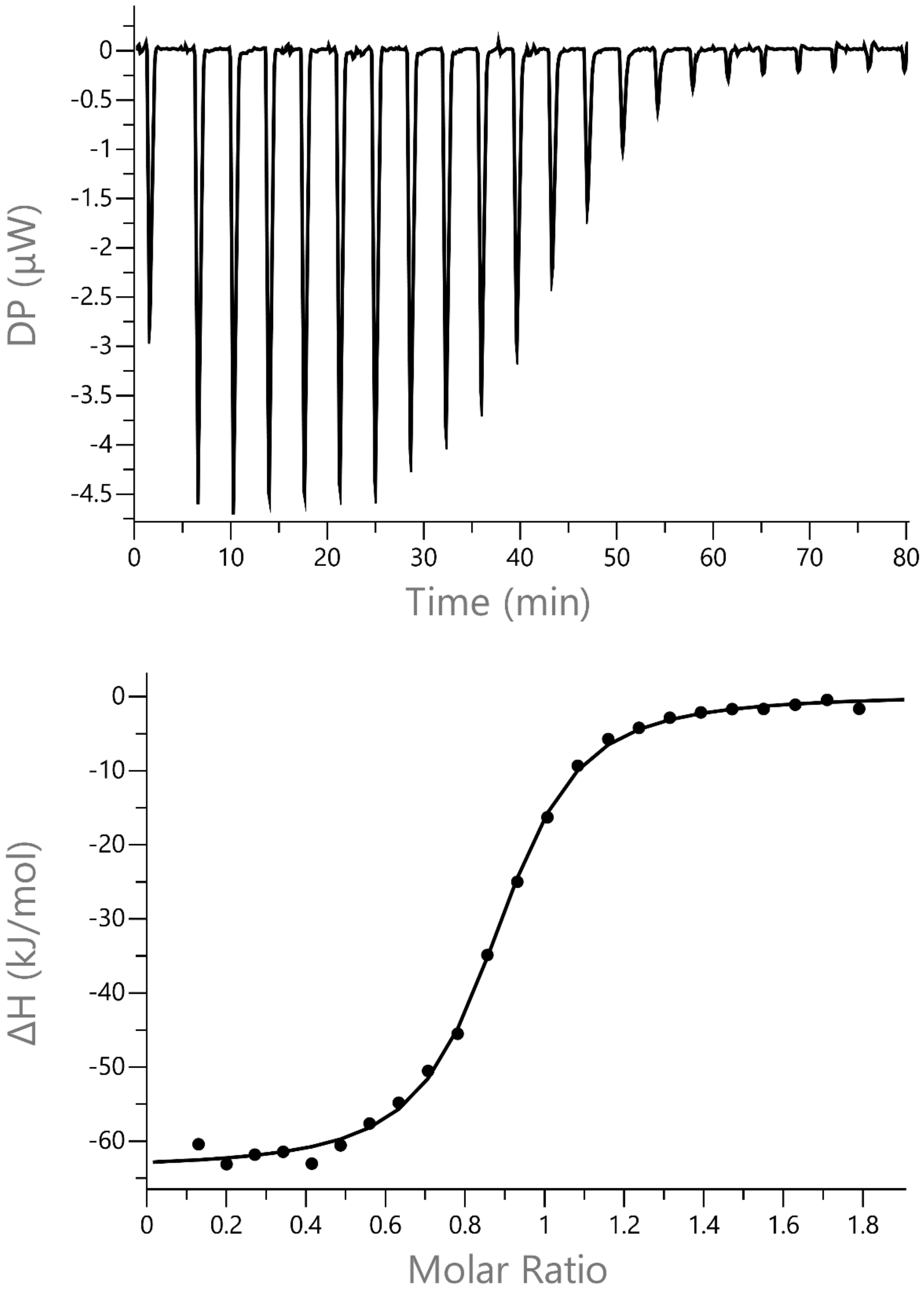

Figure S34: ITC data for titration of formic acid (0.2 mM) into *N,N'*-dimethylbenzamidine (0.02 mM) in chloroform at 298 K. The raw data for each injection is shown (differential power, DP), along with the least-squares-fit of the enthalpy change per mole of guest (*ΔH*) to a 1:1 binding isotherm.

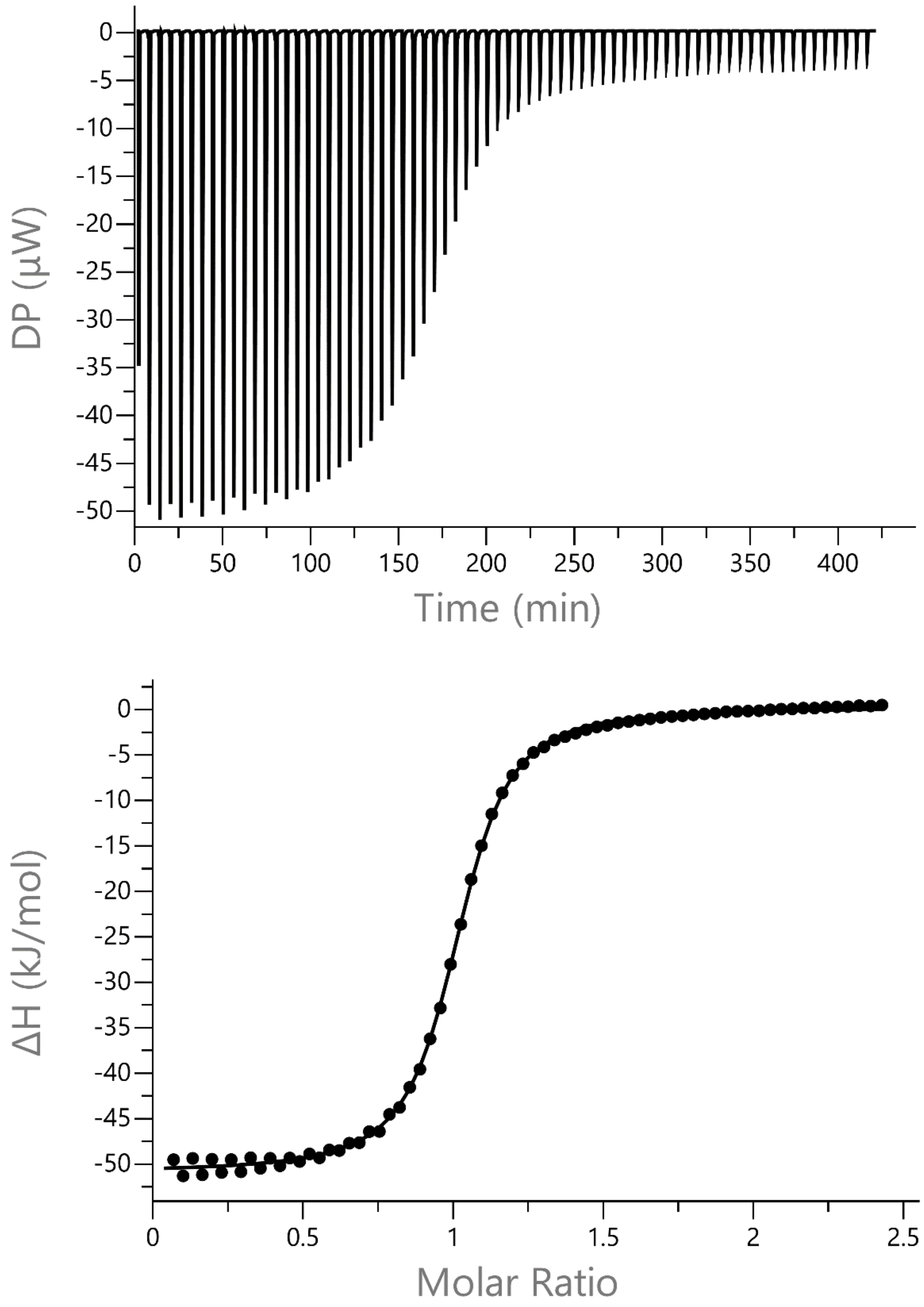

Figure S35: ITC data for titration of formic acid (5.7 mM) into *N,N'*-dimethylbenzamidine (0.5 mM) in PhOMe at 298 K. The raw data for each injection is shown (differential power, DP), along with the least-squares-fit of the enthalpy change per mole of guest (*ΔH*) to a 1:1 binding isotherm.

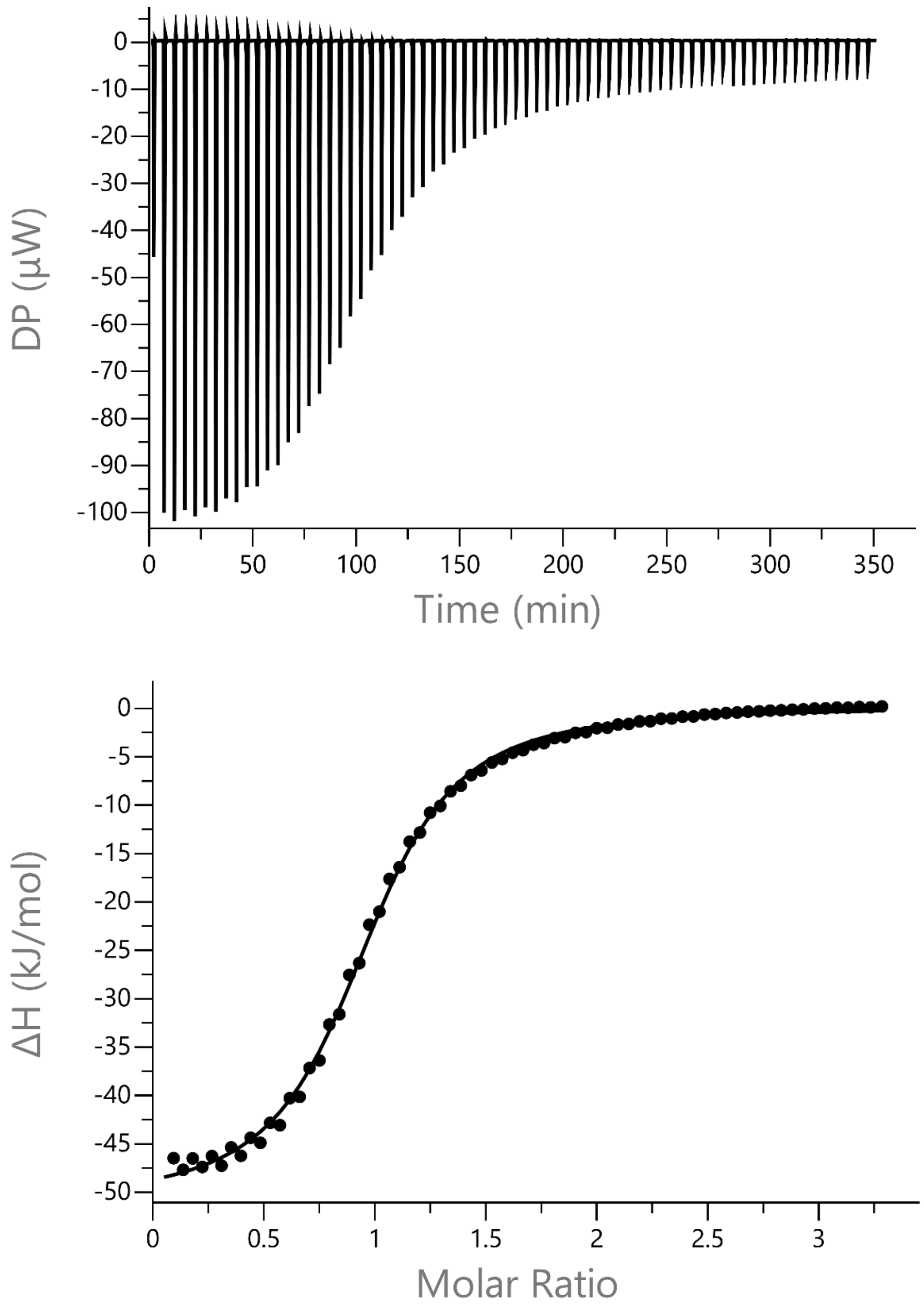

Figure S36: ITC data for titration of formic acid (10 mM) into *N,N'*-dimethylbenzamidine (0.65 mM) in MeCN at 298 K. The raw data for each injection is shown (differential power, DP), along with the least-squares-fit of the enthalpy change per mole of guest (*ΔH*) to a 1:1 binding isotherm.

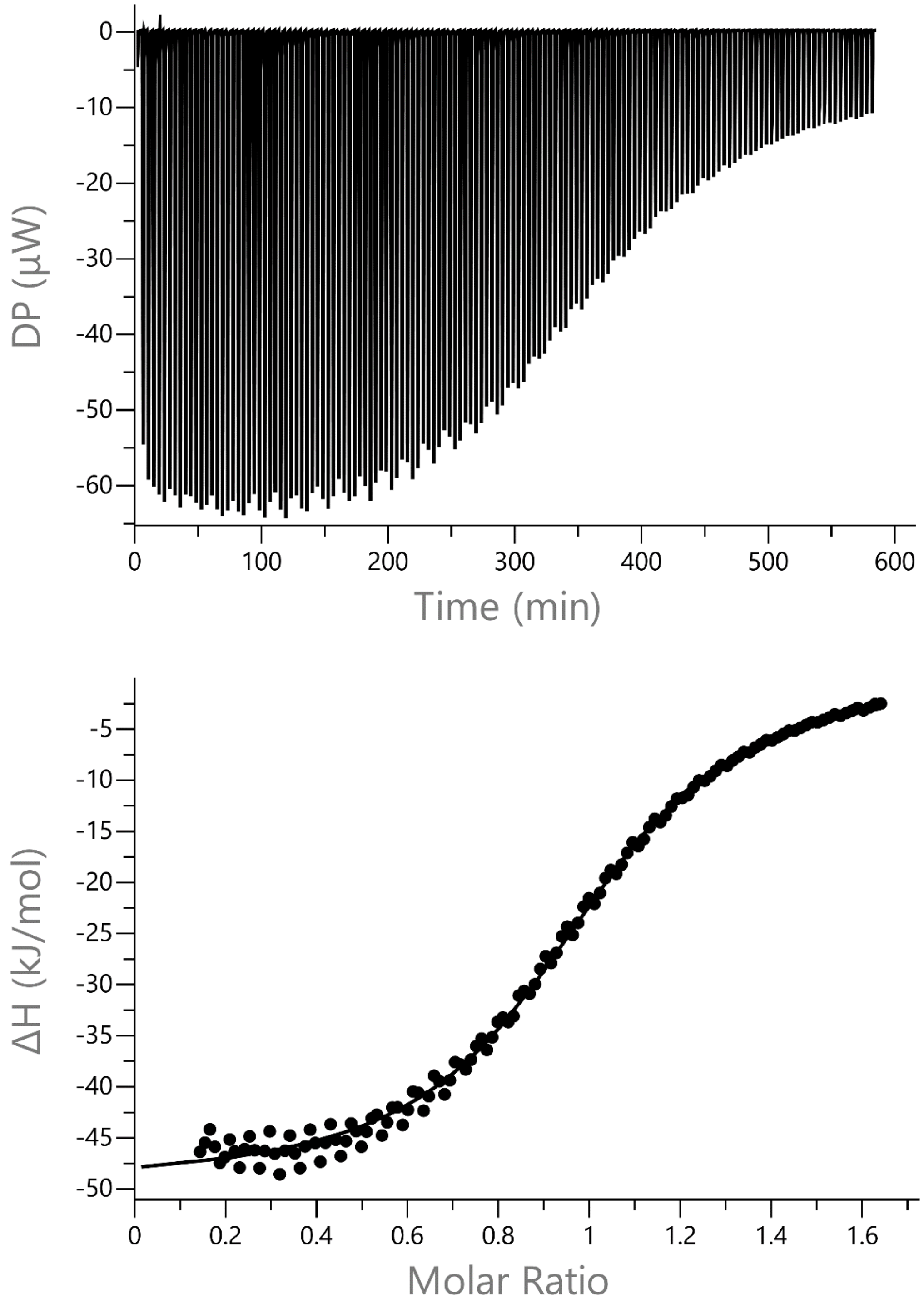

Figure S37: ITC data for titration of formic acid (18.4 mM) into *N,N'*-dimethylbenzamidine (2.3 mM) in Acetone at 298 K. The raw data for each injection is shown (differential power, DP), along with the least-squares-fit of the enthalpy change per mole of guest (*ΔH*) to a 1:1 binding isotherm.

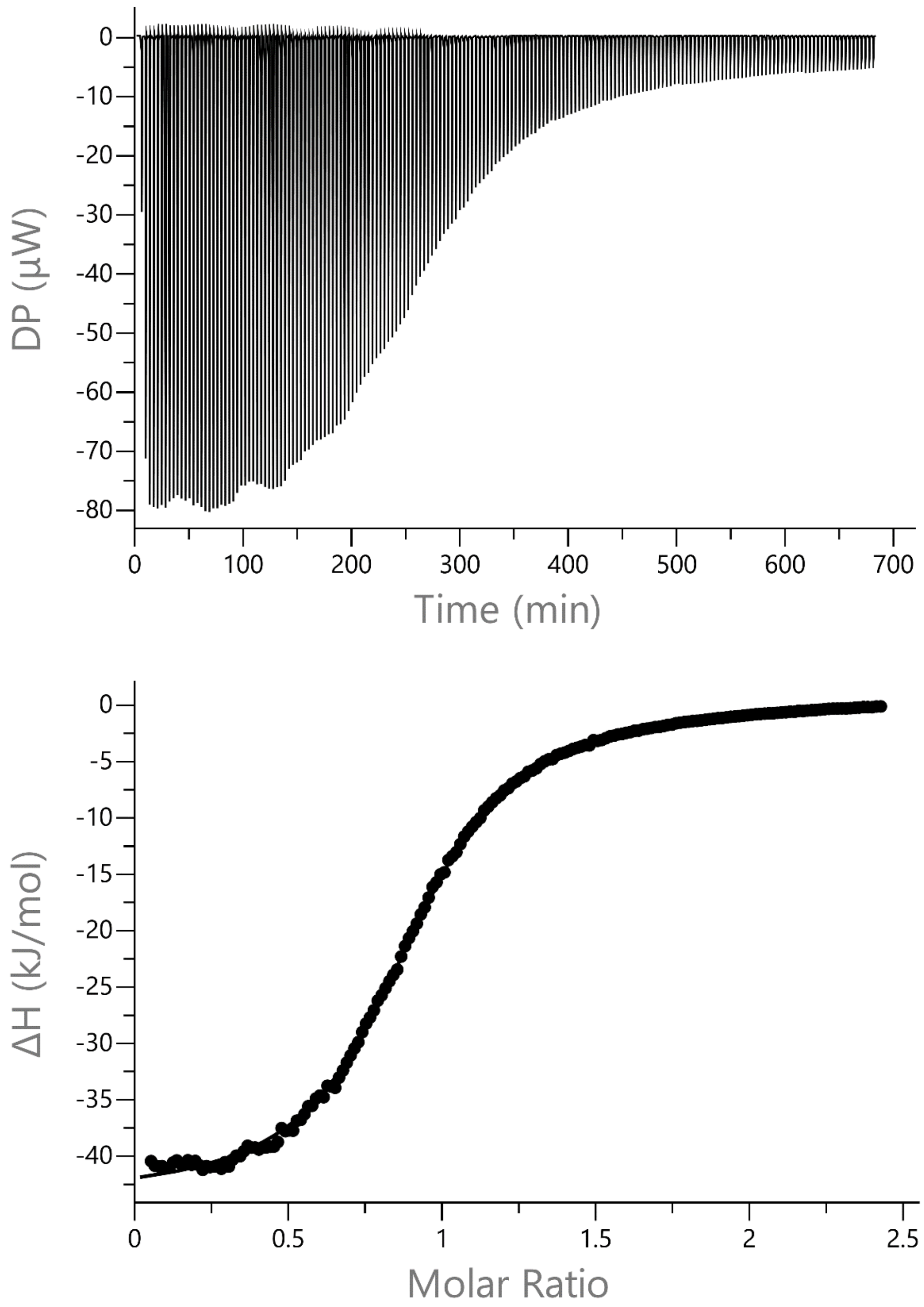

Figure S38: ITC data for titration of formic acid (27.4 mM) into *N,N'*-dimethylbenzamidine (2.4 mM) in EtOAc at 298 K. The raw data for each injection is shown (differential power, DP), along with the least-squares-fit of the enthalpy change per mole of guest (*ΔH*) to a 1:1 binding isotherm.

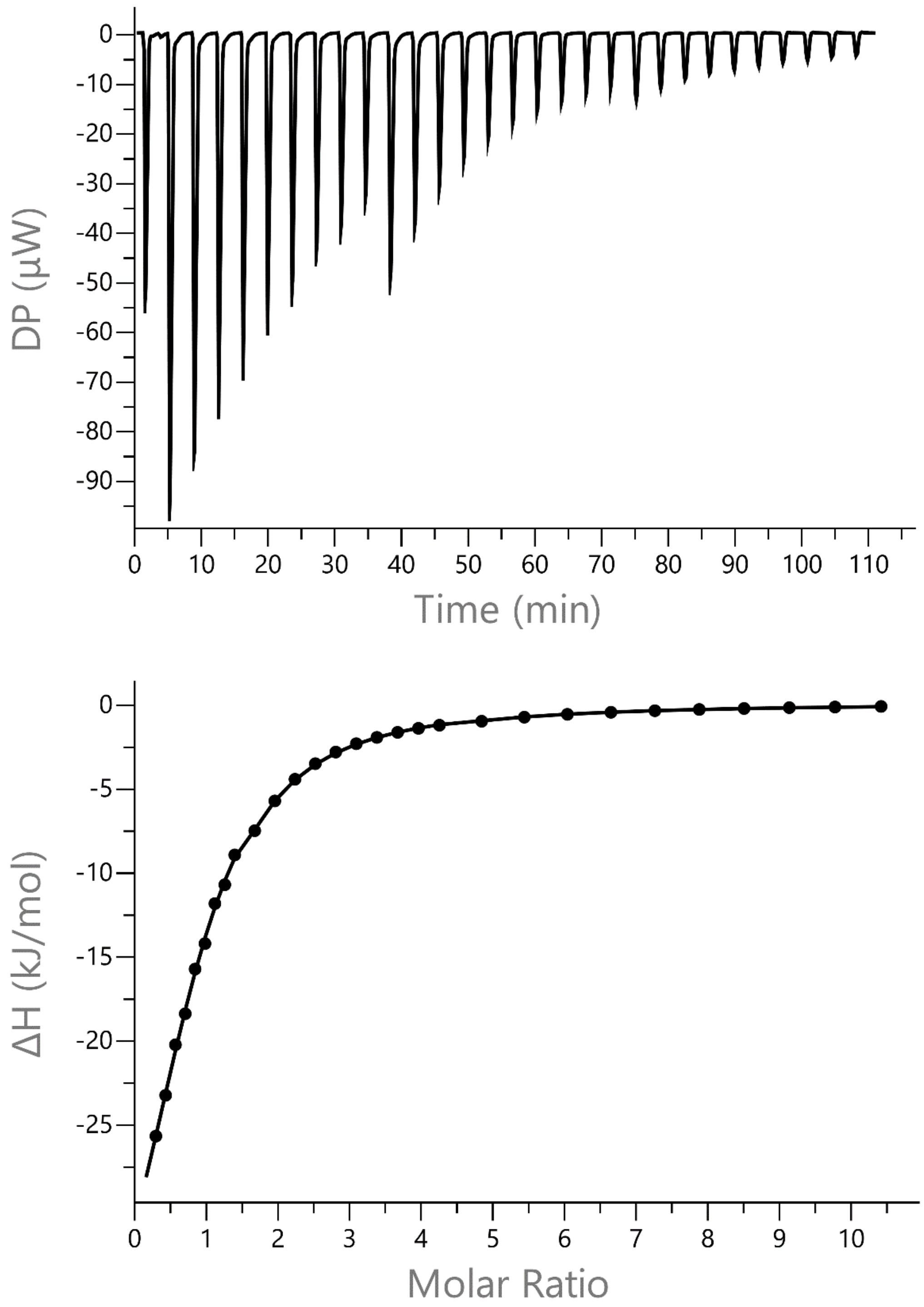

Figure S39: ITC data for titration of formic acid (24.4 mM) into *N,N'*-dimethylbenzamidine (0.5 mM) in DME at 298 K. The raw data for each injection is shown (differential power, DP), along with the least-squares-fit of the enthalpy change per mole of guest (*ΔH*) to a 1:1 binding isotherm. To decrease the duration of the experiment, the injection volume was increased from 4 µL to 8 µL after the first 10 injections.

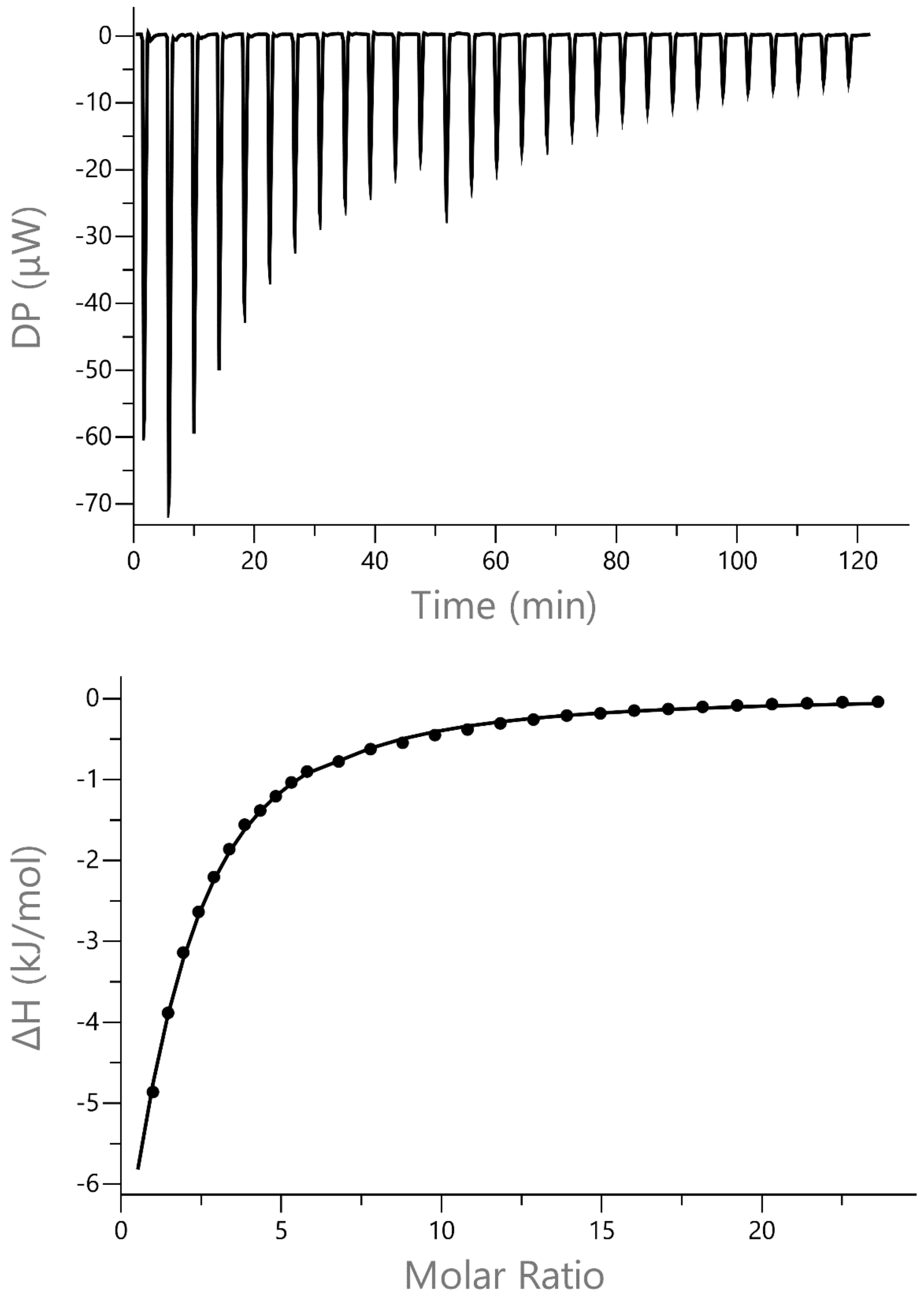

Figure S40: ITC data for titration of formic acid (56.2 mM) into *N,N'*-dimethylbenzamidine (0.5 mM) in THF at 298 K. The raw data for each injection is shown (differential power, DP), along with the least-squares-fit of the enthalpy change per mole of guest (*ΔH*) to a 1:1 binding isotherm. To decrease the duration of the experiment, the injection volume was increased from 6 μL to 12 μL after the first 12 injections.

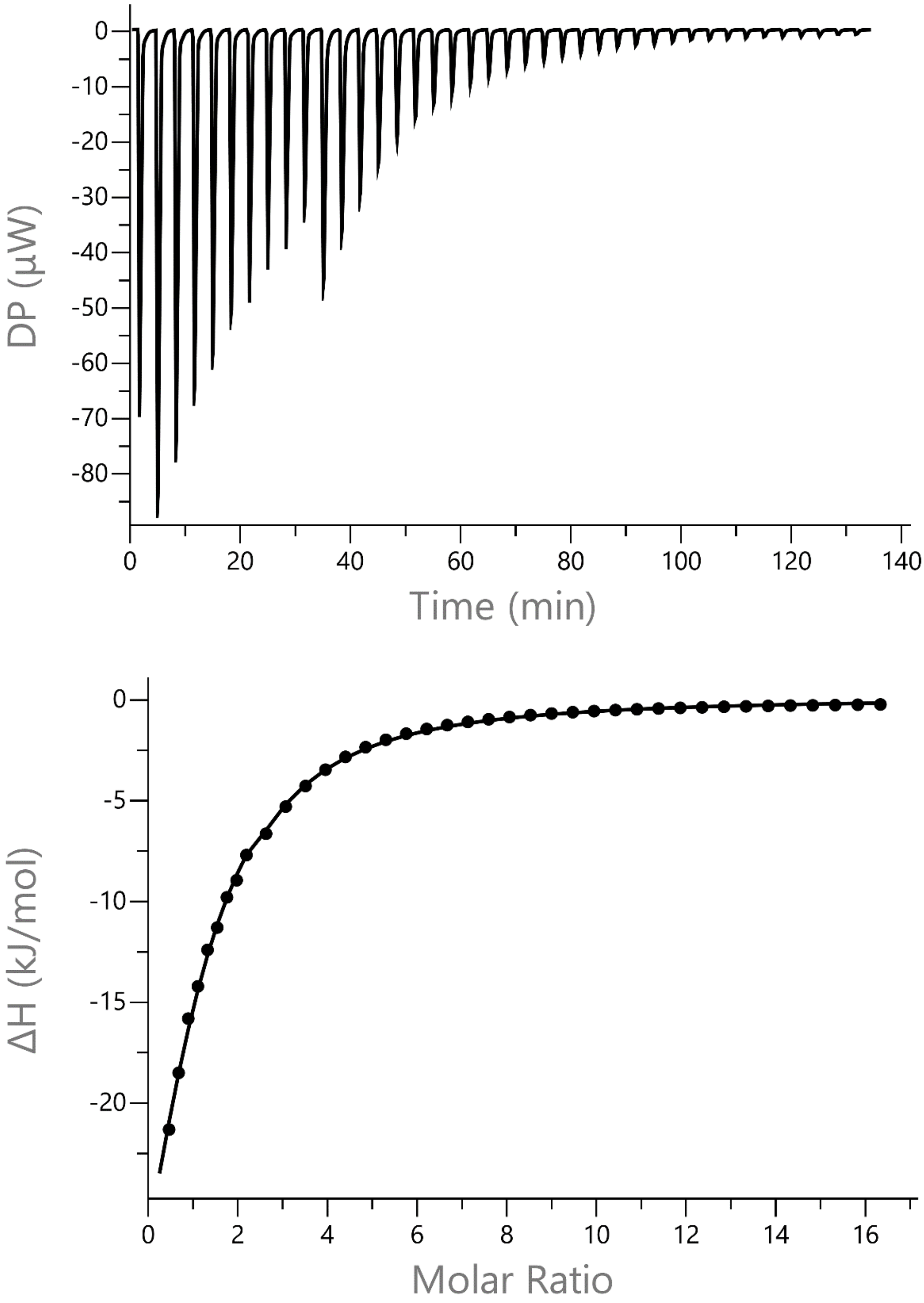

Figure S41: ITC data for titration of formic acid (30 mM) into *N,N'*-dimethylbenzamidine (0.5 mM) in DMF at 298 K. The raw data for each injection is shown (differential power, DP), along with the least-squares-fit of the enthalpy change per mole of guest (*ΔH*) to a 1:1 binding isotherm. To decrease the duration of the experiment, the injection volume was increased from 4 µL to 8 µL after the first 10 injections.

4. Diffusion-Ordered Spectroscopy Experiments

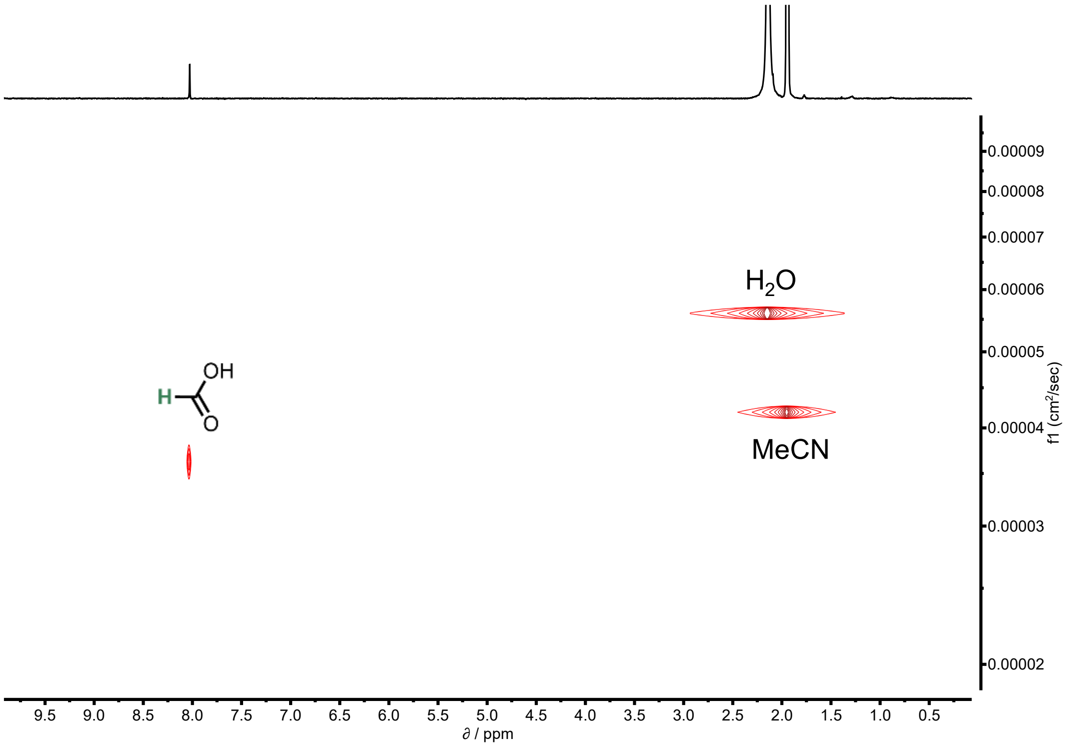

Figure S42: ^{1}H DOSY NMR (400 MHz, CD_3CN) of formic acid at 1 mM concentration.

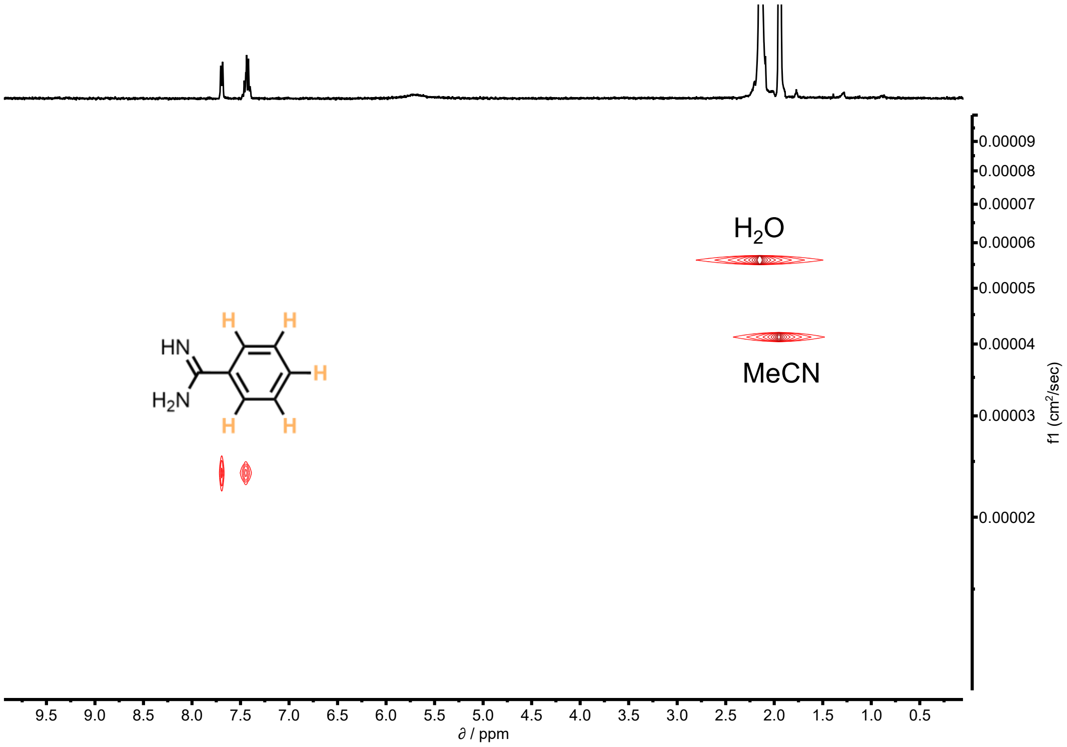

Figure S43: ^{1}H DOSY NMR (400 MHz, CD_3CN) of benzamidine at 1 mM concentration.

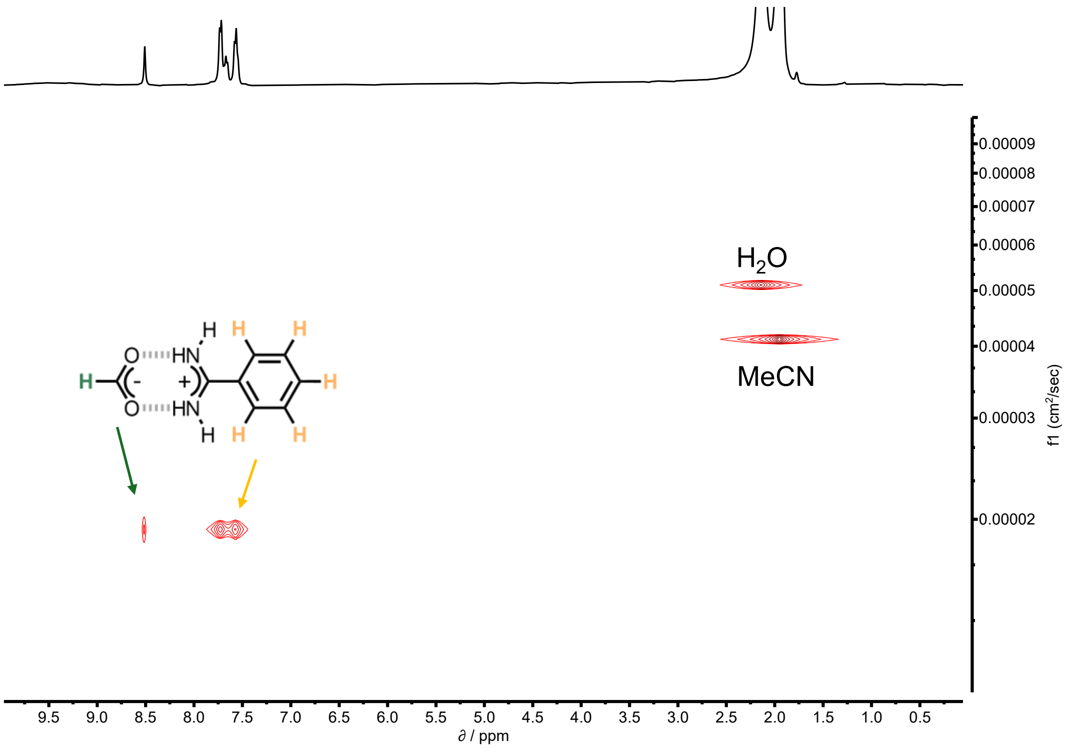

Figure S44: ^{1}H DOSY NMR (400 MHz, CD_3CN) of a 1:1 mixture of benzamidine (1 mM) and formic acid (1 mM).

Table S1: Diffusion coefficients measured in ^{1}H DOSY NMR experiments in CD_3CN at 298 K.[a]

	$\boldsymbol{D \times 10^9}$ **/ m^{2}s^{-1}**	
**^{1}H NMR Signal**	**Pure Compound**	**1:1 Mixture**
Formic Acid	3.80 ± 0.17	1.92 ± 0.04
Benzamidine	2.43 ± 0.10	1.94 ± 0.04

[a] Concentrations of all species were 1 mM, and errors are given as the largest of (i) the error in the fit or (ii) twice the standard deviation of fitted diffusion coefficients for peaks within the molecule.

5. Solvent Parameters

Solvent	ε_r	$E_T(30)$	δP	δH	α_s	β_s
$CHCl_3$	4.8	39.1	3.1	5.7	2.2	1.3
PhOMe	4.3	37.1	4.1	6.7	1.1*	3.3
MeCN	36.6	45.6	18.0	6.1	1.5	5.1
THF	7.6	37.4	5.7	8.0	0.9*	5.9
DMF	36.7	43.2	13.7	11.3	1.3*	7.4
DME	7.2	38.2	6.0	6.0	0.9*	5.3
Acetone	20.7	42.2	10.4	7.0	1.2	5.7
EtOAc	6.0	38.1	5.3	7.2	1.1*	5.3

Table S2: Solvent parameters: dielectric constant (ε_r),[S1] polarity ($E_T(30)$),[S1] Hansen polarity (δP) and hydrogen bonding (δH),[S2] and H-bond parameters (α_s and β_s) from experimental data [S3] or DFT calculations [S4] (asterisk).

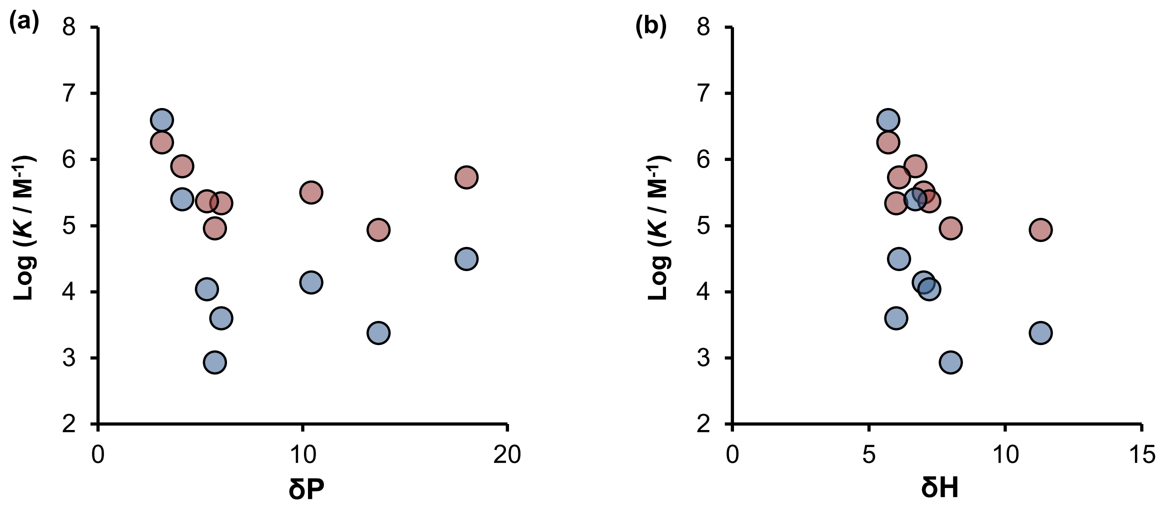

Figure S45: Comparison of the association constants for salt bridge formation between formic acid and benzamidine (red) or *N,N'*-dimethylbenzamidine (blue) with (a) Hansen polarity parameter (δP), and (b) Hansen hydrogen bonding parameter (δH).

6. Density Functional Theory Calculations

The structures of the molecules were optimised using Schrödinger's Maestro software (2016 Edition), with $CHCl_3$ as the implicit solvent and MMFFs as the forcefield. The result of this calculation was then used as a basis for a Jaguar DFT optimisation using a 6-31G** basis set and a B3LYP functional in the gas phase. The optimised structure was then used to calculate B3LYP/6-31G* molecular electrostatic potential surfaces using NWChem 7, and the results were used to calculate atomic surface site interaction point H-bond parameters as previously described.[S3]

7. References

(S1) Y. Marcus. *The Properties of Solvents*; Solutions Chemistry; Wiley, **1998**.

(S2) Hansen, C. M. *Hansen Solubility Parameters: A User's Handb*ook, Second Edition; CRC Press: Boca Raton, **2007**. https://doi.org/10.1201/9781420006834.

(S3) M. C. Storer and C. A. Hunter, *Chem. Soc. Rev.*, **2022**, 51, 10064.

(S4) M. C. Storer, K. J. Zator, D. P. Reynolds and C. A. Hunter, *Chem. Sci.*, **2024**, 15, 160-170.
